# Causal relationships between gut microbiota and lymphoma: a bidirectional Mendelian randomization study

**DOI:** 10.3389/fcimb.2024.1374775

**Published:** 2024-05-13

**Authors:** Jing Liang, Gengqiu Liu, Wenqing Wang, Hongman Xue

**Affiliations:** ^1^ Pediatric Hematology Laboratory, Division of Hematology/Oncology, Department of Pediatrics, The Seventh Affiliated Hospital of Sun Yat-Sen University, Shenzhen, Guangdong, China; ^2^ Department of Thoracic Surgery, The Seventh Affiliated Hospital, Sun Yat-sen University, Shenzhen, Guangdong, China

**Keywords:** gut microbiota, Hodgkin lymphoma, non-Hodgkin lymphoma, Mendelian randomization, causal association

## Abstract

**Background:**

Multiple studies have suggested a possible connection between the gut microbiota and the development of lymphoma, though the exact nature of this relationship remains unclear. This study aimed to explore whether a causal association exists between gut microbiota and lymphoma.

**Methods:**

A bidirectional two-sample Mendelian randomization (MR) approach was conducted to investigate potential causal effects between gut microbiota and various lymphoma subtypes. The primary method employed for MR analysis was inverse variance weighted (IVW), supplemented by additional methods including MR-Egger, weighted median, and weighted mode approaches. The Cochrane Q test, MR-PRESSO global test and MR-Egger intercept test were performed to assess pleiotropy and heterogeneity. Furthermore, a reverse MR analysis was performed to explore potential reverse causal effect.

**Results:**

The primary MR analysis identified 36 causal relationships between genetic liabilities in gut microbiota and different lymphoma subtypes. Neither the MR-PRESSO test nor the MR-Egger regression detected any pleiotropy, and Cochran’s Q test indicated no significant heterogeneity.

**Conclusions:**

Our MR analysis revealed substantial causal associations between gut microbiota and lymphoma, offering new insights into lymphoma prevention and management microbiota.

## Introduction

Lymphoma, a type of neoplasma characterized by significant heterogeneity, is commonly classified as Hodgkin lymphoma (HL) and non-Hodgkin lymphoma (NHL). These cancers are known for their varying degrees of immune evasion (Swerdlow et al., 2016). Over the past decade, changes in population growth and age structure have contributed to a continued increase in lymphoma incidence (Huang et al., 2022; Zhang et al., 2023). Despite significant advancements in treatments in recent years, the pathogenesis mechanisms of lymphoma remain incompletely elucidated. Currently, the lack of effective treatment options for refractory or drug-resistant lymphomas remains a persistent challenge (Brice et al., 2021; Bishop et al., 2022; Sehgal et al., 2022; Luan et al., 2024). Therefore, it is imperative to unravel the key mechanisms that govern tumor behavior and to develop clinically relevant biomarkers and therapeutic targets. These efforts aim to reduce the incidence of lymphoma and improve prognostic outcomes for patients.

Recently, the emerging paradigm of the microbiota-gut-lymphoma axis has been employed to explore potential correlations between the abundance of gut microbiota and a predisposition to lymphoma (Shi and Zhang, 2021; Upadhyay Banskota et al., 2023). Often described as our “second genome”, the gut microbiota plays a crucial role in shaping our immune response, educating it, and providing protection against pathogen overgrowth (Lynch and Pedersen, 2016). Its influence has been noted in conditions such as NHL and acute lymphoblastic leukemia (Rajagopala et al., 2016; Diefenbach et al., 2021; Shi et al., 2023). A classifier, developed using gut metagenomes for the natural killer/T-cell lymphoma cohort, achieved an accuracy of 0.813 area under the receiver operating characteristic curve (AUROC) in cross-validation (Shi et al., 2023). However, these studies primarily rely on analyzing the abundance and fluctuations of gut microbiota in patients’ fecal samples, and conducting experiments that involve transplanting microbiota into germ-free mice. Despite these advances, the precise correlation between gut microbiota and lymphoma remains indeterminate, underscoring the need for further research to thoroughly explore this relationship.

Mendelian randomization (MR) is a robust method that utilizes comprehensive data from genome-wide association study (GWAS) to investigate genetic associations. The main benefit of adopting this strategy lies in its capacity to effectively minimize the impact of confounders, including environmental variables, on the outcome. MR analysis involves using single nucleotide polymorphisms (SNPs), derived from independent GWAS, as instrumental variables (IVs). These SNPs are integrated with relevant health outcome data, facilitating the estimation of causal relationships within a unified framework. Additionally, this method enables the distinction between causal and non-causal associations using cross-sectional data (Burgess et al., 2015).

By employing a bidirectional two-sample MR analysis, we sought to investigate the causal association between gut microbiota and lymphoma, with the objective of providing novel insights into approaches for lymphoma prevention and management.

## Materials and methods

### Study design

**Figure 1:**
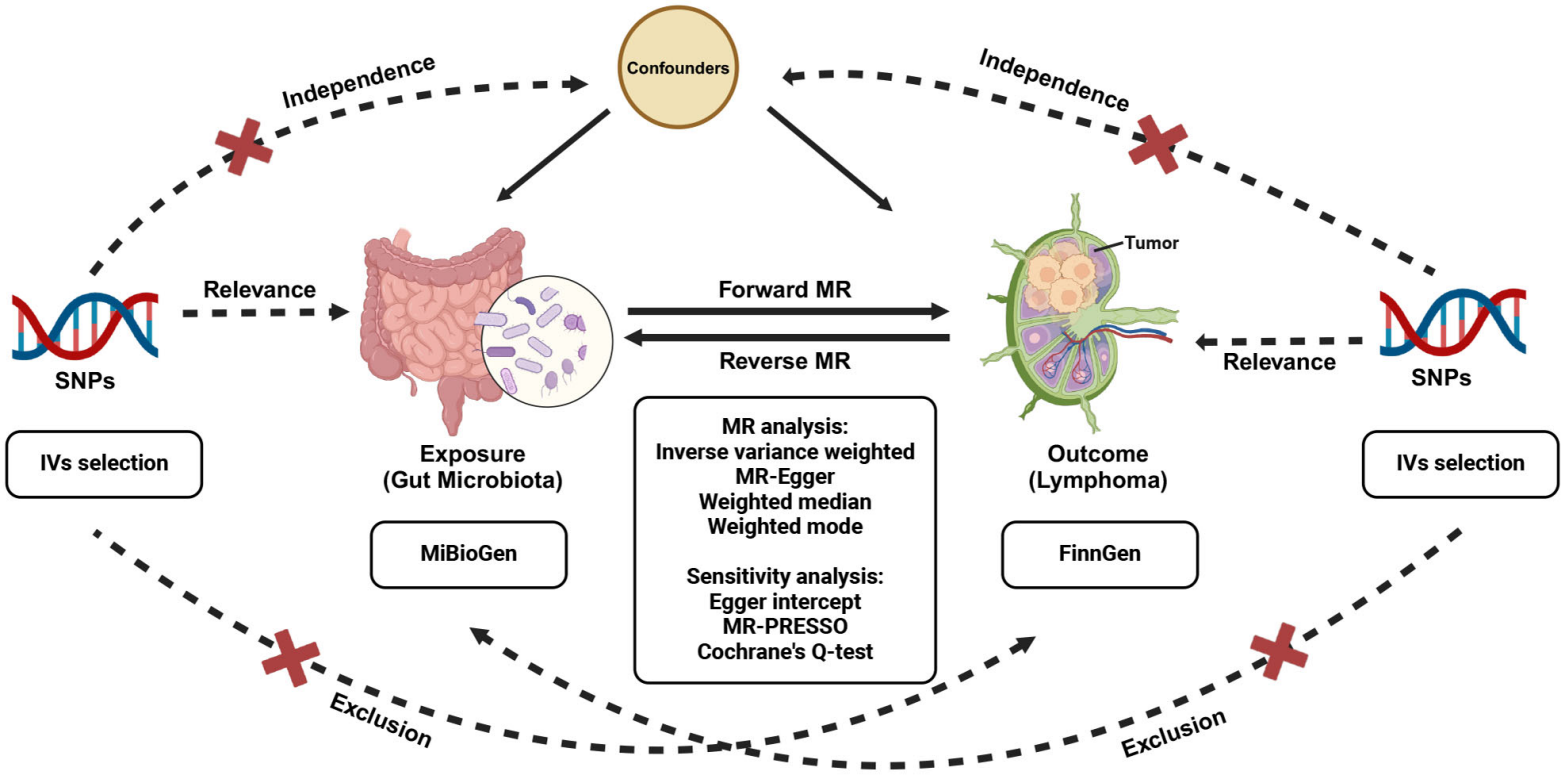
Study design of the bidirectional MR study of the correlation between gut microbiota and lymphoma. MR, Mendelian randomization; SNPs, single nucleotide polymorphisms.

Relevant GWAS summary data were employed to probe the plausible causal correlation between gut microbiota and malignant lymphoma, facilitated by a bidirectional two-sample MR analysis (Visscher et al., 2012). Initially, our study focus on determining whether gut microbiota exhibits a preventive or promotive role in lymphoma development. Moreover, we performed a reverse MR analysis to examine whether lymphoma might causally affect gut microbiota. The workflow of this study is underpinned by three fundamental IVs assumptions that support the primary MR analysis, as illustrated in **Figure 1**. To ensure the robustness of the findings, three hypotheses must be satisfied in the two-sample MR (Bowden et al., 2015): (1) Relevance, demonstrating a significant association between genetic variations and exposure; (2) Independence, ensuring no relationship between genetic variants and confounding factors; and (3) Exclusion, stipulating that the genetic variants influence the outcome solely through exposure, without involving any other pathways. Genetic variants that fulfill these three hypotheses can be utilized as IVs.

### Data selection

#### Gut microbiota GWAS

Information was obtained from an exhaustive microbiome GWAS conducted by the MiBioGen consortium (Kurilshikov et al., 2021). This multi-ethnic GWAS comprised 18,340 individuals from 24 study cohorts. The analysis demonstrated a connection between autosomal human genetic variants and gut microbiota, taking into account variables such as age, gender, study-specific factors, and genetic principal components, utilizing profiles obtained through 16S ribosomal RNA gene sequencing. Our analysis encompassed a total of 196 taxa, consisting of 119 genera, 32 families, 20 orders, 16 classes and 9 phyla, excluding those unable to be definitively classified or named. Detailed information was shown in **Supplementary Table S1**.

#### Lymphoma GWAS

We retrieved data on lymphomas from the FinnGen database (https://www.finngen.fi/en). The GWAS for HL comprised 780 cases and 299952 controls. For NHL, the GWAS data covered various subtypes: diffuse large B-cell lymphoma (DLBCL) with 1010 cases and 287137 controls, follicular lymphoma (FL) with 1081 cases and 299952 controls, mature T/NK-cell lymphomas with 335 cases and 299952 controls, and other and unspecified types of NHL with 1088 cases and 299952 controls. This comprehensive categorization provides more accurate and extensive data on genetic variances, aiding our exploration of causal associations between gut microbiota and different malignant lymphoma pathological subtypes within our MR investigation. Detailed information can be found in **Supplementary Table S1**.

#### Selection of eligible IVs

To guarantee the accuracy and authenticity of our findings on the possible association between gut microbiota and lymphoma susceptibility, we employed a range of quality control measures to identify optimal independent IVs. First, SNPs selected to serve as IVs were required to exhibit a significant correlation with the gut microbiota. To explore potential causal associations, we adopted a locus-wide significance threshold at *p* = 1 × 10^-5^, consistent with thresholds frequently used in previous analyses (Liu et al., 2022; Lopera-Maya et al., 2022). Additionally, in reverse MR analysis, we employed a lenient genome-wide significance threshold at *p* = 5 × 10^-6^ to identify SNPs correlated with lymphoma (Su et al., 2023; Xie et al., 2023). Second, to mitigate potential biases due to strong linkage disequilibrium (LD), we conducted an LD analysis with a threshold set at *r²* < 0.001 and a clumping distance of 10,000 kb, employing the 1000 Genomes Project European samples as a reference panel (Purcell et al., 2007). Third, to minimize any potential confounders, each SNP was assessed in the PhenoScanner website (Kamat et al., 2019). Fourth, GWAS summary data for the chosen SNPs were retrieved from the outcome dataset, and SNPs strongly associated with the outcome (*p* < 5 × 10^-5^) were excluded. Fifth, to evaluate the potential influence of horizontal pleiotropy, we performed MR-Egger regression tests and MR-PRESSO analyses. Simultaneously, we excluded palindromic SNPs to avoid potential biases related to strand orientation or allele coding, and removed ambiguous and duplicated SNPs. Finally, IVs were omitted if the *F*-statistic fell below 10, calculated utilizing the subsequent equation: *F* = *R^2^* (*n* – *k* – 1)/*k*(1 – *R^2^*). Here *R^2^* denotes the proportion of variance accounted for by all SNPs, *n* stands for the total sample size, and *k* denotes the number of SNPs.

### Mendelian randomization analyses

To investigate causality, the inverse variance weighted (IVW) method was employed as the principal approach to synthesize effect estimates (Burgess et al., 2013). Supplemental calculations were conducted utilizing various methods, among them MR-Egger, weighted mode, and weighted median, each accounting for varying assumptions regarding potential pleiotropy (Bowden et al., 2015, 2016). The consistency of results from these complementary methods with the IVW estimates enhances the credibility of our findings. For significance evaluation, a Bonferroni correction was applied, setting the significance thresholds for each taxonomic level by dividing 0.05 by the total number of independent bacterial taxa present at each level: phylum (*p* < 5.6 × 10^–3^), class (*p* < 3.1 × 10^–3^), order (*p* < 2.5 × 10^–3^), family (*p* < 1.6 × 10^–3^), and genus (*p* < 4.2 × 10^–4^). Additionally, *p*-values that fell between the established significance threshold and 0.05 were interpreted as suggestive of a potential causal relationship.

We calculated the heterogeneity statistic Q to assess effect estimates. Outlier SNPs were identified using the MR pleiotropy residual sum and outlier (MR-PRESSO) method (Verbanck et al., 2018). Furthermore, we implemented the leave-one-out technique to evaluate the potential influence of a single instrument on our MR findings.

We performed all analyses using the statistical software R (version 4.2.2), employing the TwoSampleMR (version 0.5.6) and MR-PRESSO (version 1.0) packages.

### Ethical approval

The summary datasets are freely accessible through OPEN GWAS. We utilized data from participating studies that had received ethical clearance from committees overseeing human experimentation standards. This eliminated the need for additional ethical approval for this study.

## Results

### Instrumental variables selection

In our analysis, we initially identified appropriate IVs based on predefined criteria. Details about the SNPs utilized in the two-sample MR analysis can be found in **Supplementary Table S2**-**S6**. After data harmonization, we determined that more than one SNP was associated with each bacterial taxon and lymphoma subtype. Furthermore, the *F*-statistics for all selected SNPs exceeded 10, alleviating concerns about the strength of the IVs.

### MR analysis

#### The causal effects of gut microbiota on lymphoma

Four MR methods were employed to investigate the causal associations between specific bacterial taxa and various lymphoma subtypes, as illustrated in **Supplementary Table S7**-**S11**.

**Figure 2:**
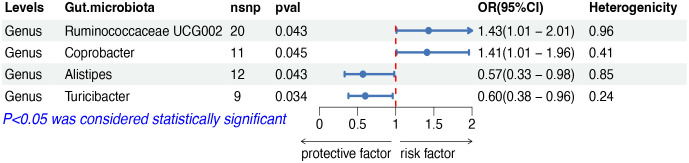
MR results of causal effects between gut microbiota and DLBCL. MR, Mendelian randomization; DLBCL, diffuse large B-cell lymphoma; nsnp, number of single nucleotide polymorphism; OR, odds ratio; CI, confidence interval.

The IVW analysis indicated that four gut microbiota taxa had causal effects on DLBCL, as illustrated in **Figure 2**. We found that the genus Ruminococcaceae UCG002 (odds ratio (OR): 1.43, 95% confidence interval (CI): 1.01–2.01, *p* = 0.043) and the genus Coprobacter (OR: 1.41, 95% CI: 1.01–1.96, *p* = 0.044) were positively correlated with the risk of DLBCL. On the contrary, the genus Alistipes (OR: 0.57, 95% CI: 0.33–0.98, *p* = 0.043) and the genus Turicibacter (OR: 0.60, 95% CI: 0.38–0.96, *p* = 0.034) were negatively correlated with DLBCL risk. Both weighted median and weighted mode analyses demonstrated consistent trends in ORs. Visual representations of the causal relationships between significant bacteria and DLBCL are demonstrated in scatter plots (**Supplementary Figure S1**).

**Figure 3:**
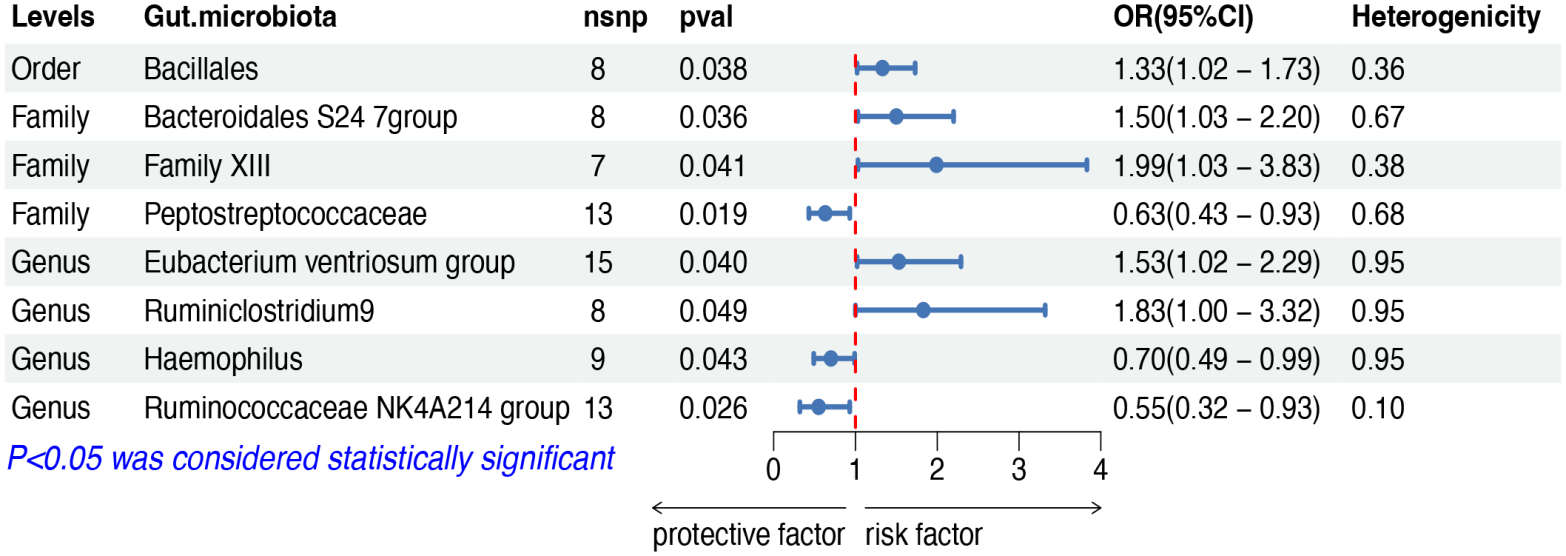
MR results of causal effects between gut microbiota and FL. MR, Mendelian randomization; FL, follicular lymphoma; nsnp, number of single nucleotide polymorphism; OR, odds ratio; CI, confidence interval.

The IVW analysis indicated that eight gut microbiota taxa had causal effects on FL, as illustrated in **Figure 3**. We found that the order Bacillales (OR: 1.32, 95% CI: 1.02–1.73, *p* = 0.038), the family Bacteroidales S24 7group (OR: 1.50, 95% CI: 1.03–2.20, *p* = 0.036), the family Family XIII (OR: 1.99, 95% CI: 1.03–3.83, *p* = 0.040), the genus Eubacterium ventriosum group (OR: 1.53, 95% CI: 1.02–2.29, *p* = 0.040) and the genus Ruminiclostridium9 (OR: 1.83, 95% CI: 1.00–3.32, *p* = 0.048) were positively correlated with the risk of FL, while the family Peptostreptococcaceae (OR: 0.63, 95% CI: 0.43–0.93, *p* = 0.019), the genus Haemophilus (OR: 0.70, 95% CI: 0.49–0.99, *p* = 0.049) and the genus Ruminococcaceae NK4A214 group (OR: 0.55, 95% CI: 0.32–0.93, *p* = 0.025) showed a negative correlation with FL risk. Both the weighted median and weighted mode demonstrated consistent trends in ORs. Visual representations of the causal relationships between significant bacteria and FL are demonstrated in scatter plots (**Supplementary Figure S1**).

**Figure 4:**
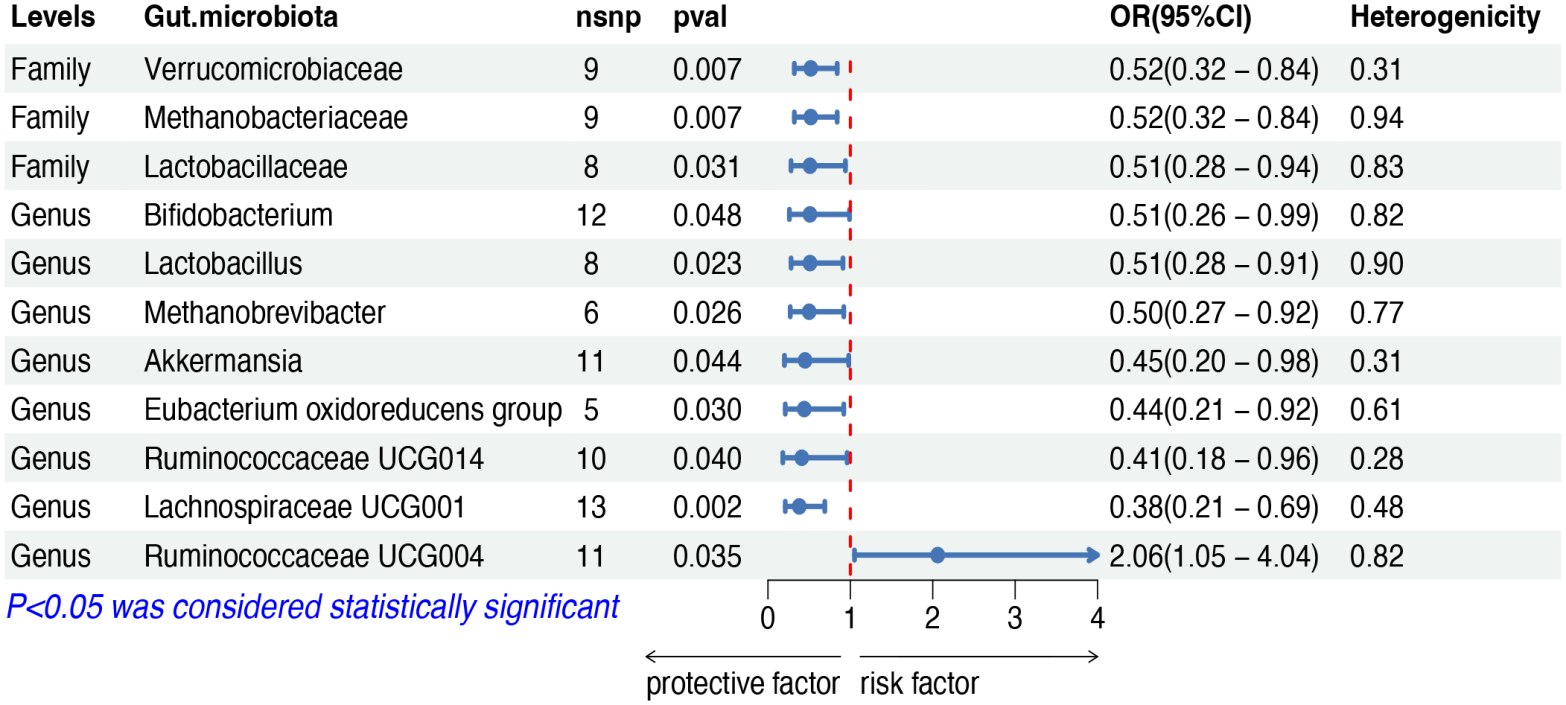
MR results of causal effects between gut microbiota and mature T/NK-cell lymphomas. MR, Mendelian randomization; nsnp, number of single nucleotide polymorphism; OR, odds ratio; CI, confidence interval.

The IVW analysis indicated that eleven gut microbiota taxa had causal effects on mature T/NK-cell lymphomas, as illustrated in **Figure 4**. We found that the genus Ruminococcaceae UCG004 (OR: 2.06, 95% CI: 1.05–4.04, *p* = 0.035) was positively correlated with the risk of mature T/NK-cell lymphomas. Conversely, several taxa showed negative correlations with the risk, including the family Methanobacteriaceae (OR: 0.51, 95% CI: 0.32–0.84, *p* = 0.007) and the genus Methanobrevibacter (OR: 0.50, 95% CI: 0.27–0.92, *p* = 0.026), the family Lactobacillaceae (OR: 0.51, 95% CI: 0.28–0.94, *p* = 0.031) and the genus Lactobacillus (OR: 0.51, 95% CI: 0.28–0.91, *p* = 0.023), the family Verrucomicrobiaceae (OR: 0.44, 95% CI: 0.20–0.98, *p* = 0.044) and the genus Akkermansia (OR: 0.45, 95% CI: 0.20–0.98, *p* = 0.044), the genus Bifidobacterium (OR: 0.51, 95% CI: 0.26–0.99, *p* = 0.047), the genus Eubacterium oxidoreducens group (OR: 0.44, 95% CI: 0.21–0.92, *p* = 0.030), the genus Ruminococcaceae UCG014 (OR: 0.41, 95% CI: 0.28–0.96, *p* = 0.040) and the genus Lachnospiraceae UCG001 (OR: 0.38, 95% CI: 0.20–0.69, *p* = 0.002). Both the weighted median and weighted mode demonstrated consistent trends in ORs. Visual representations of the causal relationships between significant bacteria and mature T/NK-cell lymphomas are demonstrated in scatter plots (**Supplementary Figure S1**).

**Figure 5:**
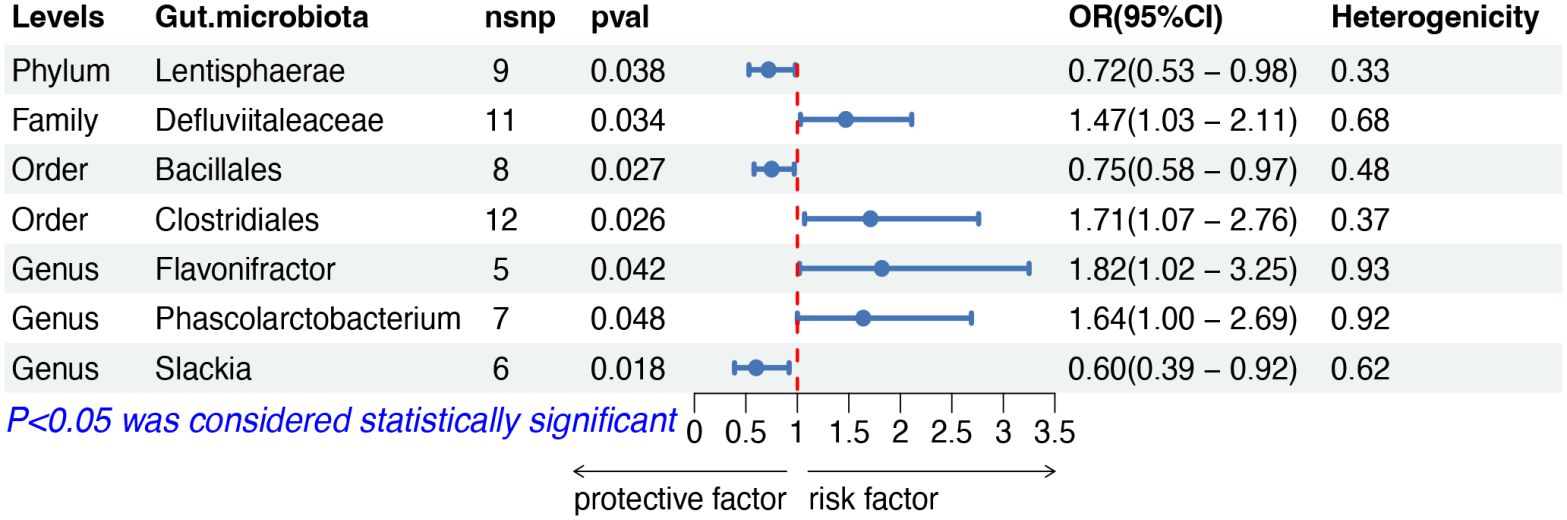
MR results of causal effects between gut microbiota and other and unspecified types of NHL. MR, Mendelian randomization; NHL, non-Hodgkin lymphoma; nsnp, number of single nucleotide polymorphism; OR, odds ratio; CI, confidence interval.

The IVW analysis indicated that seven gut microbiota taxa had causal effects on other and unspecified types of NHL, as illustrated in **Figure 5**. We found that the order Clostridiales (OR: 1.71, 95% CI: 1.07–2.76, *p* = 0.026), the family Defluviitaleaceae (OR: 1.47, 95% CI: 1.03–2.11, *p* = 0.034), the genus Flavonifractor (OR: 1.82, 95% CI: 1.02–3.25, *p* = 0.042) and the genus Phascolarctobacterium (OR: 1.64, 95% CI: 1.00–2.69, *p* = 0.048) showed a positive correlation with the risk of other and unspecified types of NHL. Conversely, the phylum Lentisphaerae (OR: 0.72, 95% CI: 0.53–0.98, *p* = 0.038), the order Bacillales (OR: 0.75, 95% CI: 0.58–0.97, risk of lymphoma, while the phylum *p* = 0.027), and the genus Slackia (OR: 0.60, 95% CI: 0.39–0.92, *p* = 0.018) were negatively correlated with the risk. Both the weighted median and weighted mode demonstrated consistent trends in ORs. Visual representations of the causal relationships between significant bacteria and other and unspecified types of NHL are demonstrated in scatter plots (**Supplementary Figure S1**).

**Figure 6:**
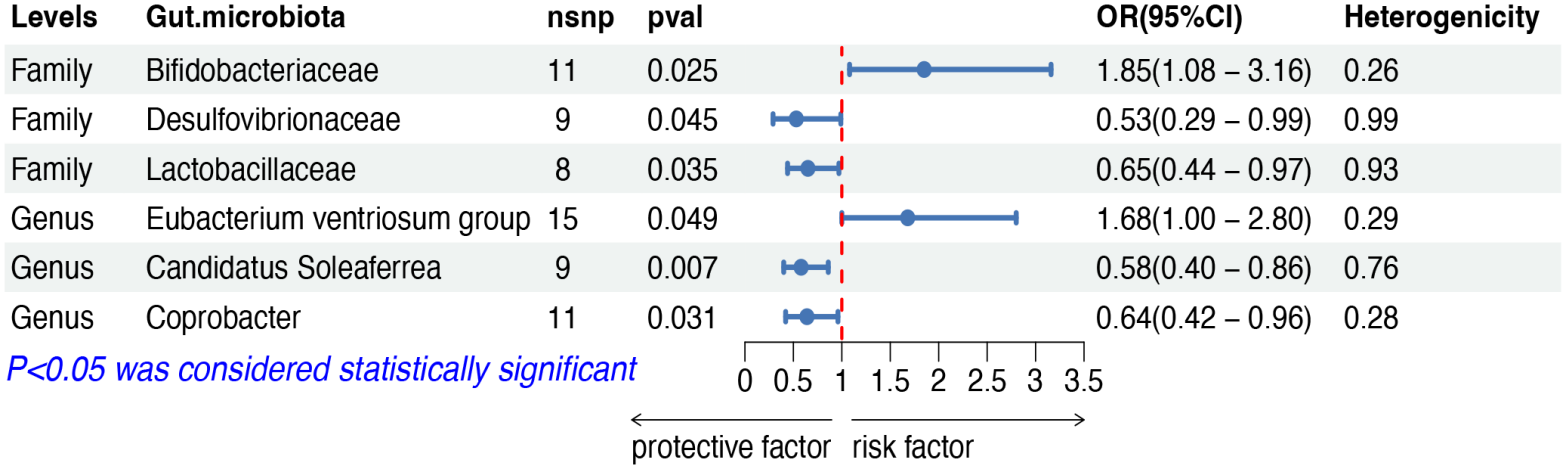
MR results of causal effects between gut microbiota and HL. MR, Mendelian randomization; HL, Hodgkin lymphoma; nsnp, number of single nucleotide polymorphism; OR, odds ratio; CI, confidence interval.

The IVW analysis indicated that six gut microbiota taxa had causal effects on HL, as illustrated in **Figure 6**. We found that the family Bifidobacteriaceae (OR: 1.85, 95% CI: 1.08–3.16, *p* = 0.025) and the genus Eubacterium ventriosum group (OR: 1.68, 95% CI: 1.00–2.80, *p* = 0.049) were positively correlated with the risk of HL, while the family Desulfovibrionaceae (OR: 0.53, 95% CI: 0.29–0.99, *p* = 0.045), the family Lactobacillaceae (OR: 0.65, 95% CI: 0.44–0.97, *p* = 0.035), the genus Candidatus Soleaferrea (OR: 0.58, 95% CI: 0.40–0.86, *p* = 0.007) and the genus Coprobacter (OR: 0.63, 95% CI: 0.42–0.96, *p* = 0.031) showed a negative correlation with HL risk. Both the weighted median and weighted mode demonstrated consistent trends in ORs. Visual representations of the causal relationships between significant bacteria and HL are demonstrated in scatter plots (**Supplementary Figure S1**).

In the sensitivity analyses, we assessed horizontal pleiotropy and heterogeneity, as detailed in **Supplementary Table S12**-**S13** and **Supplementary Figure S2**. The MR-PRESSO test revealed no signs of horizontal pleiotropy among these SNPs, as indicated by a *p*-value exceeding 0.05 in the global test. Additionally, according to Cochran’s Q statistics, we detected no pleiotropy due to cross-instrument effects, with the Cochran’s Q for IVW being exceeding 0.05. The MR-Egger analysis confirmed the absence of directional pleiotropy, with its p-value intercept also being greater than 0.05. Moreover, the leave-one-out analysis demonstrated the robustness and stability of the results. The sensitivity analyses of gut microbiota, which demonstrated significant causal relationships with lymphoma subtypes, are presented in **Table 1**.

**Table 1:** Sensitivity analysis of the causal association between gut microbiota and lymphoma.

Subtype	Microbiota	Cochran Q test	MR-PRESSO	MR-Egger	
		*p*_value	*p*_value	Intercept	*p*_value
Diffuse large B-cell lymphoma	Genus Ruminococcaceae UCG002	0.941	0.970	-0.012	0.731
	Genus Coprobacter	0.413	0.450	-0.031	0.686
	Genus Alistipes	0.854	0.870	-0.110	0.197
	Genus Turicibacter	0.241	0.299	-0.016	0.889
Follicular lymphoma	Order Bacillales	0.362	0.398	0.047	0.612
	Family Bacteroidales S24 7group	0.673	0.696	-0.016	0.847
	Family XIII	0.382	0.415	0.014	0.885
	Family Peptostreptococcaceae	0.678	0.689	0.036	0.334
	Genus Eubacterium ventriosum group	0.946	0.946	0.029	0.677
	Genus Ruminiclostridium9	0.703	0.727	0.068	0.495
	Genus Haemophilus	0.472	0.467	-0.011	0.823
	Genus Ruminococcaceae NK4A214 group	0.105	0.131	-0.030	0.659
Mature T/NK-cell lymphomas	Family Verrucomicrobiaceae	0.310	0.312	0.108	0.334
	Family Methanobacteriaceae	0.943	0.955	-0.011	0.945
	Family Lactobacillaceae	0.832	0.806	0.010	0.922
	Genus Bifidobacterium	0.818	0.828	-0.069	0.320
	Genus Lactobacillus	0.903	0.891	0.009	0.923
	Genus Methanobrevibacter	0.773	0.796	0.079	0.455
	Genus Akkermansia	0.309	0.322	0.107	0.340
	Genus Eubacterium oxidoreducens group	0.606	0.651	-0.187	0.304
	Genus Ruminococcaceae UCG014	0.280	0.372	0.063	0.489
	Genus Lachnospiraceae UCG001	0.477	0.521	-0.050	0.696
	Genus Ruminococcaceae UCG004	0.824	0.834	0.080	0.632
Other and unspecified types of non-Hodgkin lymphoma	Phylum Lentisphaerae	0.330	0.378	-0.065	0.492
	Family Defluviitaleaceae	0.844	0.691	0.073	0.282
	Order Bacillales	0.478	0.538	-0.011	0.899
	Order Clostridiales	0.366	0.419	0.045	0.348
	Genus Flavonifractor	0.927	0.933	-0.056	0.606
	Genus Phascolarctobacterium	0.924	0.934	0.101	0.373
	Genus Slackia	0.624	0.704	-0.189	0.251
Hodgkin lymphoma	Family Bifidobacteriaceae	0.262	0.322	0.065	0.337
	Family Desulfovibrionaceae	0.994	0.995	0.004	0.963
	Family Lactobacillaceae	0.928	0.928	0.002	0.973
	Genus Eubacterium ventriosum group	0.288	0.285	0.060	0.511
	Genus Candidatus Soleaferrea	0.759	0.772	0.008	0.966
	Genus Coprobacter	0.282	0.311	0.020	0.833

#### The causal effects of lymphoma on gut microbiota

We performed reverse MR analyses to explore potential causal associations between lymphoma subtypes and forward significant bacteria. Considering the limited identification of lymphoma associated SNPs identified employing the strict threshold at *p* < 5 × 10^-8^, we adopted a more lenient threshold to mitigate potential inaccuracies arising from an insufficient number of SNPs. Except for a reverse causal relationship between the other and unspecified types of NHL and the phylum Lentisphaerae, which was excluded to guarantee the robustness of our results, we generally found no statistically significant associations using the IVW method, as detailed in **Supplementary Table S14**-**S16**.

## Discussion

To our knowledge, this study is believed to be the first MR analysis to investigate the possible causal link between gut microbiota and lymphoma, representing a pre-lymphoma longitudinal study of the microbiota. We investigated the potential involvement of 196 distinct microbial taxa in the etiology of malignant lymphoma, utilizing the most comprehensive microbiome GWAS summary data available. Our results indicate causal associations between changes in the abundance of certain microbial groups and the development of lymphoma.

Emerging studies suggested that gut microbiota could regulate the formation of lymphoma through various mechanisms, including aberrant activation of the immune system, generation of both pro-inflammatory and anti-inflammatory responses, and modulation of metabolic processes (Shi and Zhang, 2021). In our study, the genus Phascolarctobacterium was associated with an increased risk of lymphoma, while the phylum Lentisphaerae, the family Desulfovibrionaceae and the genus Haemophilus within the phylum Proteobacteria, along with the family Methanobrevibacter and the genus Methanobrevibacter within the phylum Euryarchaeotaare, were found to be protective factors against lymphoma. Interestingly, the microbiota within the phylum Firmicutes, Bacteroidetes, Actinobacteria and Verrucomicrobia can act as either risk or protective factors depending on the lymphoma subtypes. This phenomenon may be attributed to distinct pathogenic mechanisms among different tumor subtypes, heterogeneous immune responses of tumor cells to microorganisms across subtypes, and the influence of the tumor microenvironment, necessitating further validation through animal experiments and clinical trials.

In this study, the order Clostridiales within the class Clostridia was identified as a risk factor for lymphoma, although its suborder’s microbiota partly promotes tumorigenesis and partly inhibits tumor formation. Research studies have shown that the class Clostridia promotes the differentiation of CD4^+^Foxp3^+^Tregs cells, which subsequently induce the production of IgA^+^ B-cells in the intestinal tract (Atarashi et al., 2011). These B-cells can decrease the absorption of antigens derived from the microbiota in mucosal tissues and reduce the activation of systemic T-cell activation (Cong et al., 2009). Meanwhile, butyric acid, a metabolite of the class Clostridia (Vital et al., 2014), helps inhibit the activation of the NF-κB signaling pathway (Inan et al., 2000). These mechanisms collectively contribute to maintaining immune homeostasis, suppressing deleterious inflammation, and thereby inhibiting tumor formation. Importantly, inflammatory lymphomas are characterized by significant immune cell infiltration, particularly of T cells, frequent mutations that lead to persistent activation of the NF-κB pathway, and a heightened sensitivity to immune checkpoint blockade therapy (Kline et al., 2020). Consistent with these studies, family Peptostreptococcaceae, genus Eubacterium oxidoreducens group, Ruminococcaceae UCG014 and Ruminococcaceae NK4A214 group, Lachnospiraceae UCG001, Slackia, and Turicibacter were found to inhibit tumorigenesis.

Recently, numerous studies have focused on investigating the correlation between gut microbiota along with its metabolites and lymphoma. Gut microbiota, often referred to as the “new virtual metabolic organ”, regulates various metabolic pathways in the host (Evans et al., 2013). Some microbial metabolites can promote or inhibit carcinogenesis. For instance, most short-chain fatty acids (SCFAs), produced by the fermentation of dietary fibers by the two main phyla, Firmicutes and Bacteroidetes, are considered to have anticancer effects. SCFAs act as ligands for G protein-coupled receptors found throughout the gastrointestinal tract and on immune cells, and have been implicated in regulating inflammation and cancer progression (Zhang and Davies, 2016). Additionally, butyrate, a histone deacetylase inhibitor, initiates apoptosis and prevents tumor cell proliferation through the Warburg effect, enhancing histone 3 acetylation and the expression of target genes such as Fas, P21, P27, etc (Vander Heiden et al., 2009; Wei et al., 2016). Lu et al. found that a decrease in Fusobacterium rectum led to butyrate deficiency in patients with lymphoma, failed to inhibit lymphomagenesis by suppressing the TNF-induced TLR4/MyD88/NF-κB axis (Lu et al., 2022). Interestingly, butyrate can also promote tumor formation by facilitating the extra-thymic production of Treg cells (Arpaia et al., 2013). Consistent with previous observational and animal studies, our study showed that group Eubacterium oxidoreducens, Ruminococcaceae UCG014, Ruminococcaceae NK4A214 group, and Lachnospiraceae UCG001 within the phylum Firmicutes, and the genus Alistipes and Coprobacter within the phylum Bacteroidetes function as protective factors for lymphoma. We also found genus Eubacterium ventriosum Group, Flavonifractor, Ruminococcaceae UCG002, Ruminococcaceae UCG004, and Ruminiclostridium 9 within the phylum Firmicutes to be risk factors for different types of lymphoma, suggesting that different genera of family Eubacteriaceae, Ruminococcaceae, and Lachnospiraceae may have distinct mechanisms of action in different lymphoma types.

Akkermansia muciniphila, a representative species of the phylum Verrucomicrobia in the human intestine, along with genus Lactobacillus and genus Alistipes, are considered important probiotic microorganisms in the human gut (Cani et al., 2022). These probiotics are thought to enhance antitumor activity by improving host metabolism, modulating the immune response and increasing efficacy of immune checkpoint inhibitors in patients with FL (Routy et al., 2018; Merryman et al., 2023). Certain strains of genus Lactobacillus and Bifidobacterium can inhibit the growth of Helicobacter pylori by releasing bacteriocins or organic acids, and may reduce its attachment to gastric epithelial cells, thereby lowering the risk of gastric adenocarcinoma and lymphoma (Gotteland et al., 2006). Furthermore, castalagin, which is enriched in bacteria associated with effective immunotherapeutic responses (e.g., family Ruminococcaceae and genus Alistipes), improves the ratio of CD8^+^ cells to FOXP3^+^CD4^+^ cells in the tumor microenvironment (Messaoudene et al., 2022).

Above all, our research contributes new perspectives on the potential causality between gut microbiota and lymphoma, which have not previously been reported. One major strength of this study lies in its utilization of a MR approach, which helps minimize confounding factors and biases commonly observed in observational studies, thereby enhancing the credibility of the results. Although the MR approach offers several benefits over traditional epidemiological research, interpreting the results requires considerable caution. This caution is necessary due to potential variability in methodologies used across different cohorts within the MiBioGen consortium, as well as the dynamic and complex nature of the gut microbiota within its ecosystem. Consequently, further epidemiological studies and clinical trials are essential to more definitively determine the causal relationship between gut microbiota and lymphoma. Additionally, the resolution at the genus level provided by 16S sequencing is limited; therefore, alternative approaches, such as shotgun metagenomics, metatranscriptomics, proteomic analysis, and metabolomic profiling are recommended. These methods will enable better harmonization of GWAS data and lead to a more comprehensive understanding of the microbiome’s involvement in lymphoma.

In conclusion, our study provides evidence for potential associations between alterations in the composition of gut microbiota and different subtypes of lymphoma. We discovered that several microbial taxa have causal effects on lymphoma, offering valuable insights into prophylactic and therapeutic targets against lymphoma. These findings suggest that microbial prophylaxis or interventions such as probiotic administration, fecal microbiota transplantation, or dietary modifications warrant further exploration.

## Data availability statement

The original contributions presented in the study are included in the article/**Supplementary Material**. Further inquiries can be directed to the corresponding author.

## Ethics statement

Ethical approval was not required for the study involving humans in accordance with the local legislation and institutional requirements. Written informed consent to participate in this study was not required from the participants or the participants’ legal guardians/next of kin in accordance with the national legislation and the institutional requirements.

## Author contributions

JL: Conceptualization, Data curation, Formal Analysis, Investigation, Methodology, Project administration, Software, Supervision, Validation, Writing – original draft, Writing – review & editing. GL: Conceptualization, Data curation, Methodology, Software, Supervision, Writing – original draft, Writing – review & editing. WW: Data curation, Formal Analysis, Project administration, Writing – review & editing. HX: Formal Analysis, Funding acquisition, Project administration, Resources, Supervision, Validation, Visualization, Writing – review & editing.
