# Supplementary material for: Causal relationships between gut microbiota and lymphoma: a bidirectional Mendelian randomization study
Source: Front Cell Infect Microbiol. 2024 May 13;14:1374775. doi: 10.3389/fcimb.2024.1374775 (PMC11128559; doi:10.3389/fcimb.2024.1374775)
Supplement: Supplementary file 1 [file Image_1.pdf]

Supplementary Figure S1. Scatter plots for the causal association between gut microbiota and lymphoma.

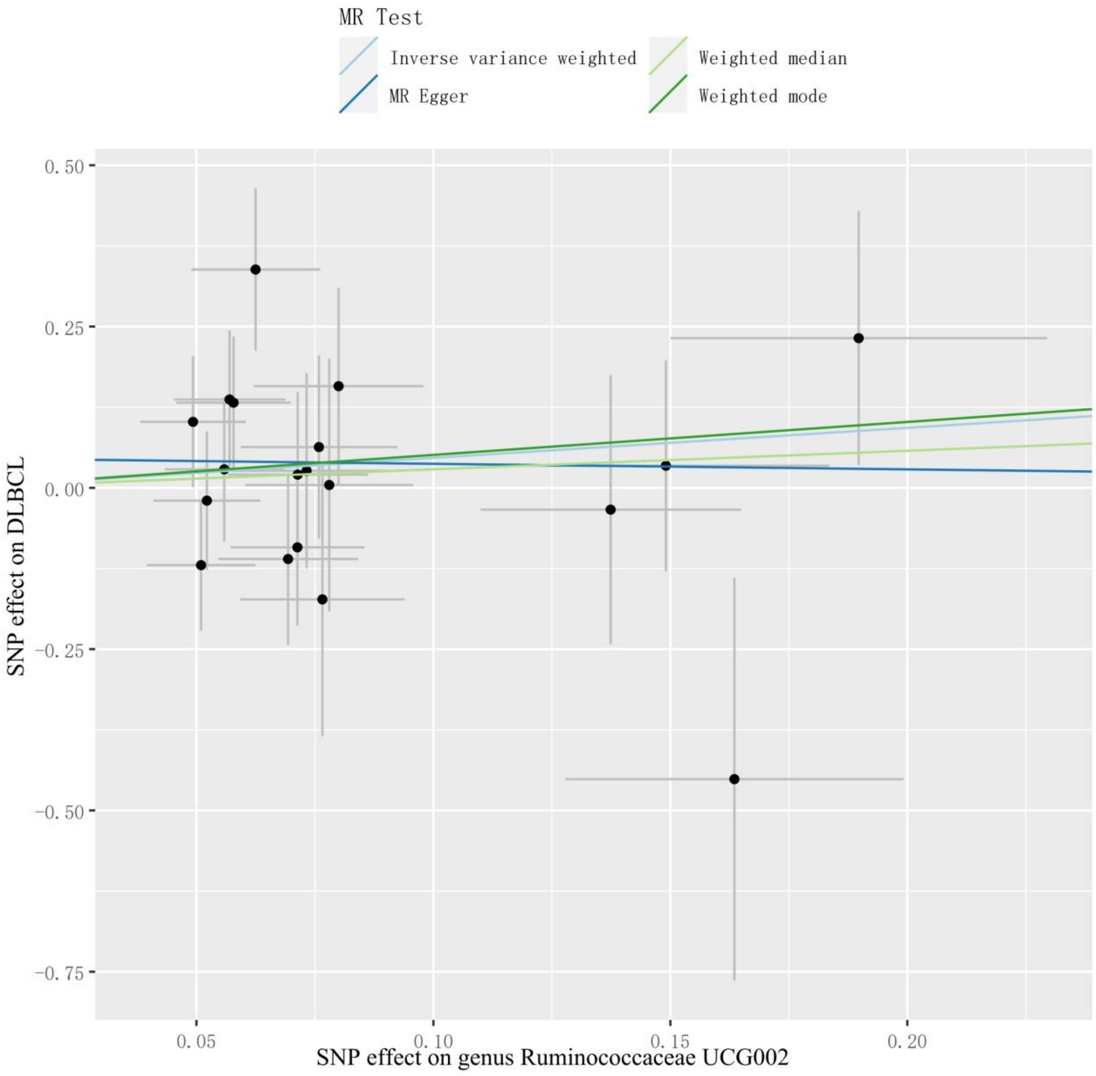

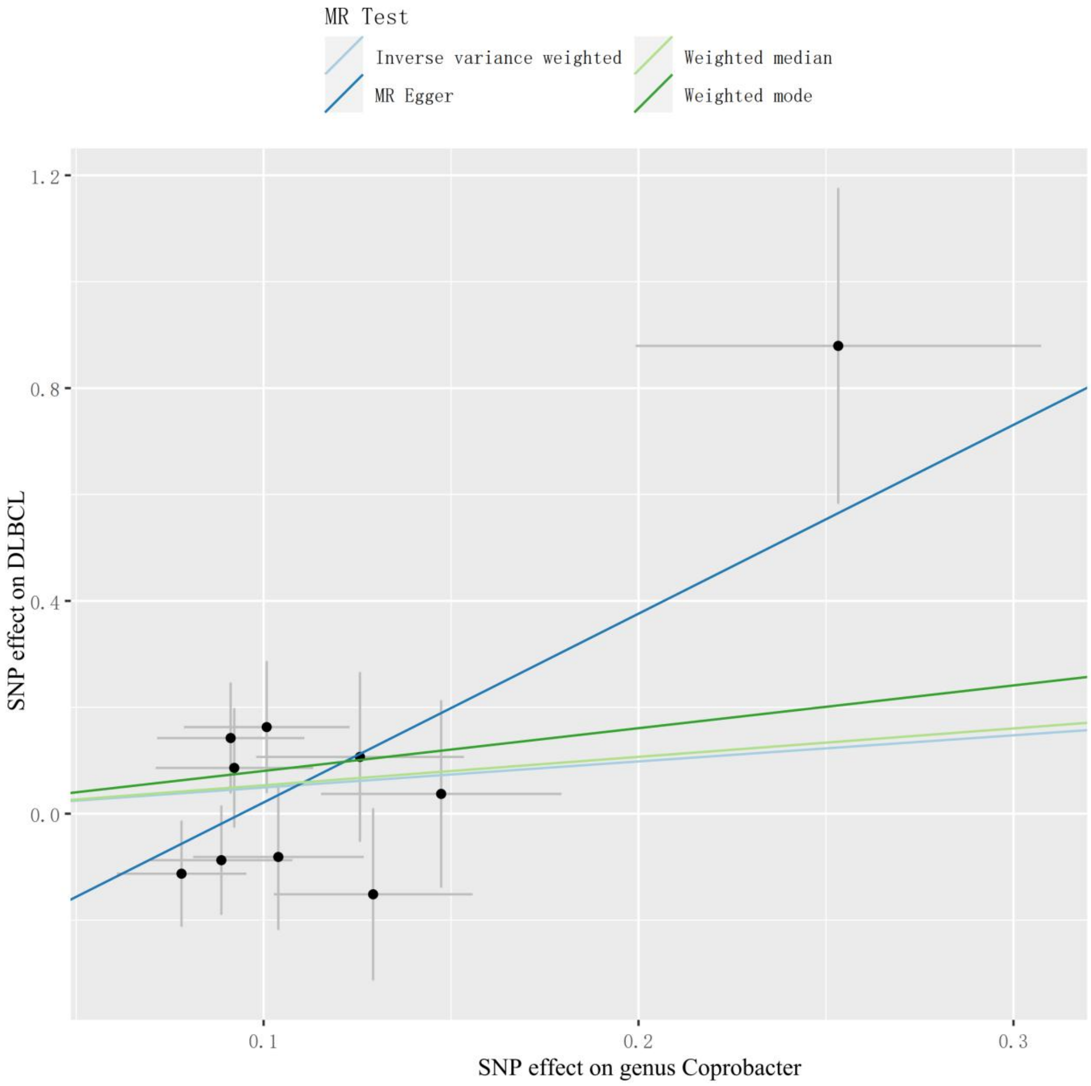
MR Test
Inverse variance weighted
MR Egger
Weighted median
Weighted mode
1.2
0.8
0.4
0.0
0.1
0.2
0.3
SNP effect on DLBCL
SNP effect on genus Coprobacter

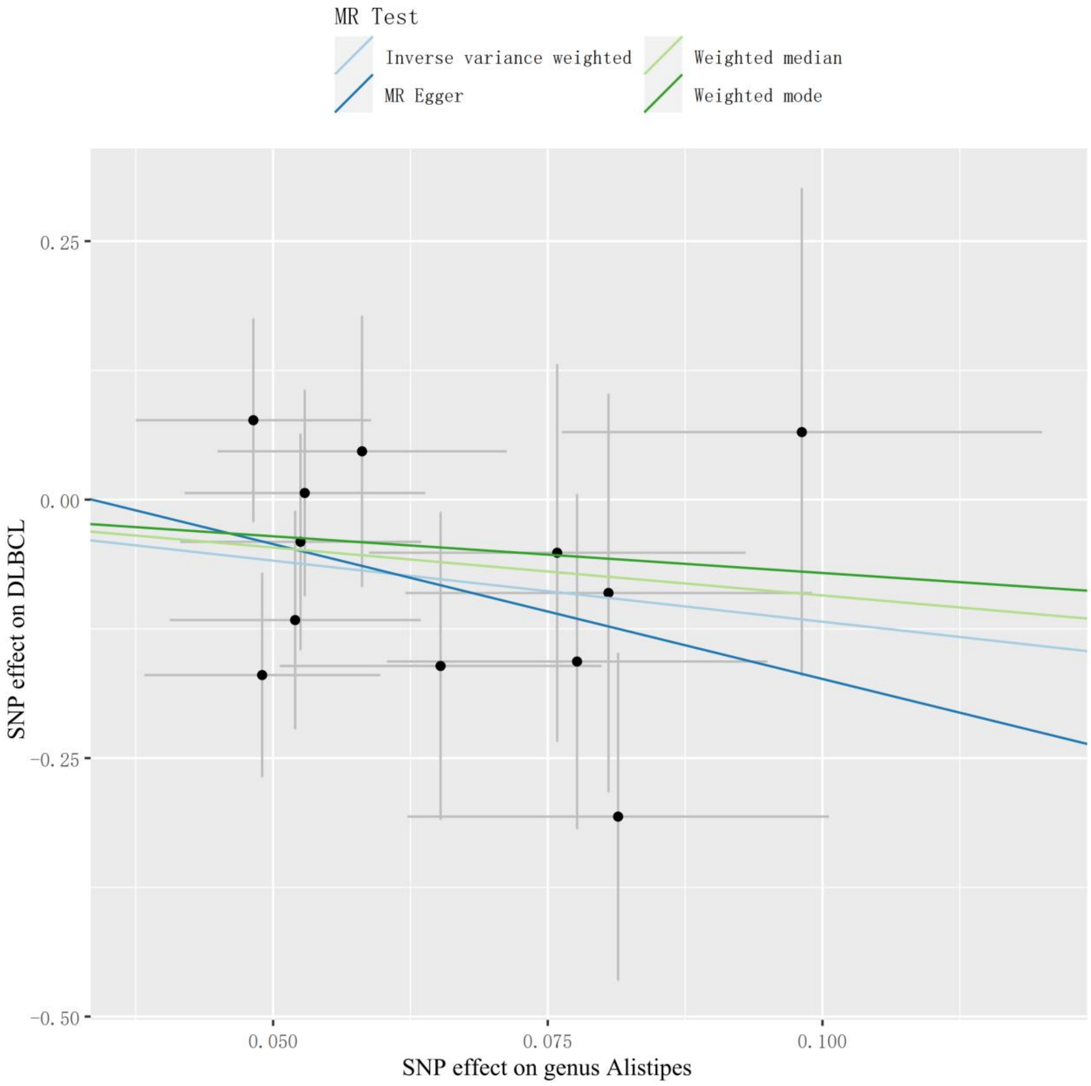
MR Test
Inverse variance weighted
MR Egger
Weighted median
Weighted mode
0.25
0.00
−0.25
−0.50
0.050
0.075
0.100
SNP effect on DLBCL
SNP effect on genus Alistipes

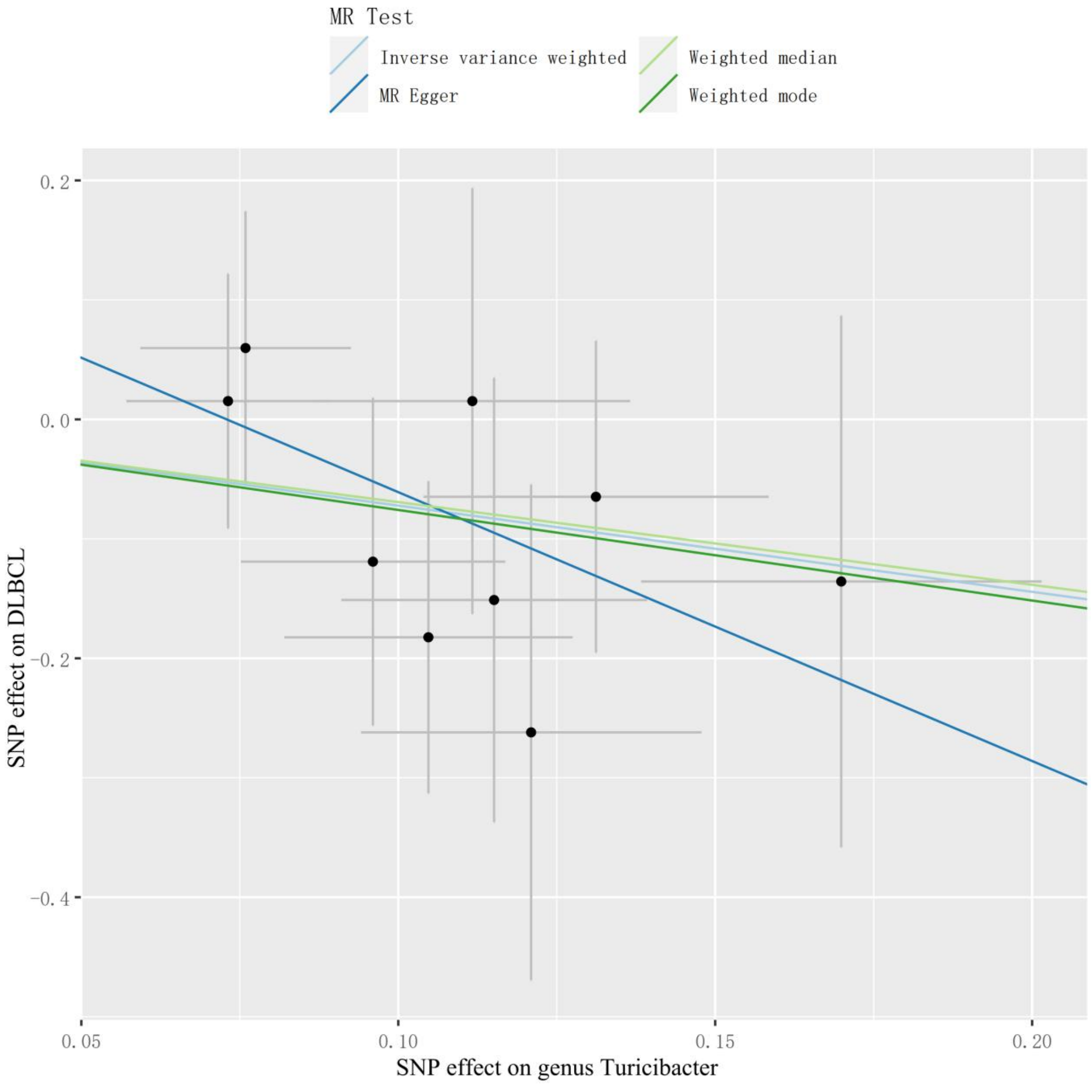
MR Test
Inverse variance weighted
MR Egger
Weighted median
Weighted mode
0.2
0.0
−0.2
−0.4
0.05
0.10
0.15
0.20
SNP effect on DLBCL
SNP effect on genus Turicibacter

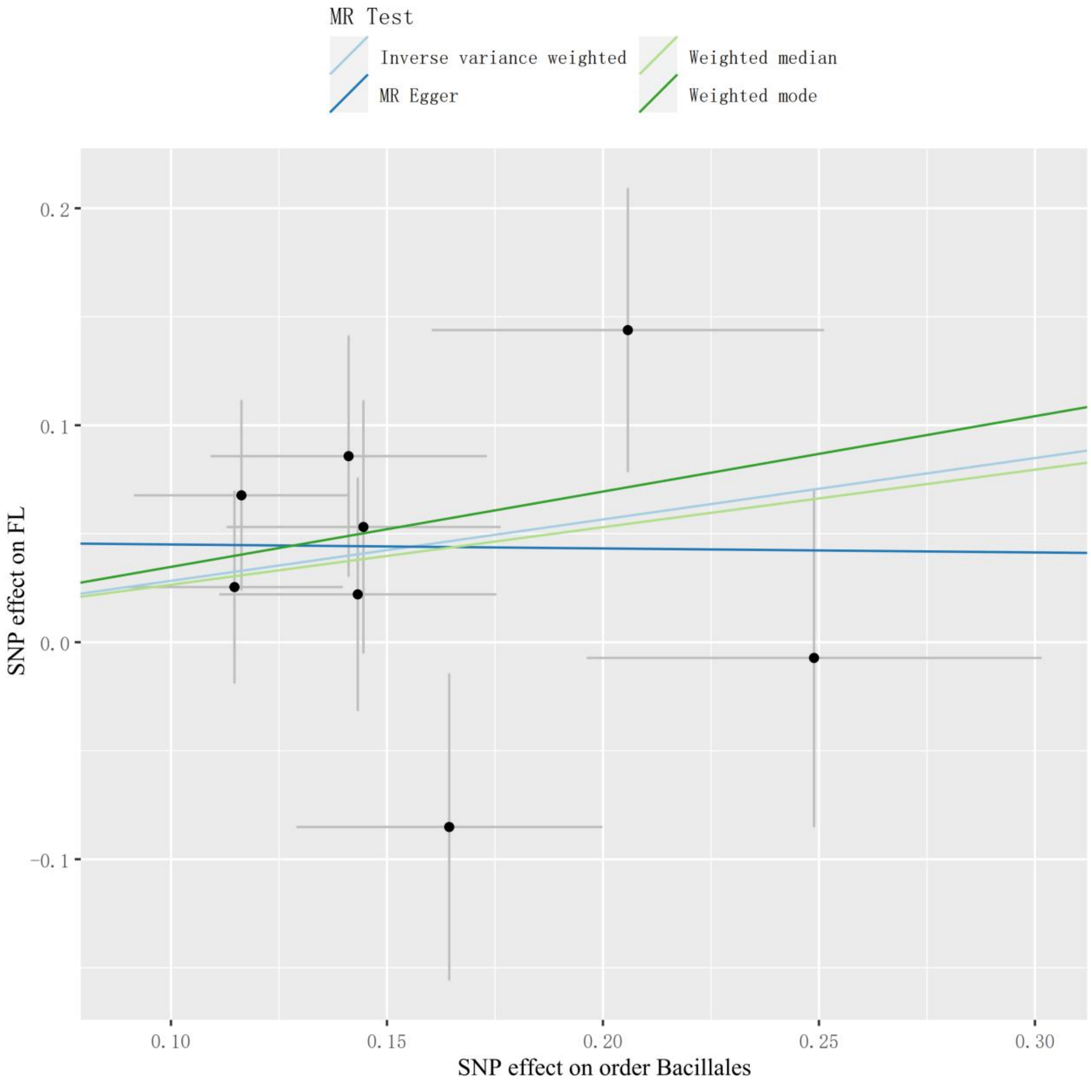
MR Test
Inverse variance weighted
Weighted median
MR Egger
Weighted mode
0.2
0.1
0.0
-0.1
SNP effect on FL
0.10
0.15
0.20
0.25
0.30
SNP effect on order Bacillales

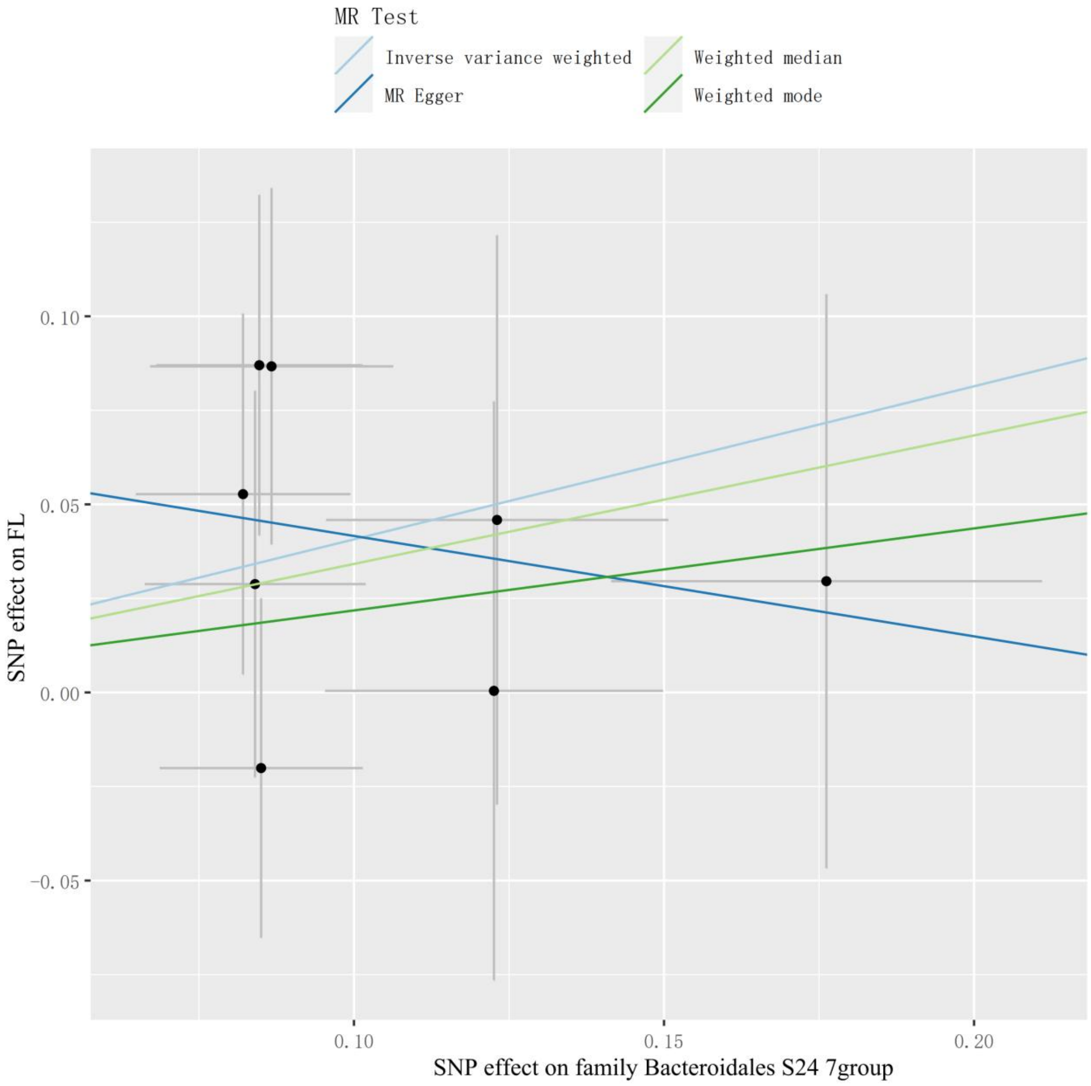
MR Test
Inverse variance weighted
MR Egger
Weighted median
Weighted mode
SNP effect on FL
0.10
0.05
0.00
−0.05
0.10
0.15
0.20
SNP effect on family Bacteroidales S24 7group

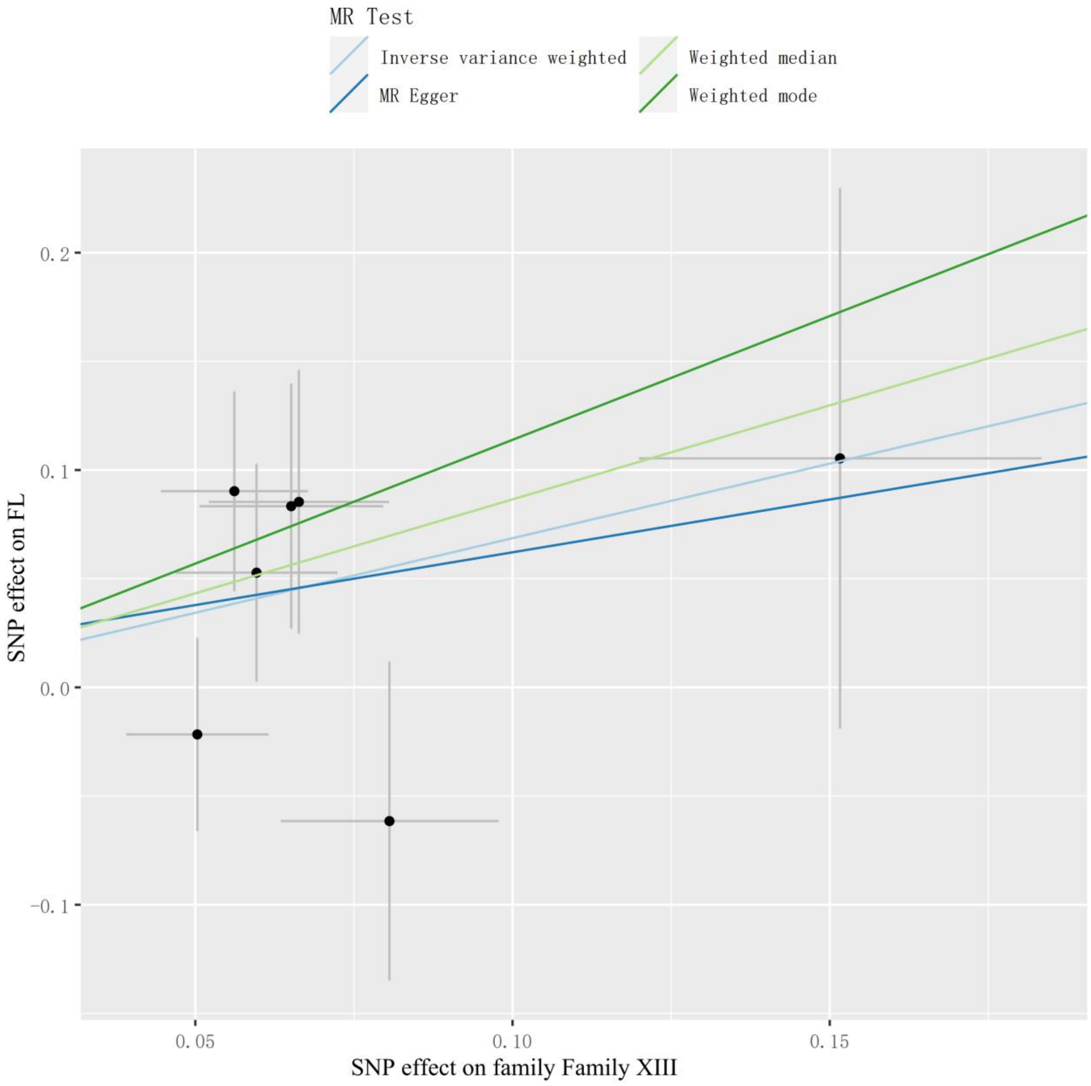

MR Test
Inverse variance weighted
MR Egger
Weighted median
Weighted mode
0.2
0.1
0.0
-0.1
SNP effect on FL
0.05
0.10
0.15
SNP effect on family Family XIII

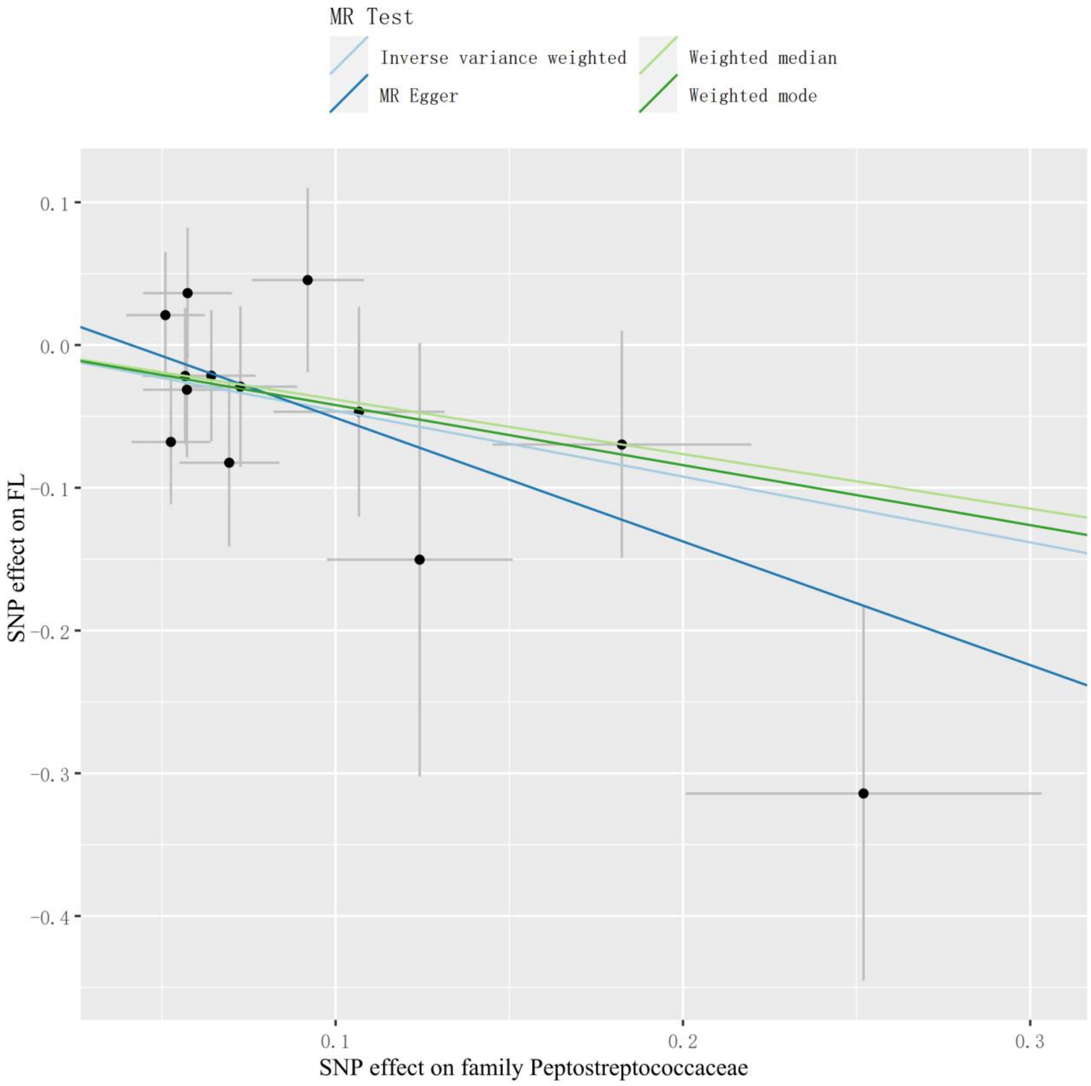
MR Test
Inverse variance weighted
MR Egger
Weighted median
Weighted mode
0.1
0.0
-0.1
-0.2
-0.3
-0.4
SNP effect on FL
0.1
0.2
0.3
SNP effect on family Peptostreptococcaceae

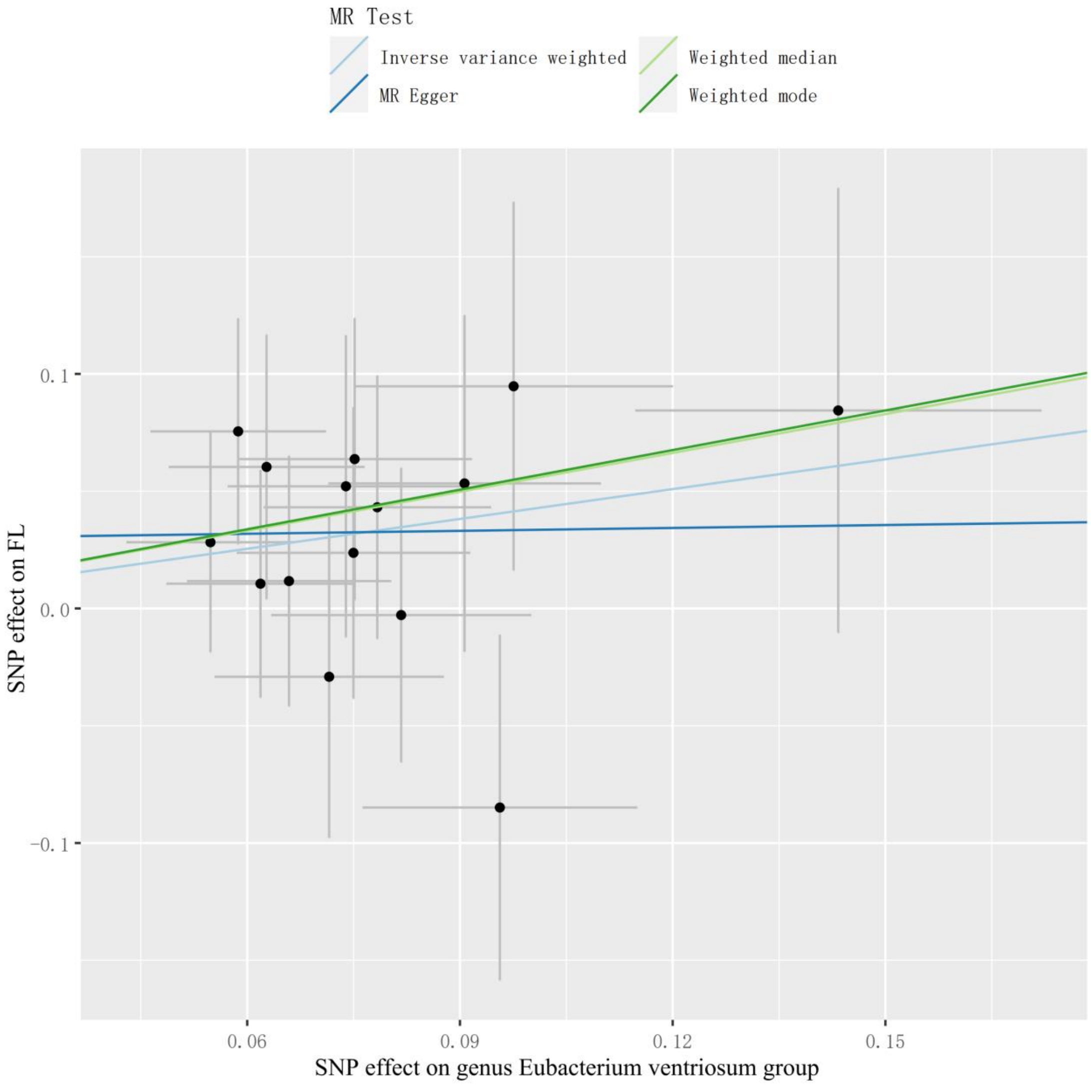
MR Test
Inverse variance weighted
MR Egger
Weighted median
Weighted mode
0.1
0.0
−0.1
SNP effect on FL
0.06
0.09
0.12
0.15
SNP effect on genus Eubacterium ventriosum group

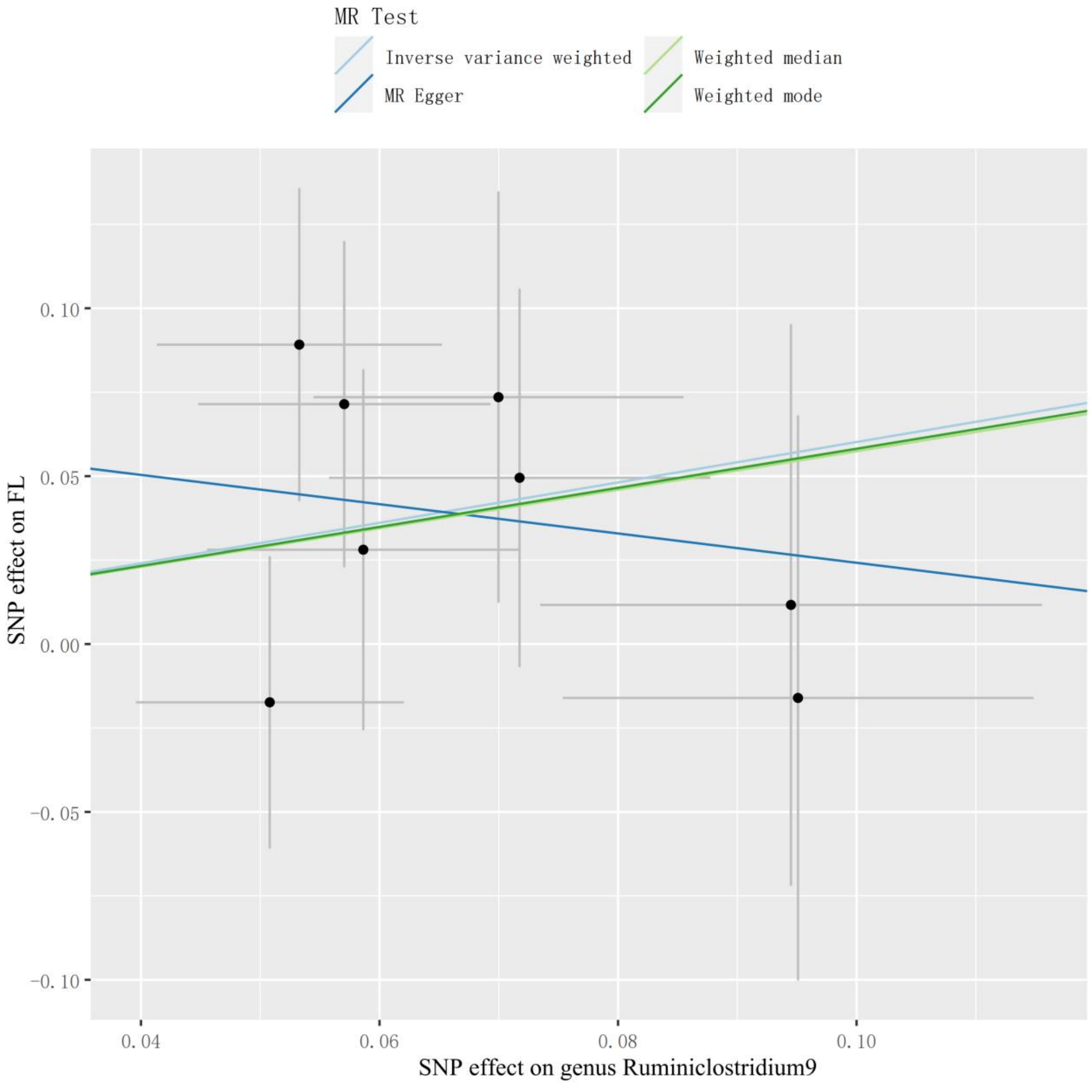

MR Test
Inverse variance weighted
MR Egger
Weighted median
Weighted mode
0.10
0.05
0.00
−0.05
−0.10
SNP effect on FL
0.04
0.06
0.08
0.10
SNP effect on genus Ruminiclostridium9

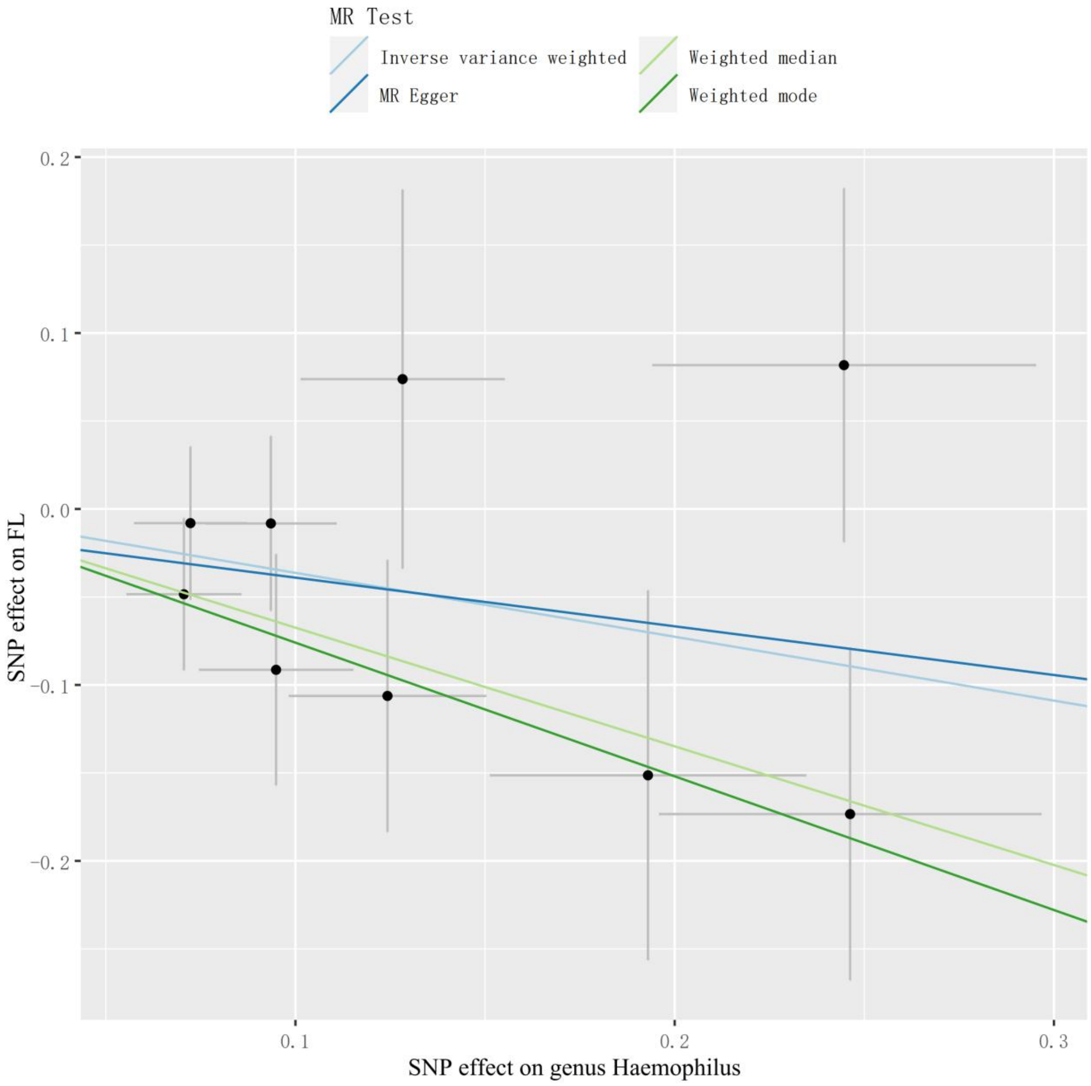

MR Test
Inverse variance weighted
MR Egger
Weighted median
Weighted mode
0.2
0.1
0.0
−0.1
−0.2
SNP effect on FL
0.1
0.2
0.3
SNP effect on genus Haemophilus

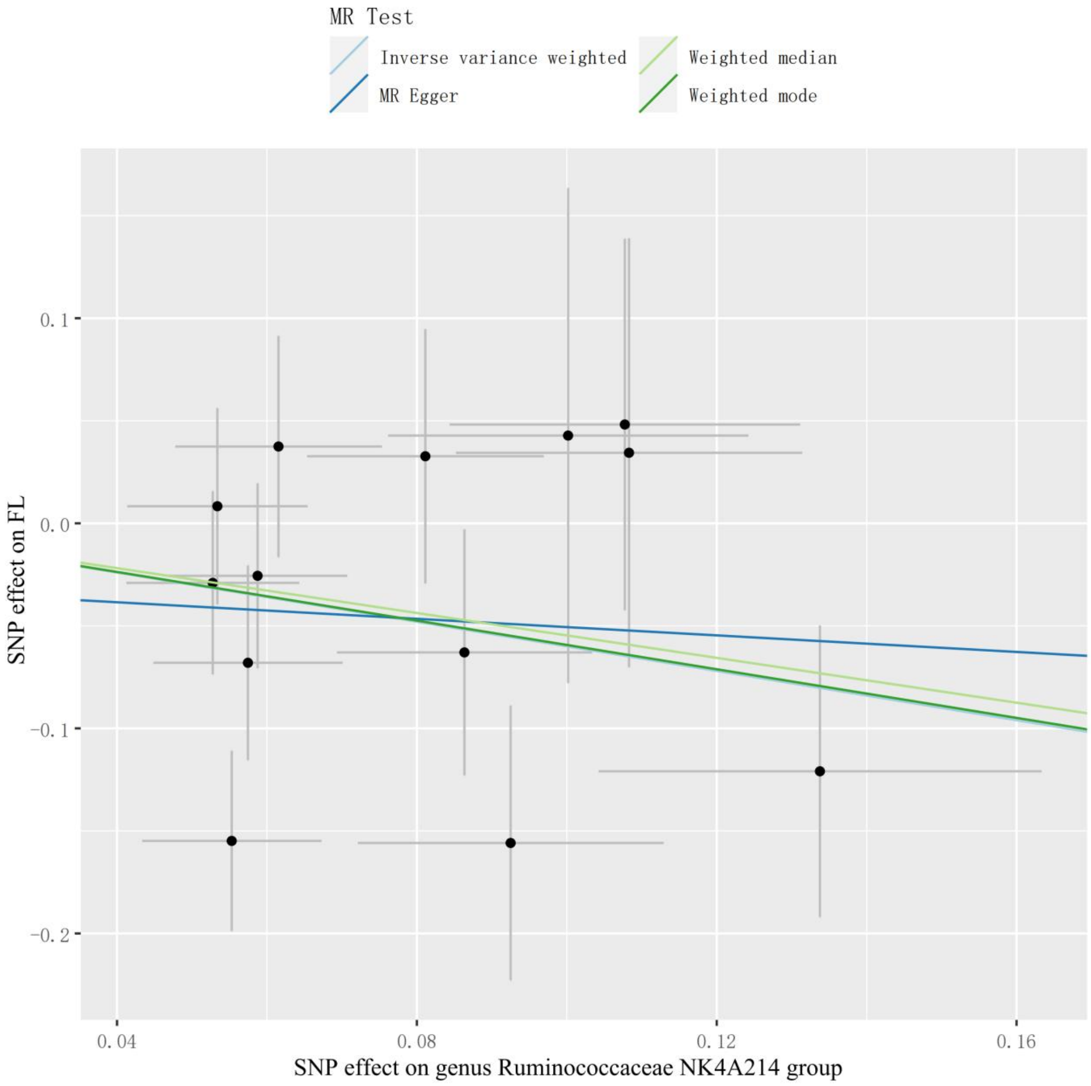
MR Test
Inverse variance weighted
MR Egger
Weighted median
Weighted mode
0.1
0.0
-0.1
-0.2
SNP effect on FL
0.04
0.08
0.12
0.16
SNP effect on genus Ruminococcaceae NK4A214 group

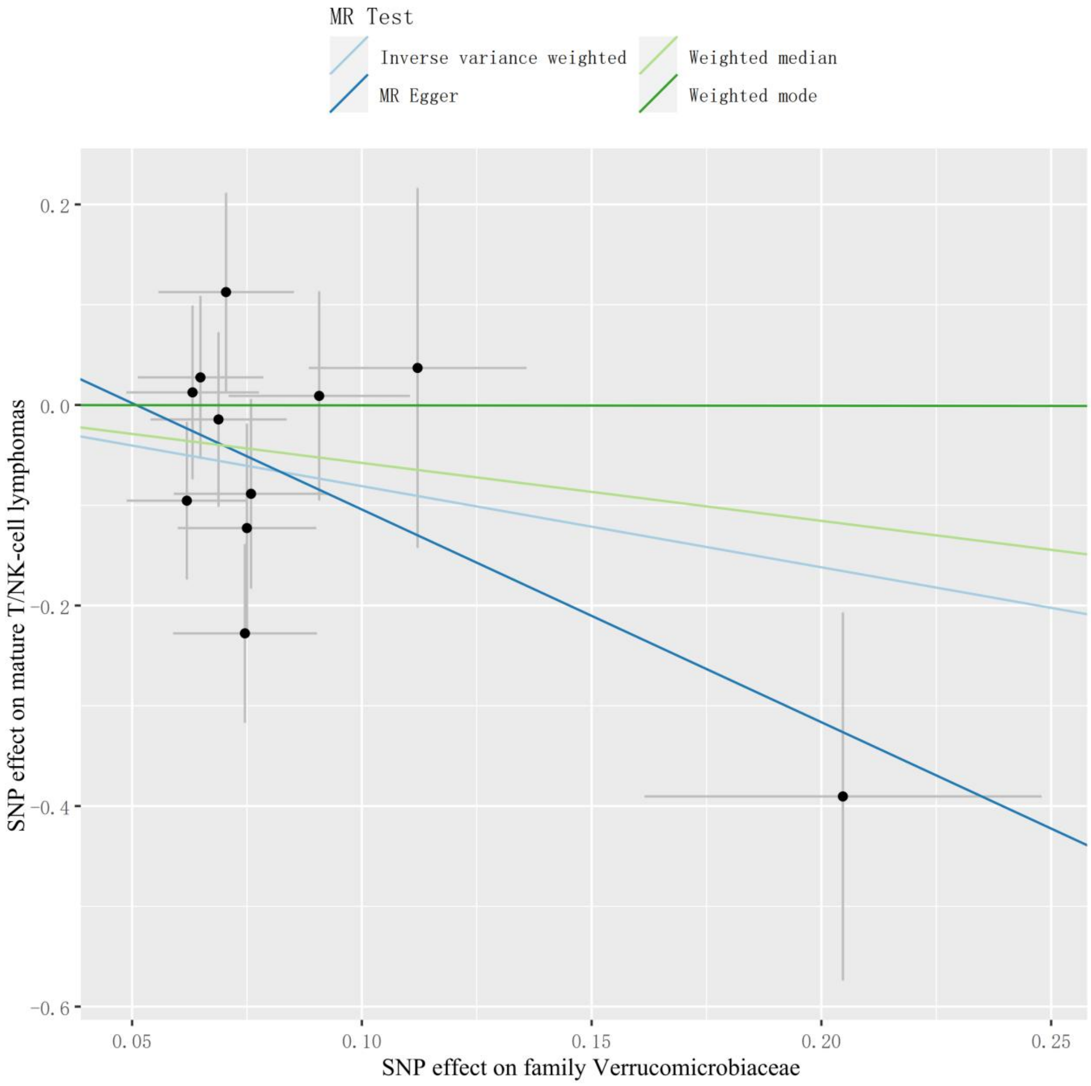
MR Test
Inverse variance weighted
MR Egger
Weighted median
Weighted mode
0.2
0.0
−0.2
−0.4
−0.6
0.05
0.10
0.15
0.20
0.25
SNP effect on mature T/NK-cell lymphomas
SNP effect on family Verrucomicrobiaceae

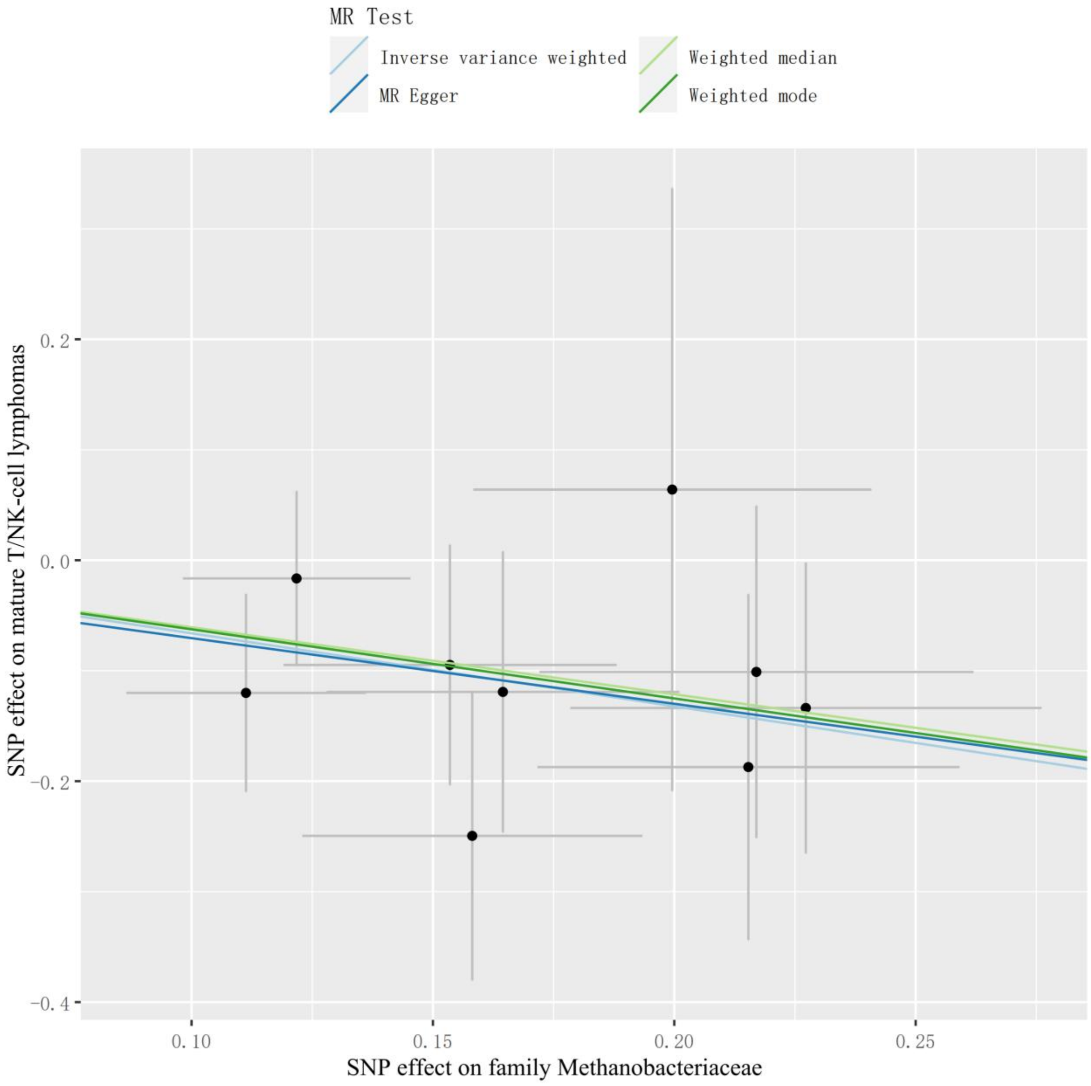

MR Test
Inverse variance weighted
MR Egger
Weighted median
Weighted mode
SNP effect on mature T/NK-cell lymphomas
0.2
0.0
−0.2
−0.4
0.10
0.15
0.20
0.25
SNP effect on family Methanobacteriaceae

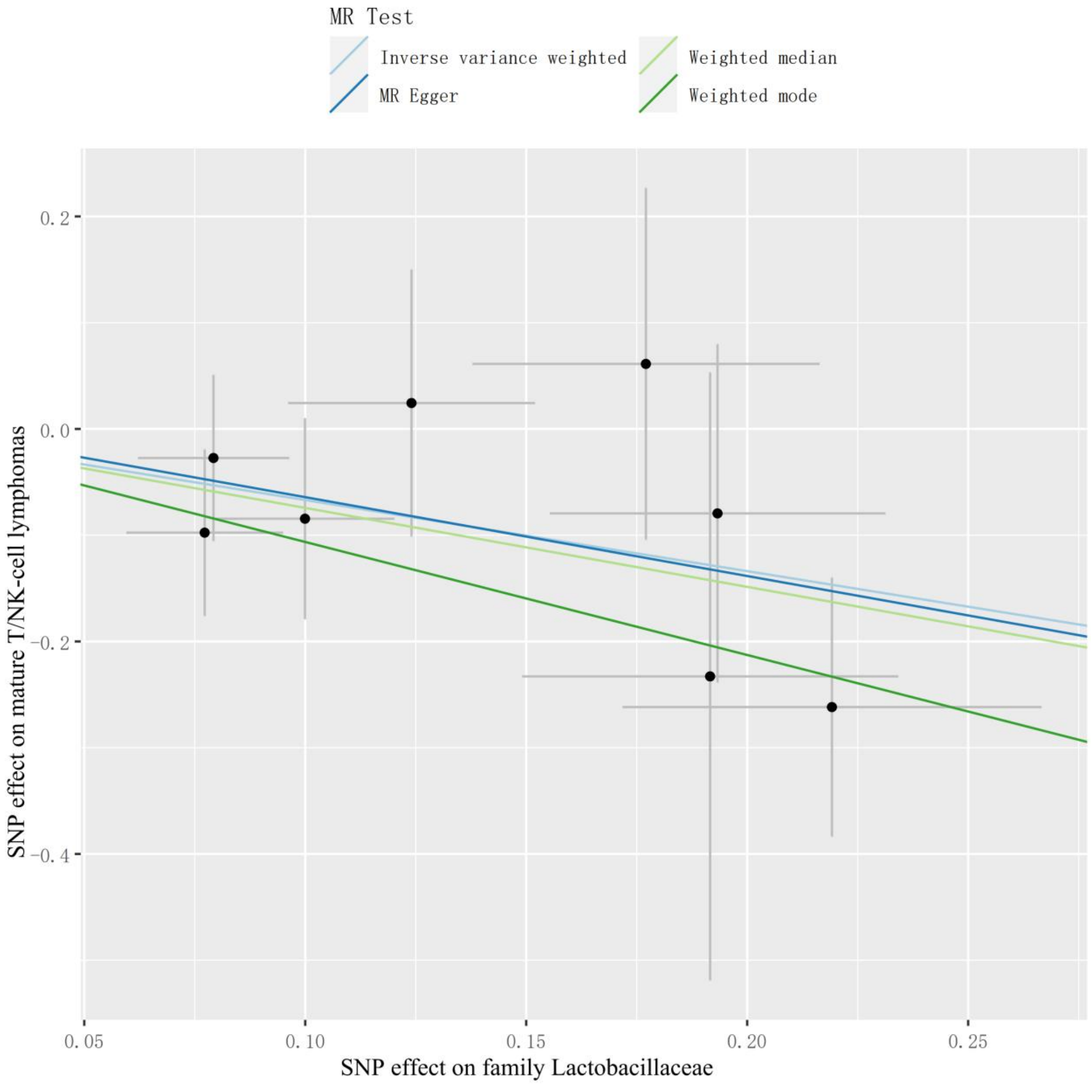

MR Test
Inverse variance weighted
MR Egger
Weighted median
Weighted mode
0.2
0.0
−0.2
−0.4
SNP effect on mature T/NK-cell lymphomas
0.05
0.10
0.15
0.20
0.25
SNP effect on family Lactobacillaceae

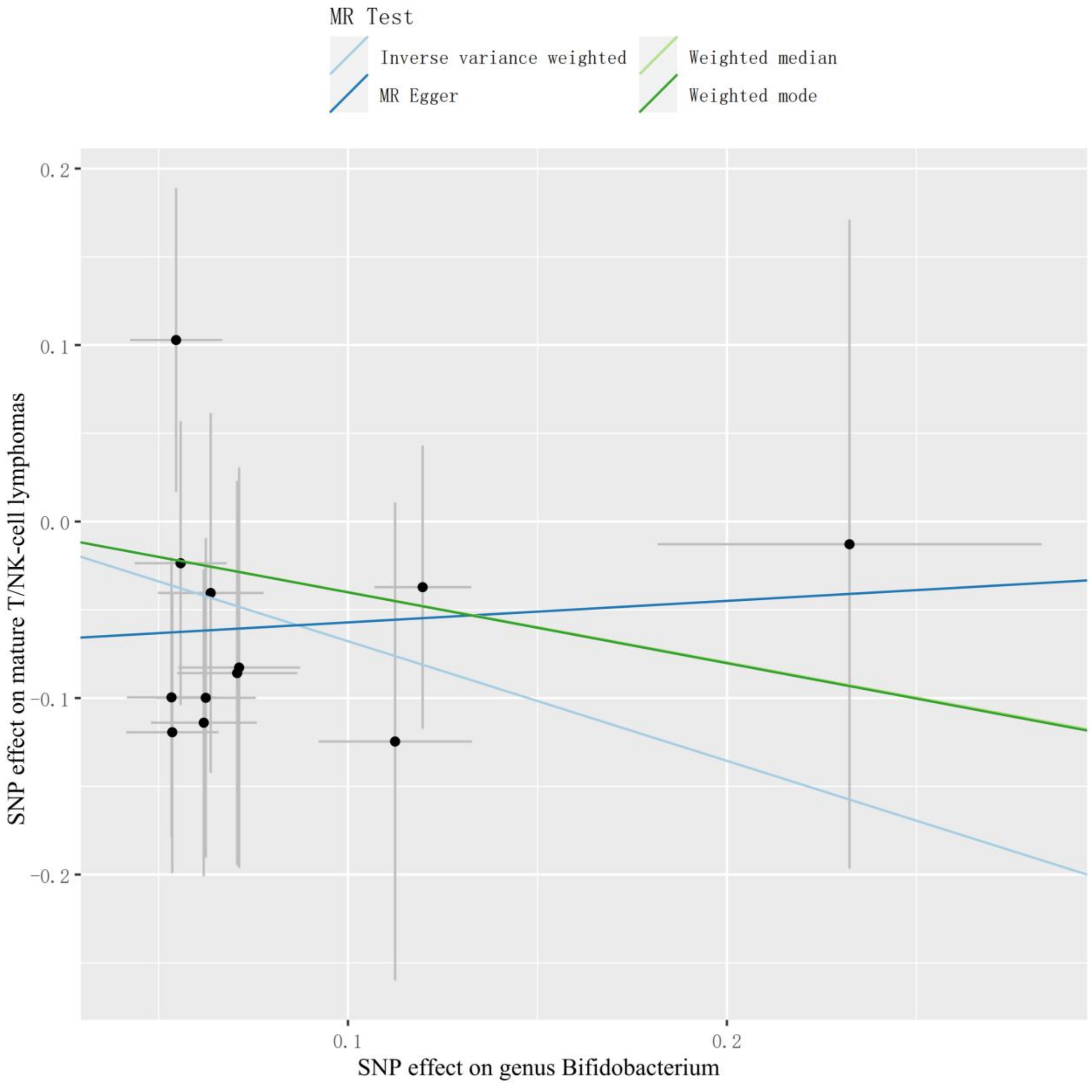
MR Test
Inverse variance weighted
MR Egger
Weighted median
Weighted mode
0.2
0.1
0.0
−0.1
−0.2
SNP effect on mature T/NK-cell lymphomas
0.1
0.2
SNP effect on genus Bifidobacterium

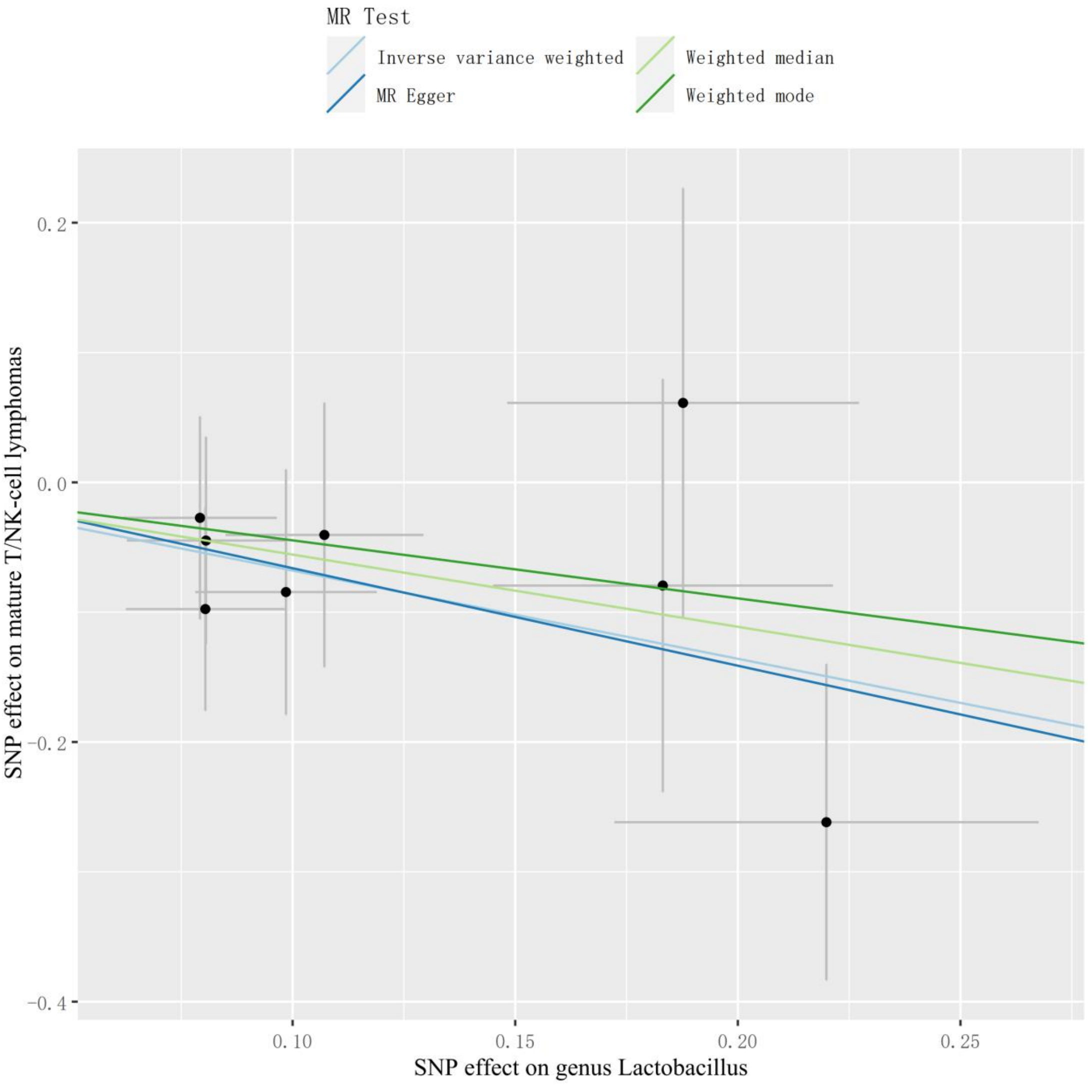
MR Test
Inverse variance weighted
MR Egger
Weighted median
Weighted mode
0.2
0.0
−0.2
−0.4
0.10
0.15
0.20
0.25
SNP effect on mature T/NK-cell lymphomas
SNP effect on genus Lactobacillus

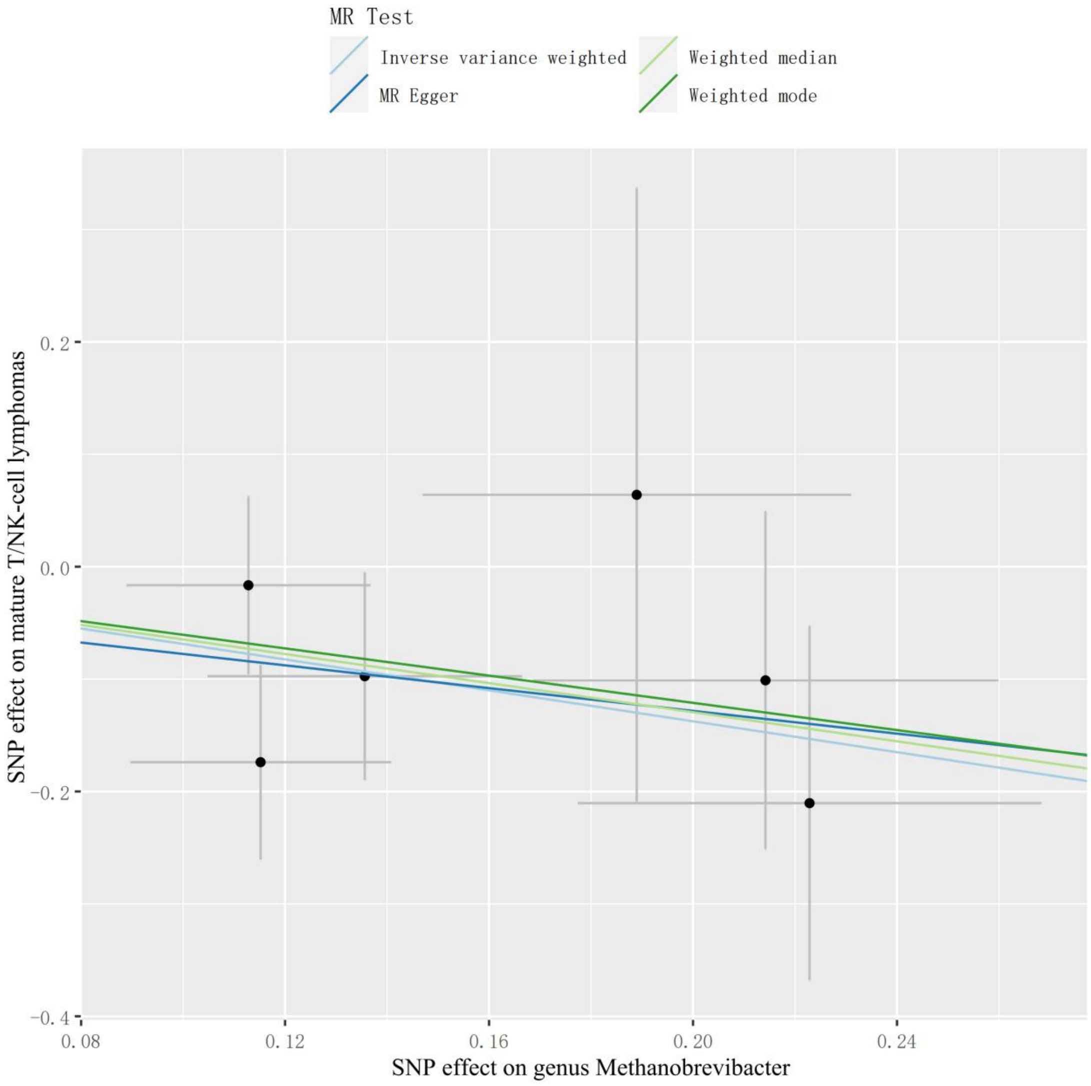
MR Test
Inverse variance weighted
MR Egger
Weighted median
Weighted mode
SNP effect on mature T/NK-cell lymphomas
0.2
0.0
−0.2
−0.4
0.08
0.12
0.16
0.20
0.24
SNP effect on genus Methanobrevibacter

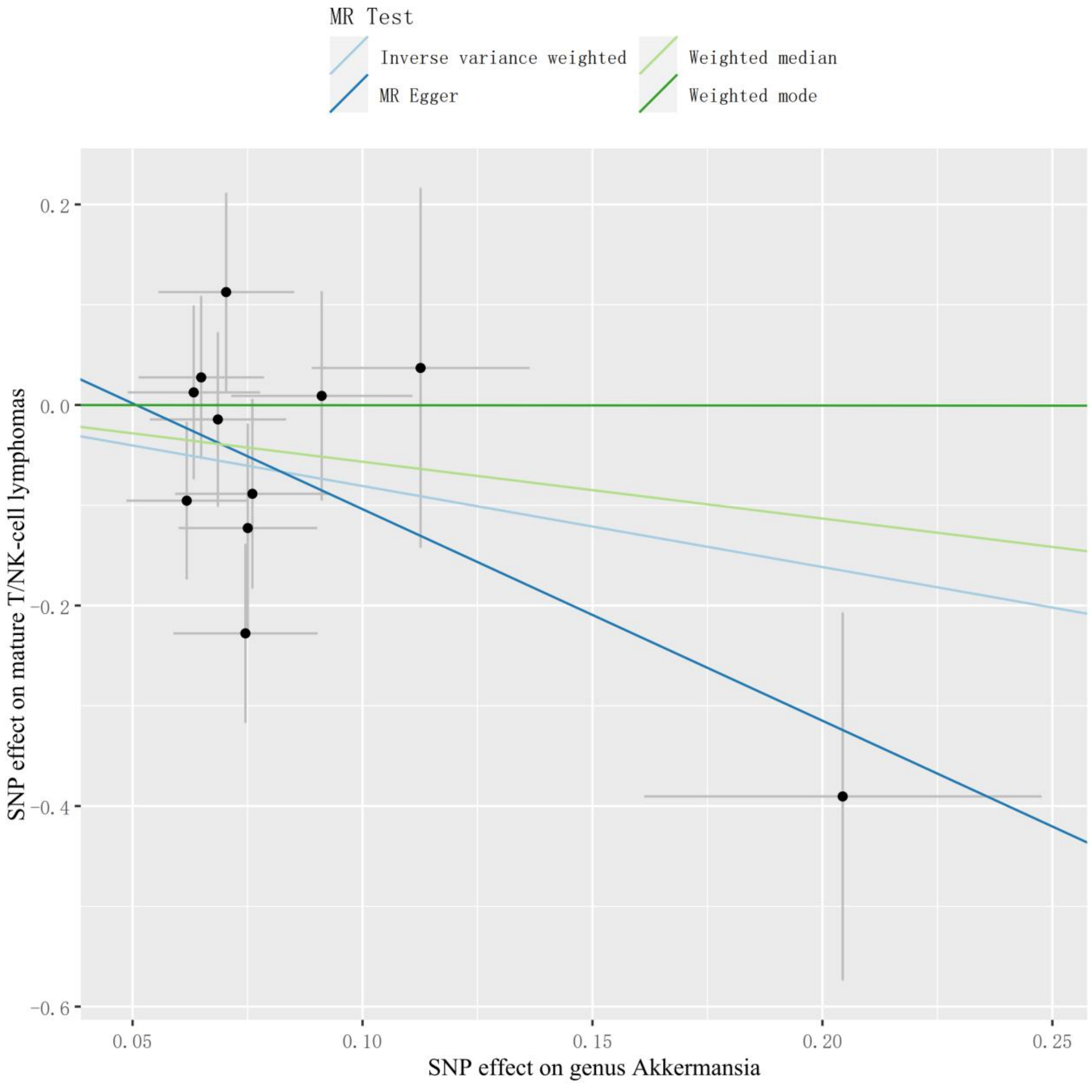

MR Test
Inverse variance weighted
MR Egger
Weighted median
Weighted mode
0.2
0.0
−0.2
−0.4
−0.6
SNP effect on mature T/NK-cell lymphomas
0.05
0.10
0.15
0.20
0.25
SNP effect on genus Akkermansia

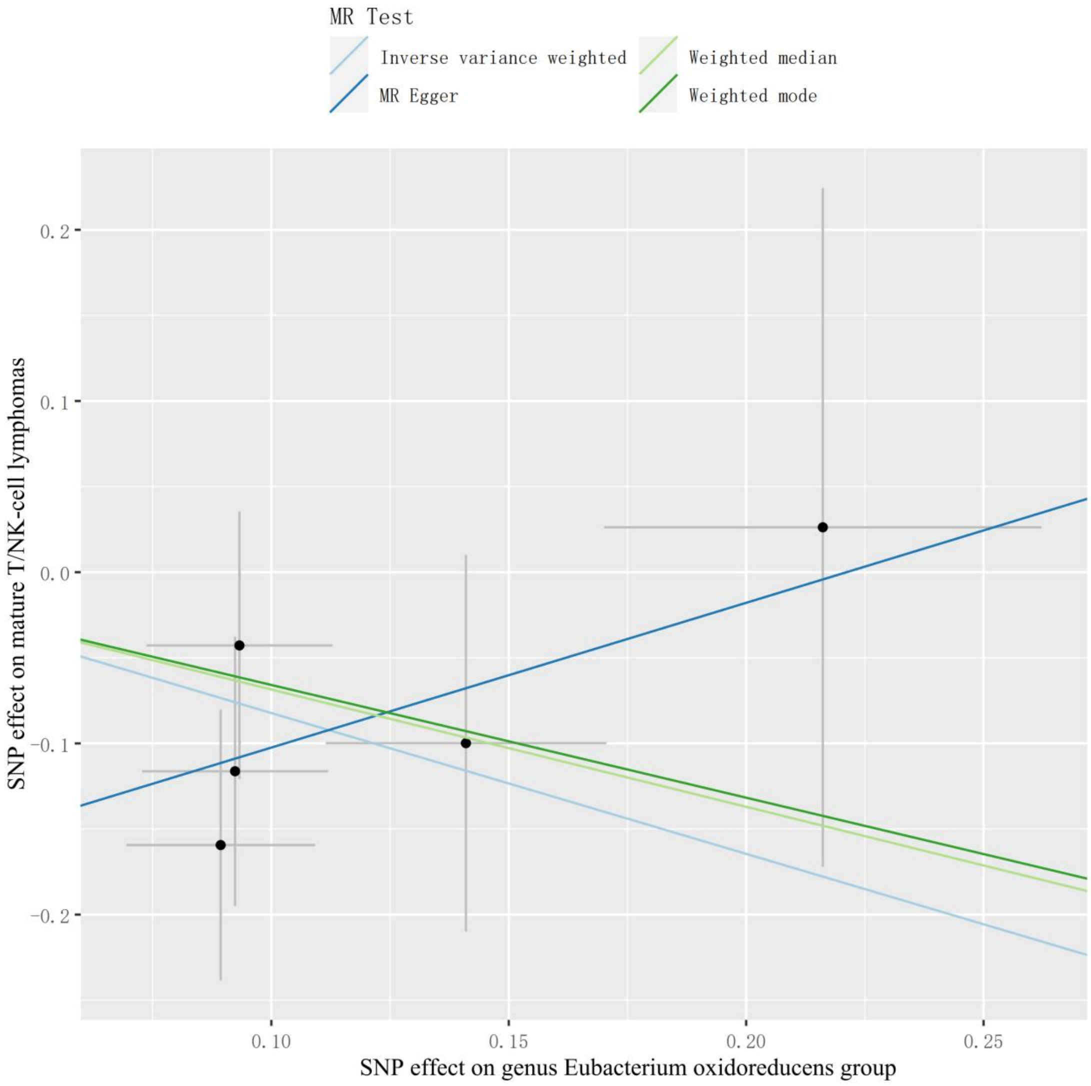

MR Test
Inverse variance weighted
MR Egger
Weighted median
Weighted mode
0.2
0.1
0.0
−0.1
−0.2
0.10
0.15
0.20
0.25
SNP effect on mature T/NK-cell lymphomas
SNP effect on genus Eubacterium oxidoreducens group

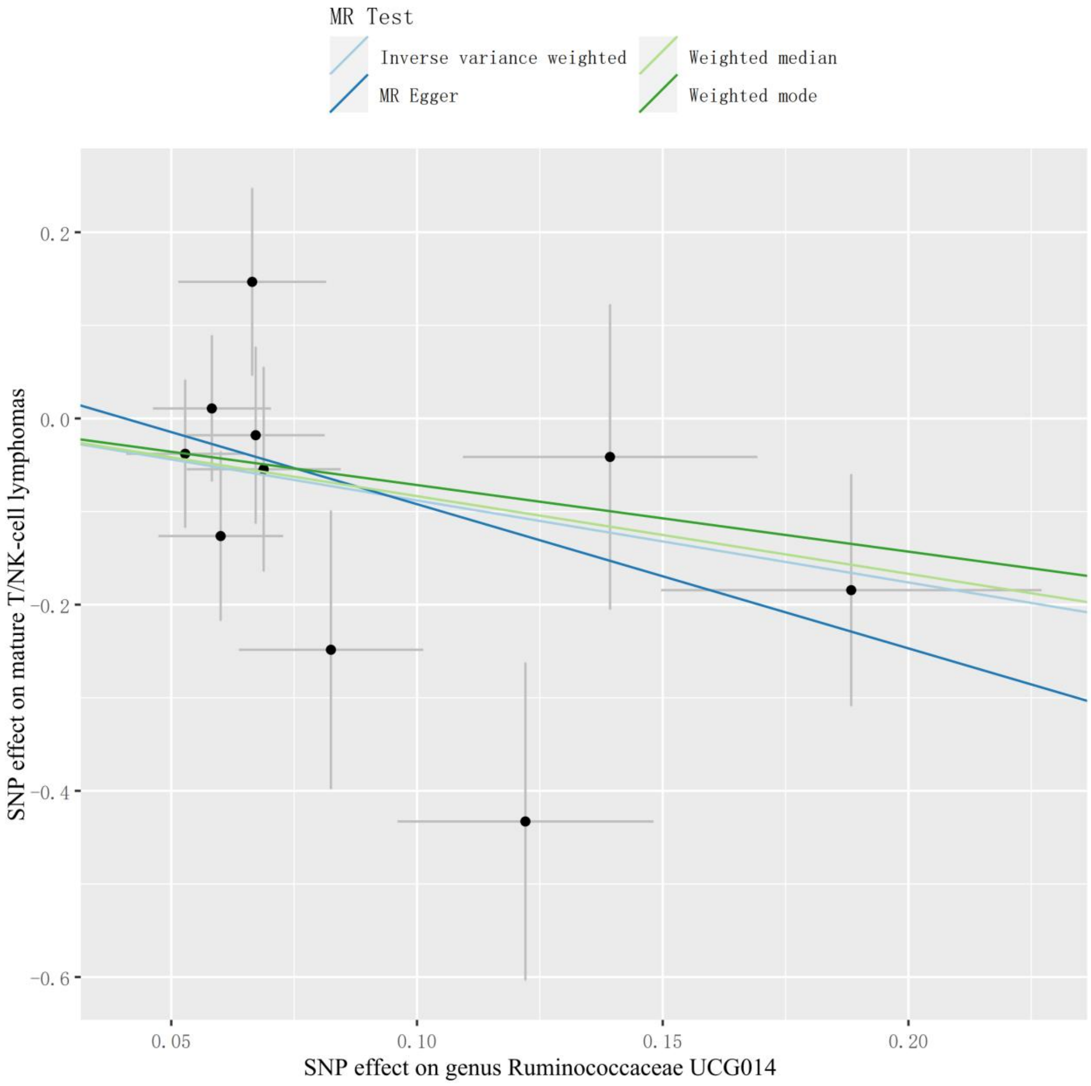
MR Test
Inverse variance weighted
MR Egger
Weighted median
Weighted mode
0.2
0.0
−0.2
−0.4
−0.6
0.05
0.10
0.15
0.20
SNP effect on mature T/NK-cell lymphomas
SNP effect on genus Ruminococcaceae UCG014

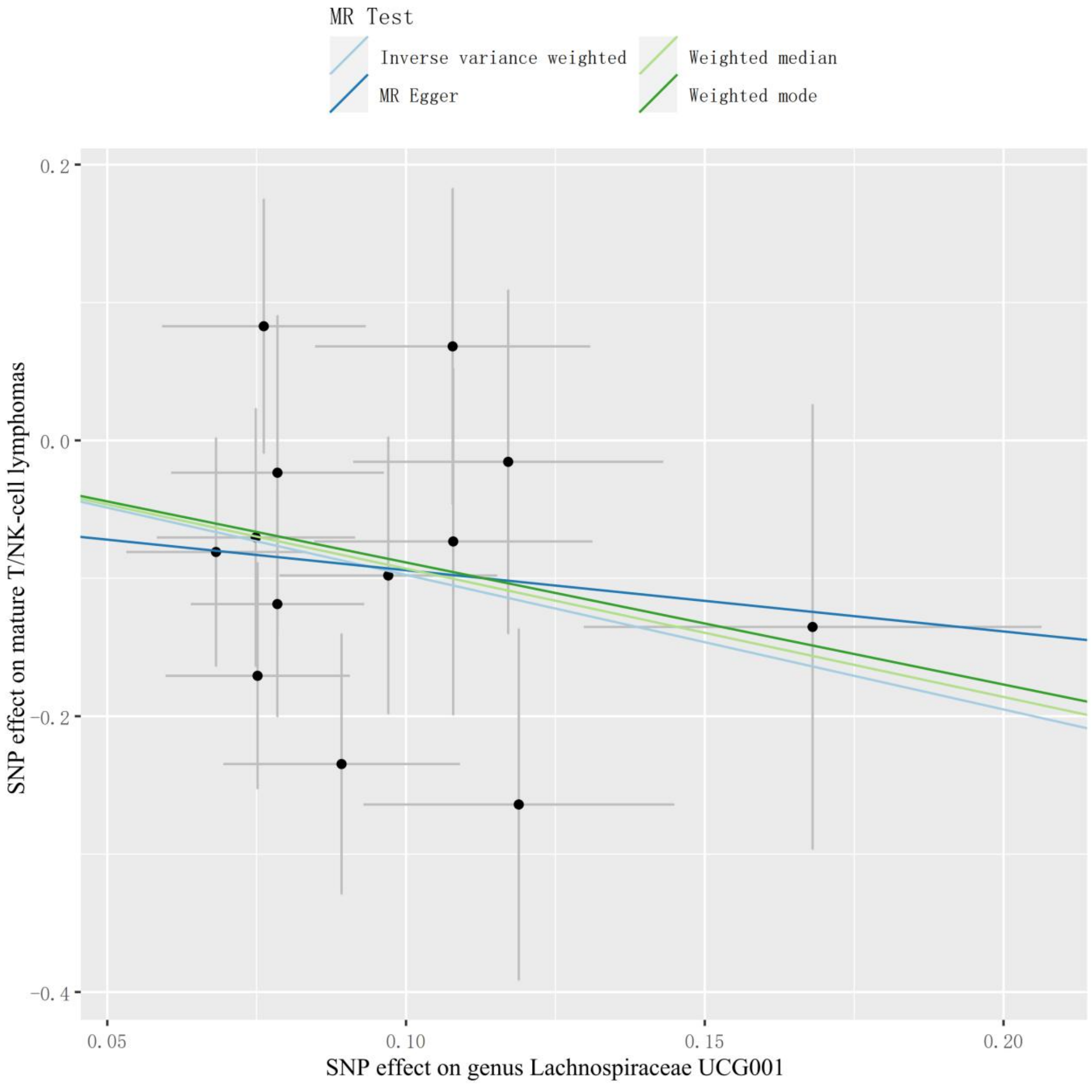

MR Test
Inverse variance weighted
MR Egger
Weighted median
Weighted mode
0.2
0.0
−0.2
−0.4
0.05
0.10
0.15
0.20
SNP effect on mature T/NK-cell lymphomas
SNP effect on genus Lachnospiraceae UCG001

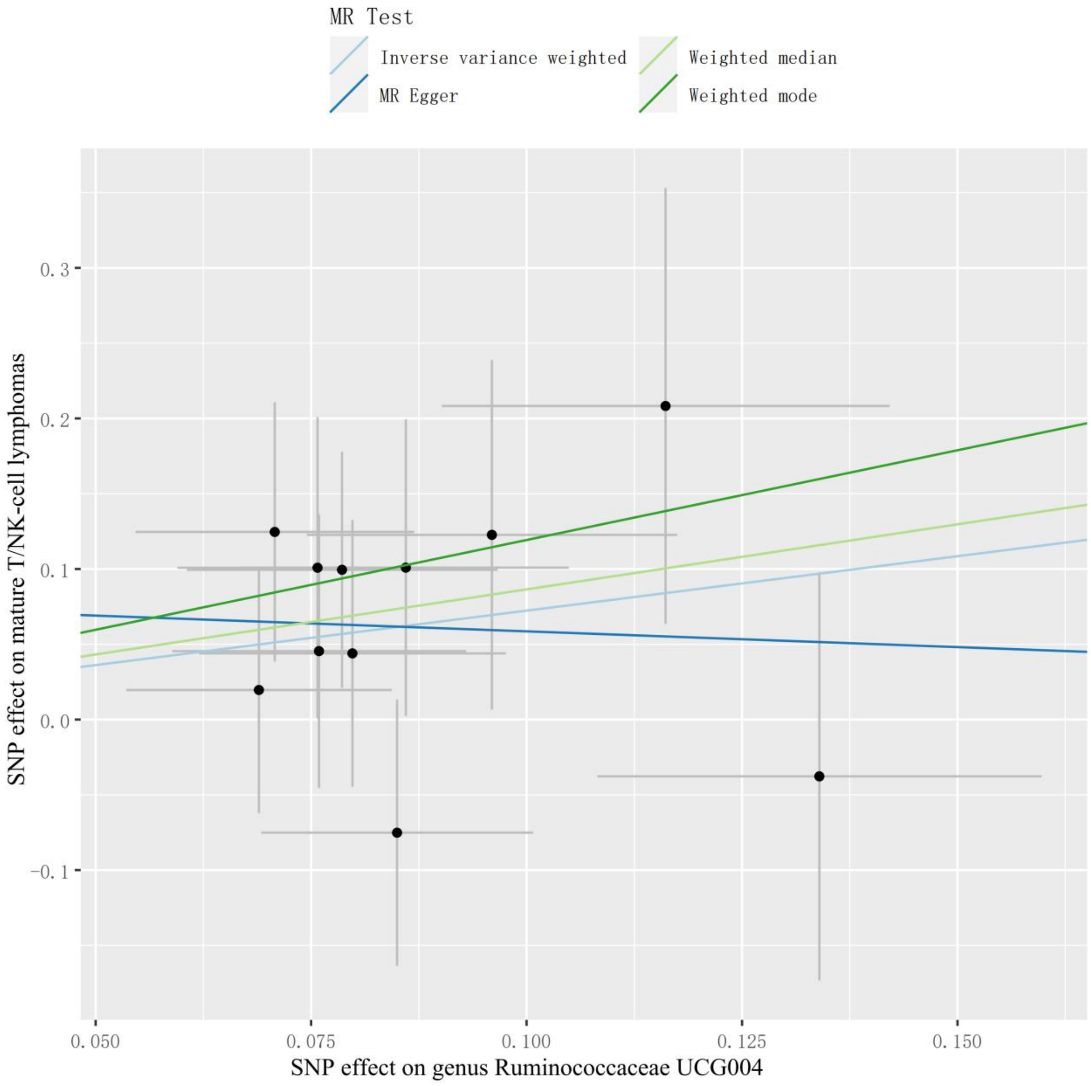

MR Test
Inverse variance weighted
MR Egger
Weighted median
Weighted mode
0.3
0.2
0.1
0.0
−0.1
SNP effect on mature T/NK-cell lymphomas
0.050
0.075
0.100
0.125
0.150
SNP effect on genus Ruminococcaceae UCG004

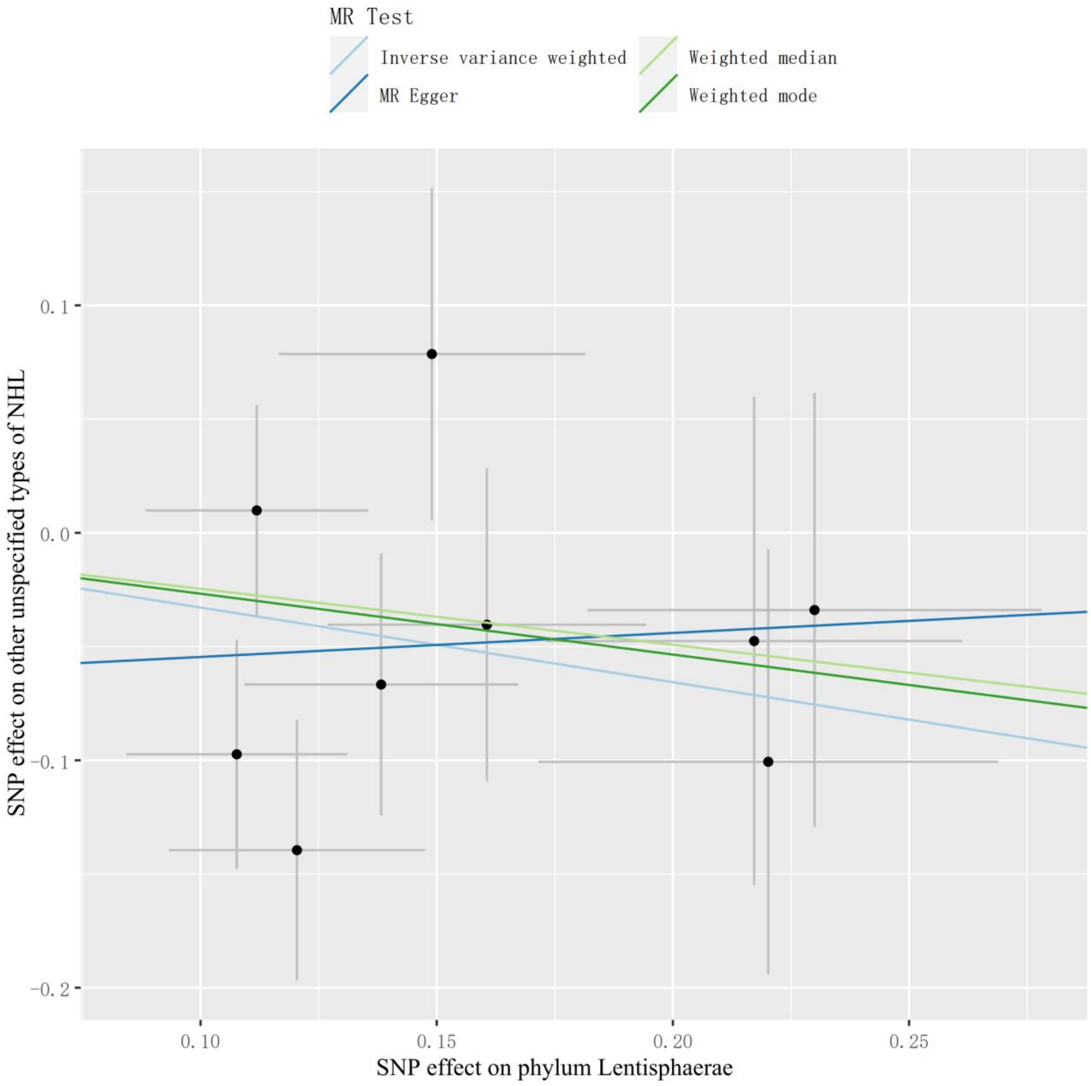

MR Test
Inverse variance weighted
MR Egger
Weighted median
Weighted mode
0.1
0.0
−0.1
−0.2
0.10
0.15
0.20
0.25
SNP effect on other unspecified types of NHL
SNP effect on phylum Lentisphaerae

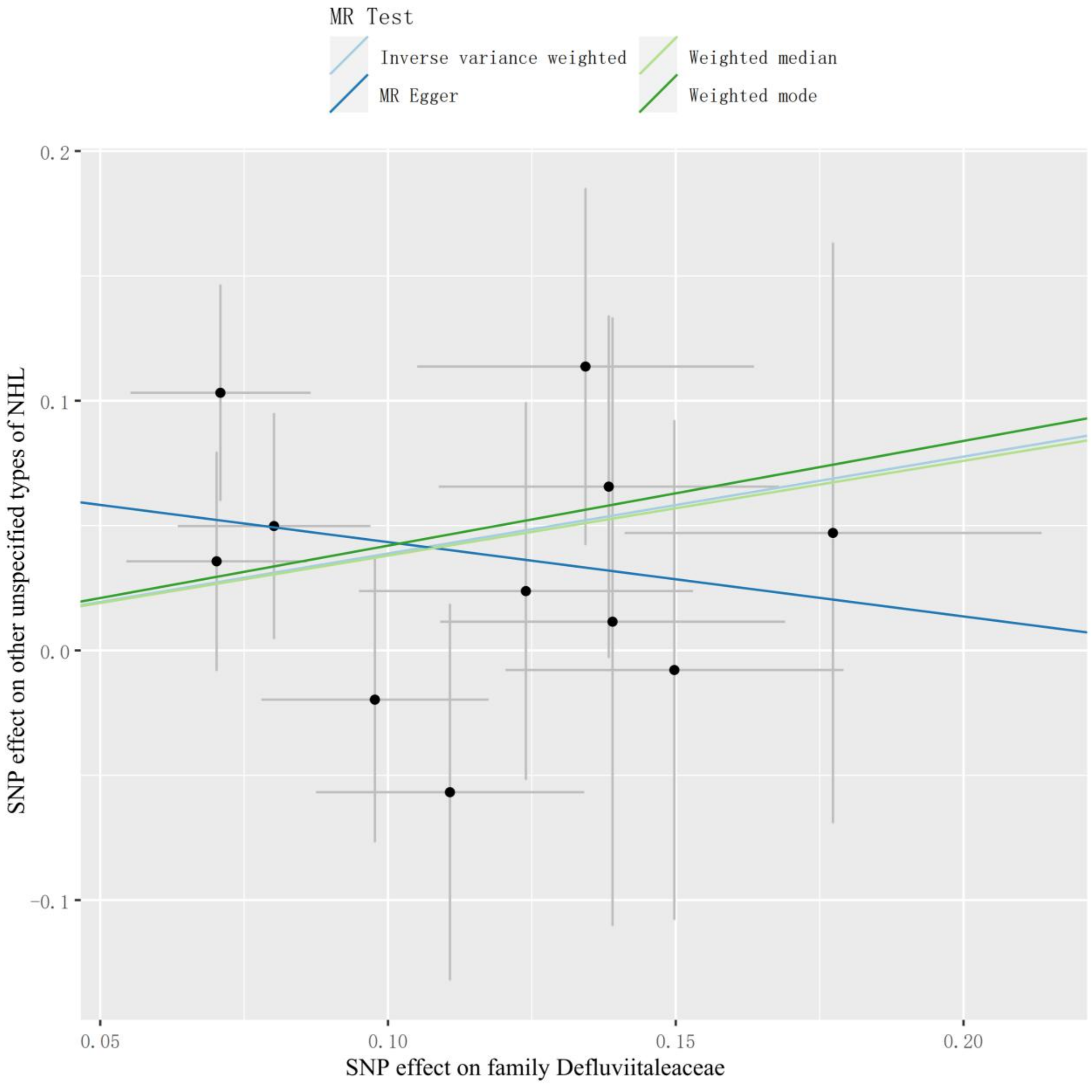
MR Test
Inverse variance weighted
MR Egger
Weighted median
Weighted mode
0.2
0.1
0.0
-0.1
SNP effect on other unspecified types of NHL
0.05
0.10
0.15
0.20
SNP effect on family Defluviitaleaceae

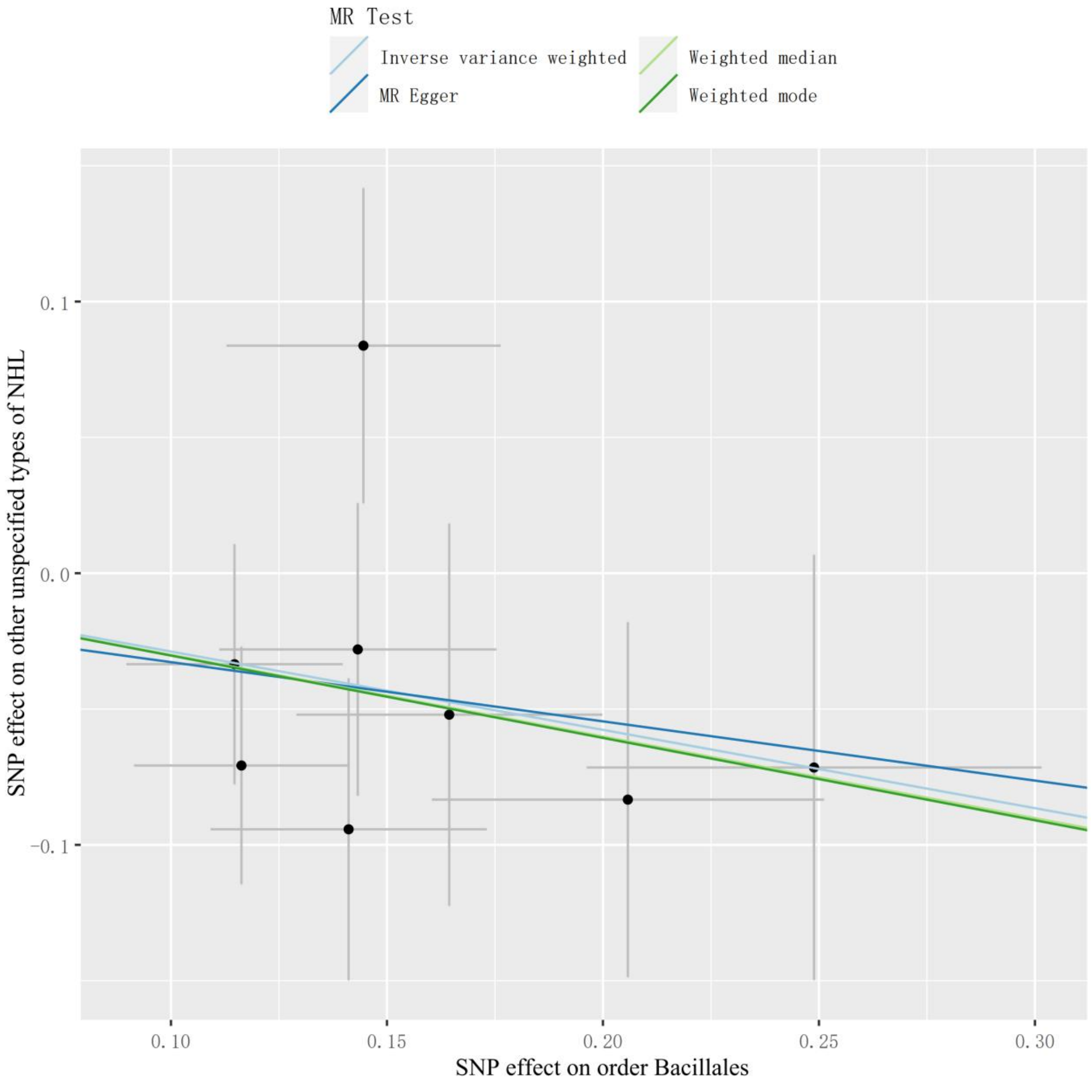

MR Test
Inverse variance weighted
MR Egger
Weighted median
Weighted mode
0.1
0.0
−0.1
0.10
0.15
0.20
0.25
0.30
SNP effect on order Bacillales
SNP effect on other unspecified types of NHL

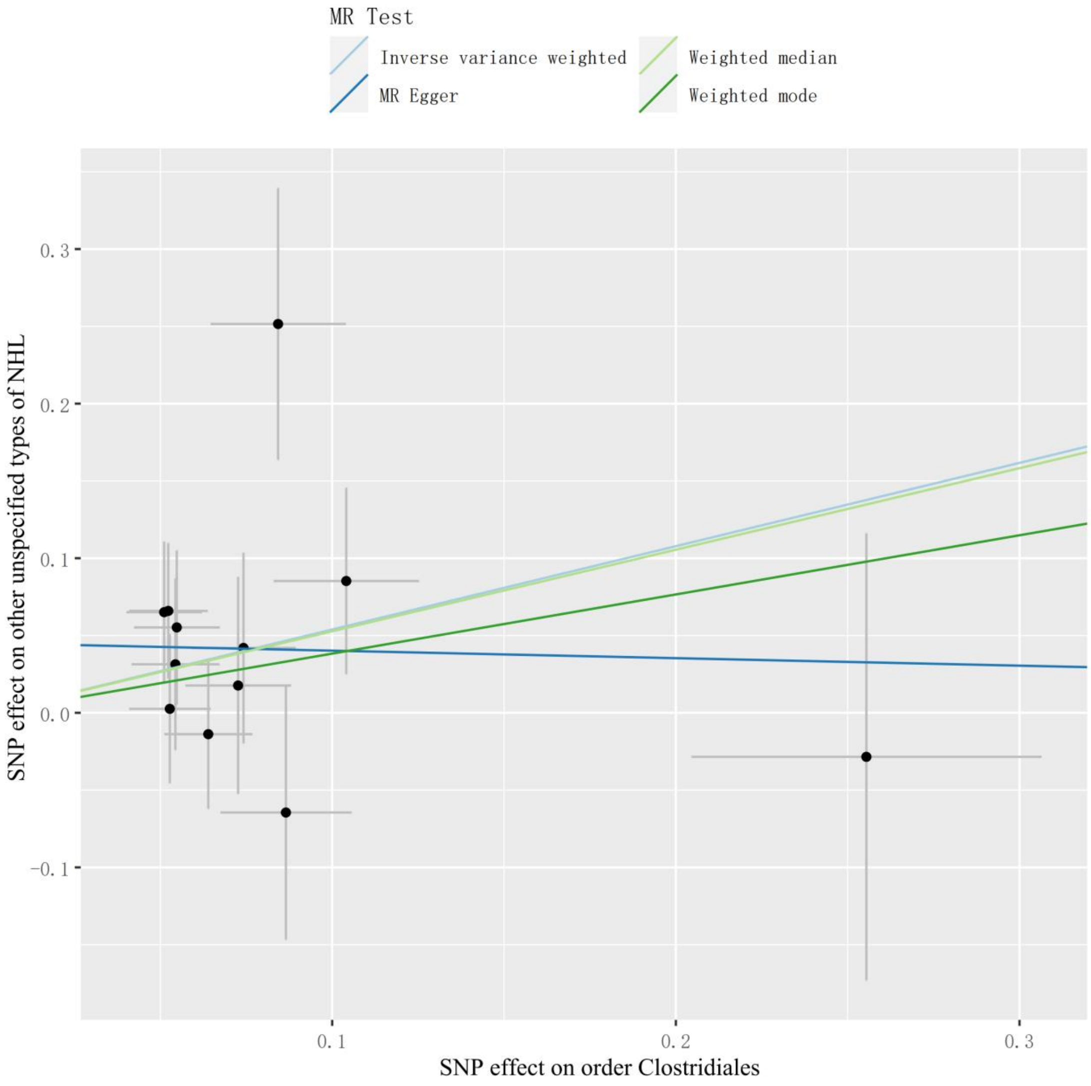
MR Test
Inverse variance weighted
MR Egger
Weighted median
Weighted mode
0.3
0.2
0.1
0.0
-0.1
SNP effect on other unspecified types of NHL
0.1
0.2
0.3
SNP effect on order Clostridiales

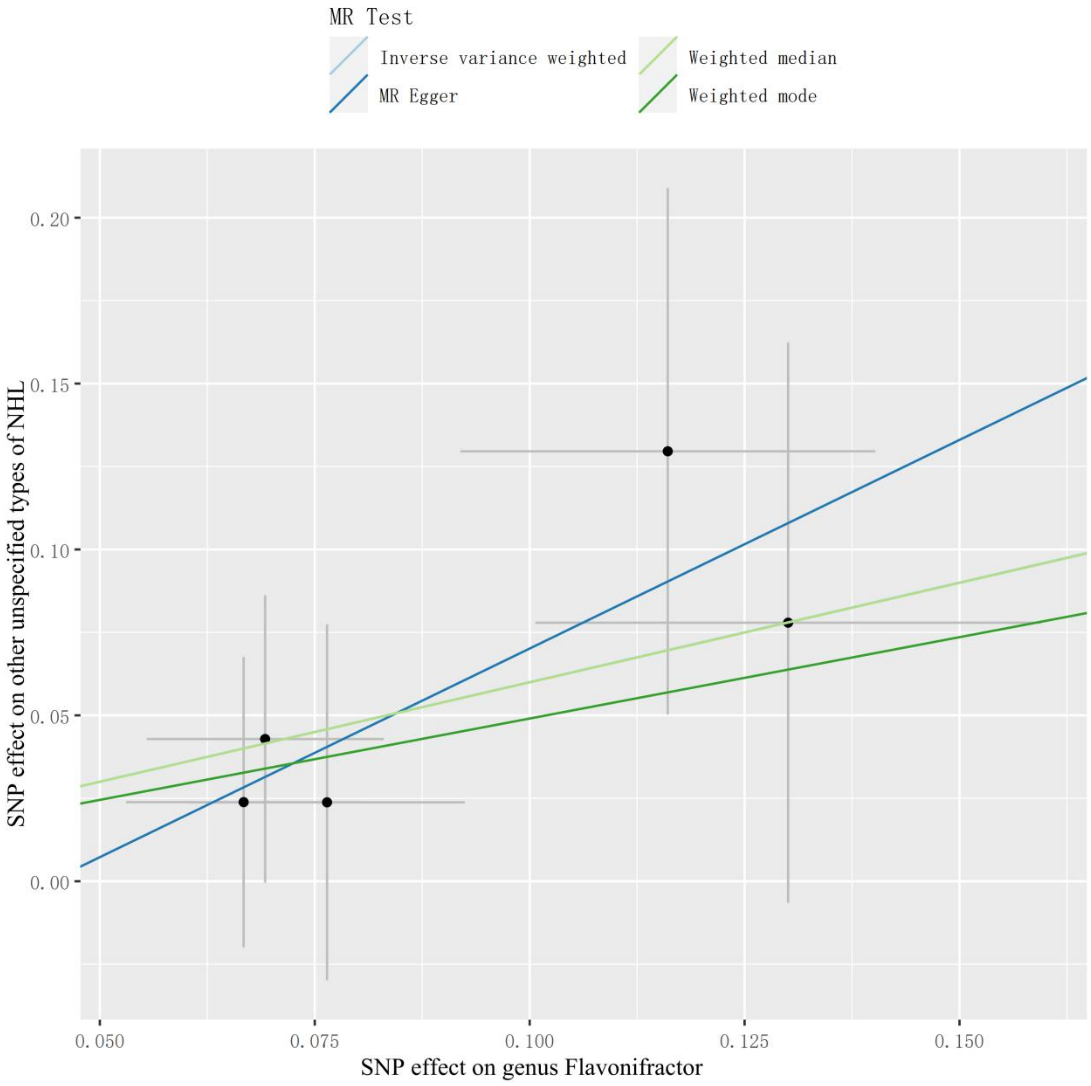
MR Test
Inverse variance weighted
MR Egger
Weighted median
Weighted mode
SNP effect on other unspecified types of NHL
0.20
0.15
0.10
0.05
0.00
0.050
0.075
0.100
0.125
0.150
SNP effect on genus Flavonifractor

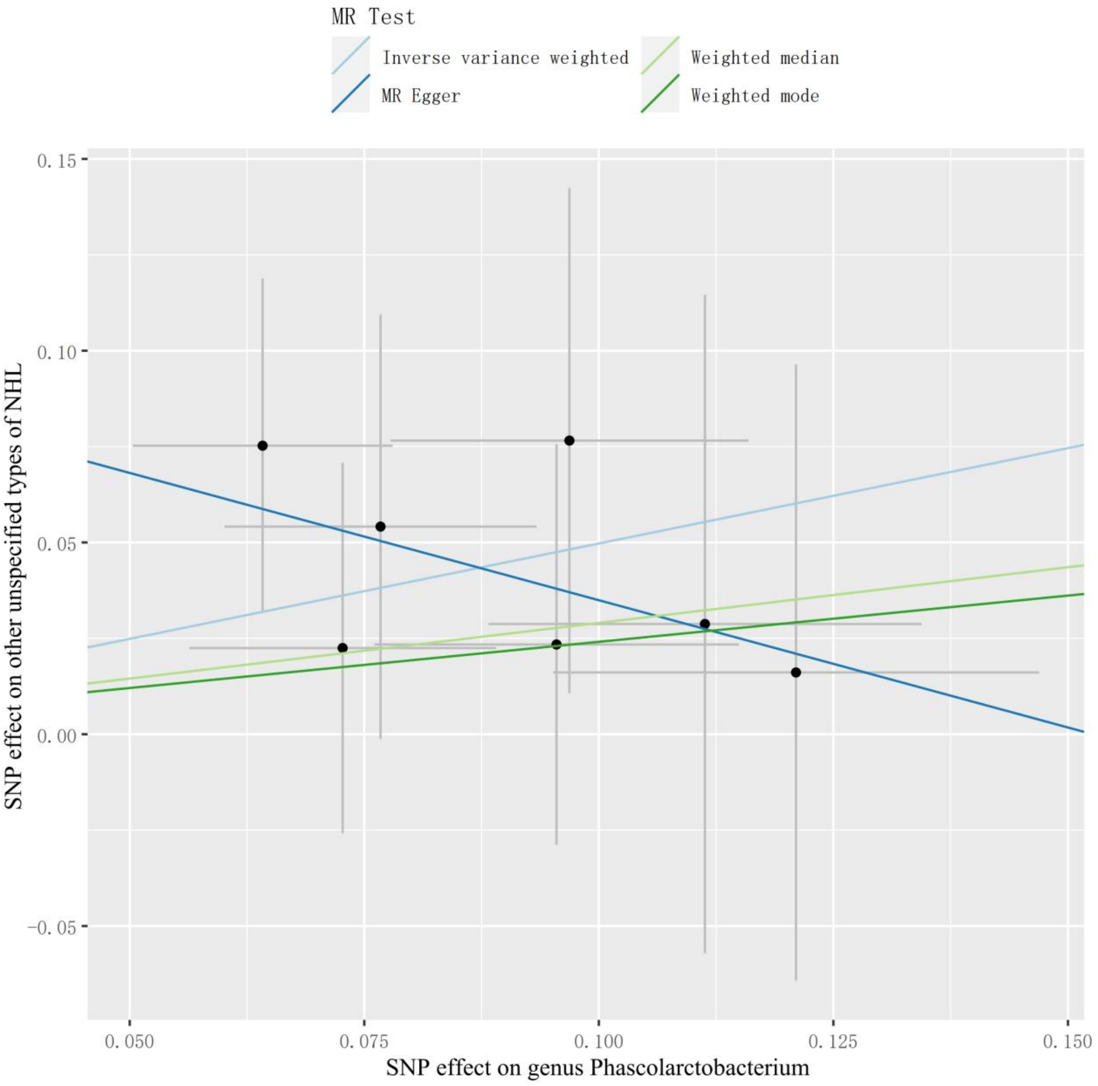
MR Test
Inverse variance weighted
MR Egger
Weighted median
Weighted mode
0.15
0.10
0.05
0.00
−0.05
SNP effect on other unspecified types of NHL
0.050
0.075
0.100
0.125
0.150
SNP effect on genus Phascolarctobacterium

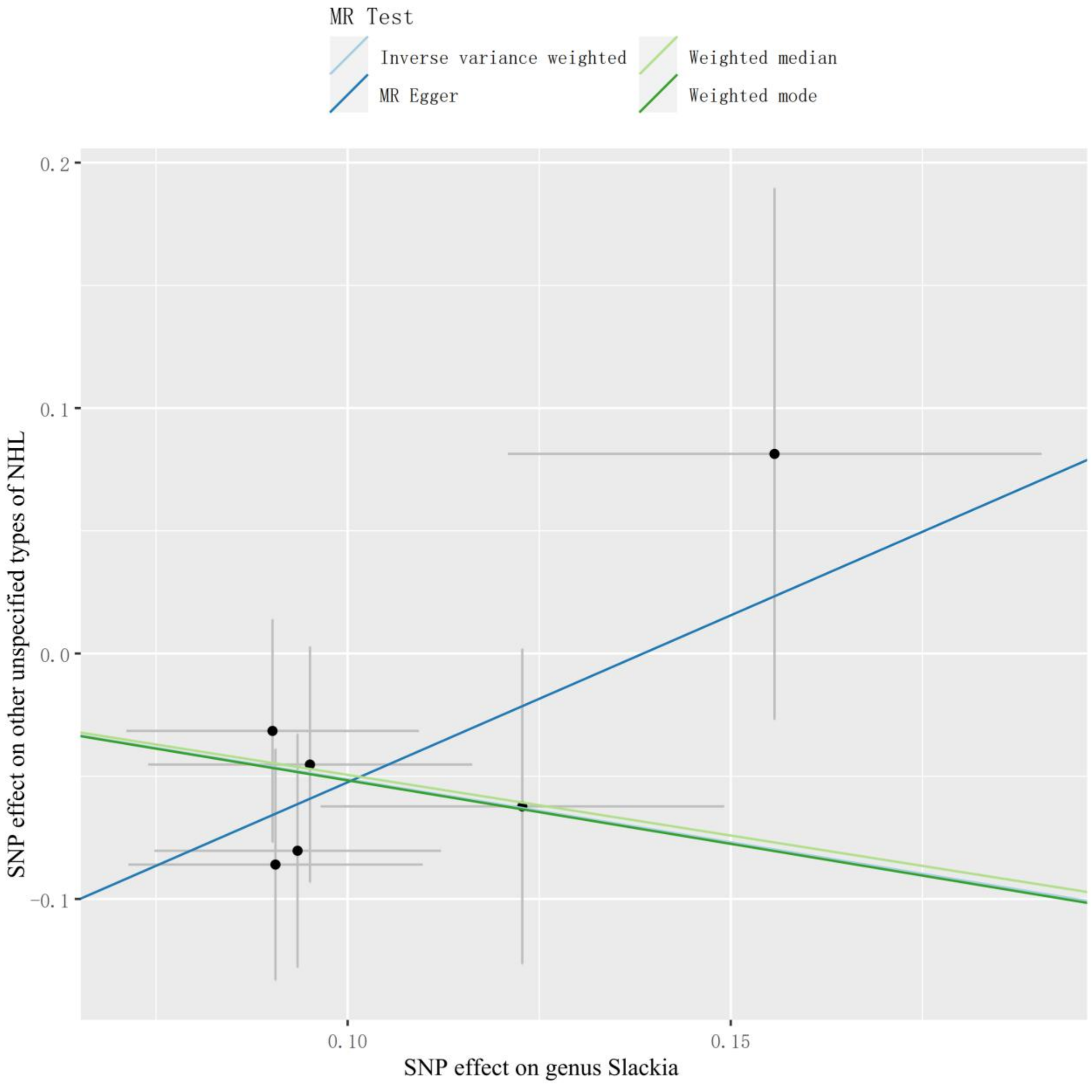

MR Test
Inverse variance weighted
MR Egger
Weighted median
Weighted mode
0.2
0.1
0.0
−0.1
0.10
0.15
SNP effect on genus Slackia
SNP effect on other unspecified types of NHL

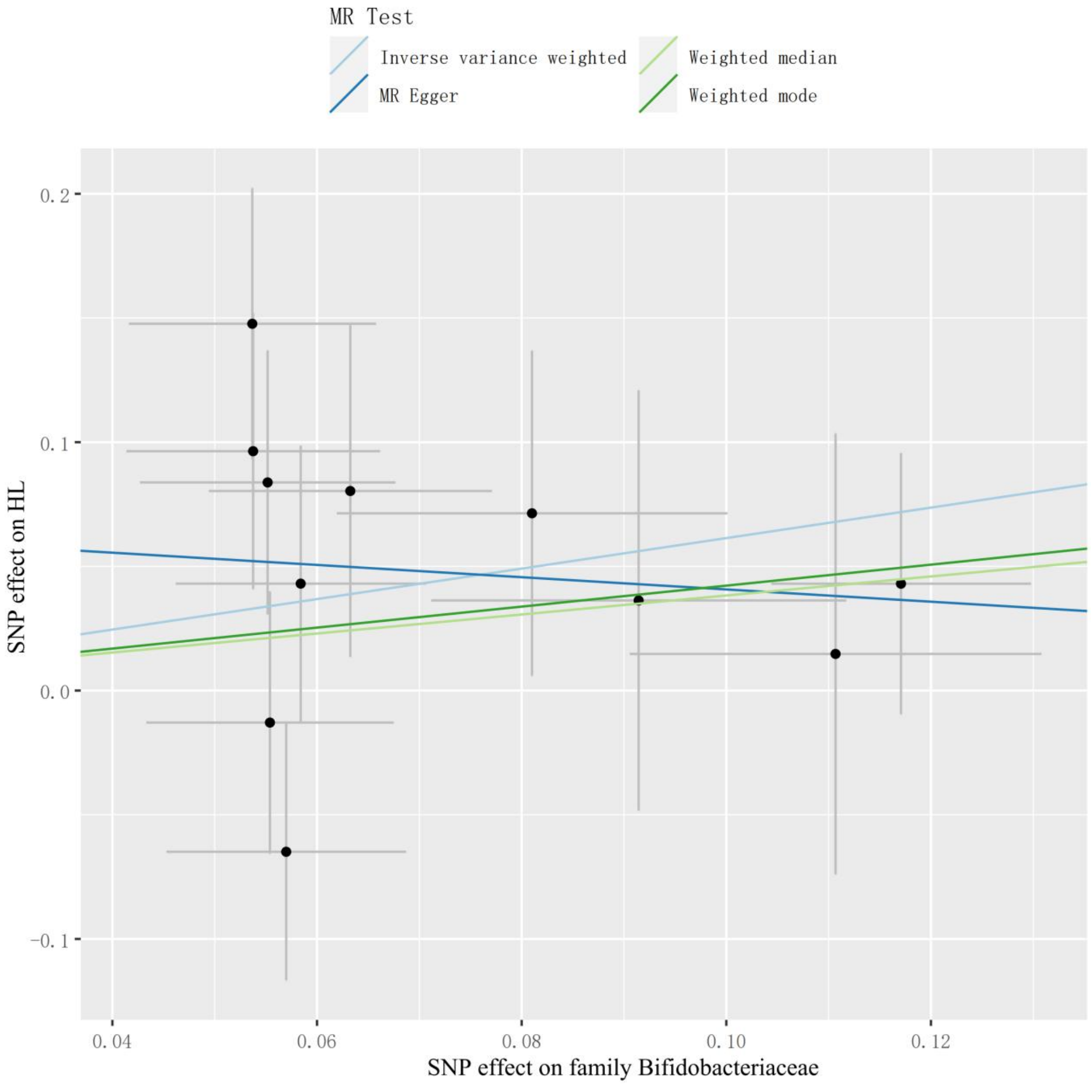

MR Test
Inverse variance weighted
MR Egger
Weighted median
Weighted mode
0.2
0.1
0.0
−0.1
0.04
0.06
0.08
0.10
0.12
SNP effect on HL
SNP effect on family Bifidobacteriaceae

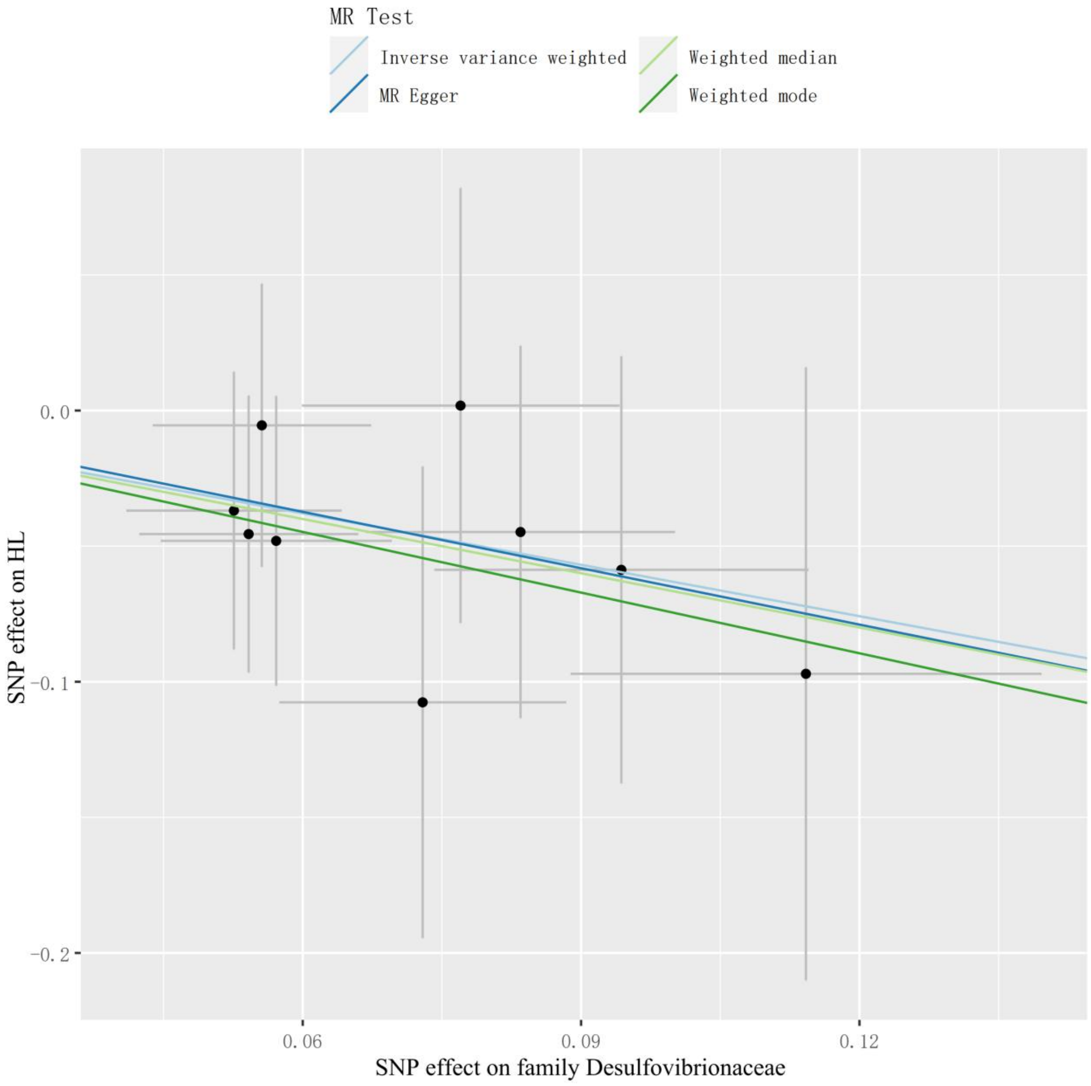
MR Test
Inverse variance weighted
MR Egger
Weighted median
Weighted mode
0.0
-0.1
-0.2
0.06
0.09
0.12
SNP effect on HL
SNP effect on family Desulfovibrionaceae

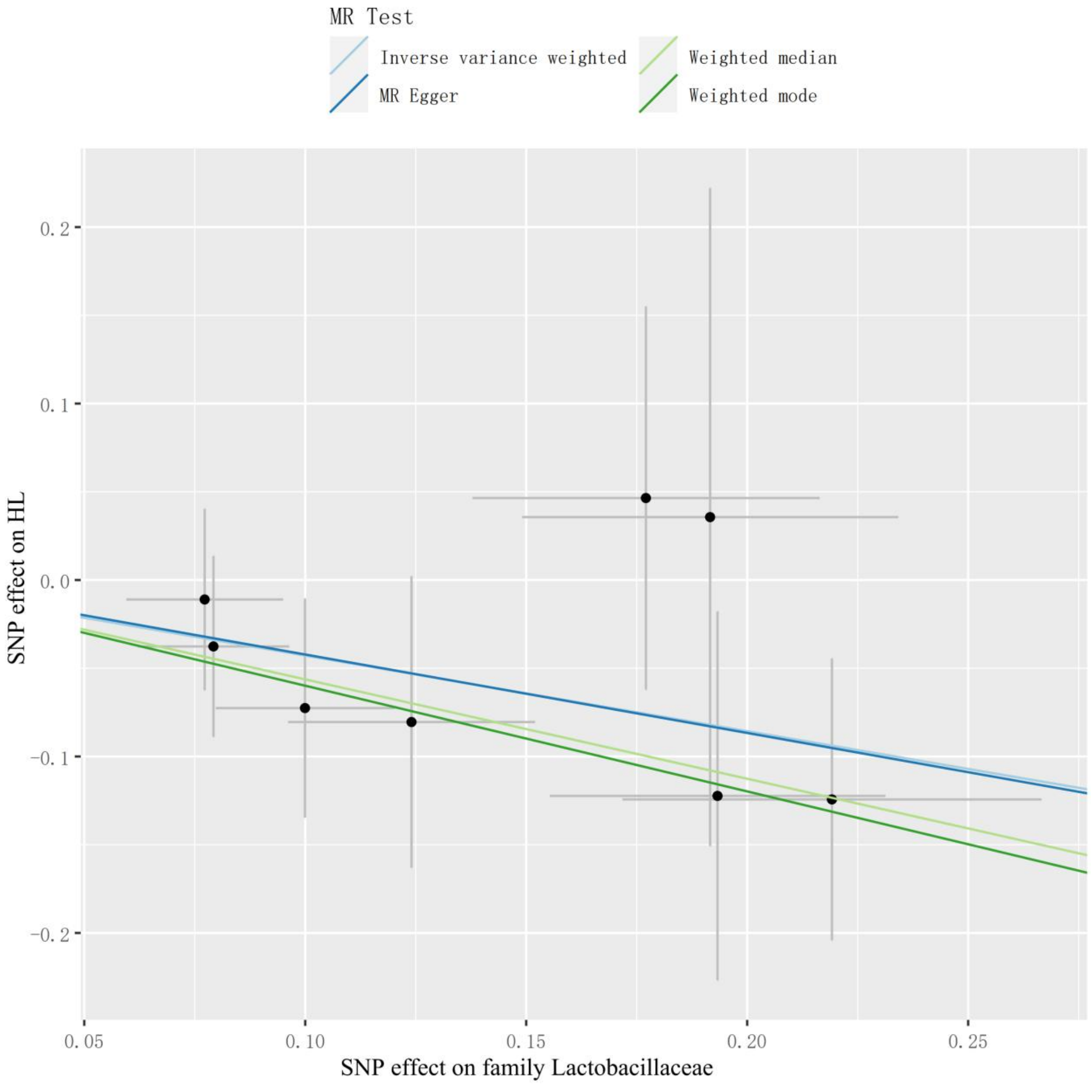
MR Test
Inverse variance weighted
MR Egger
Weighted median
Weighted mode
0.2
0.1
0.0
−0.1
−0.2
SNP effect on HL
0.05
0.10
0.15
0.20
0.25
SNP effect on family Lactobacillaceae

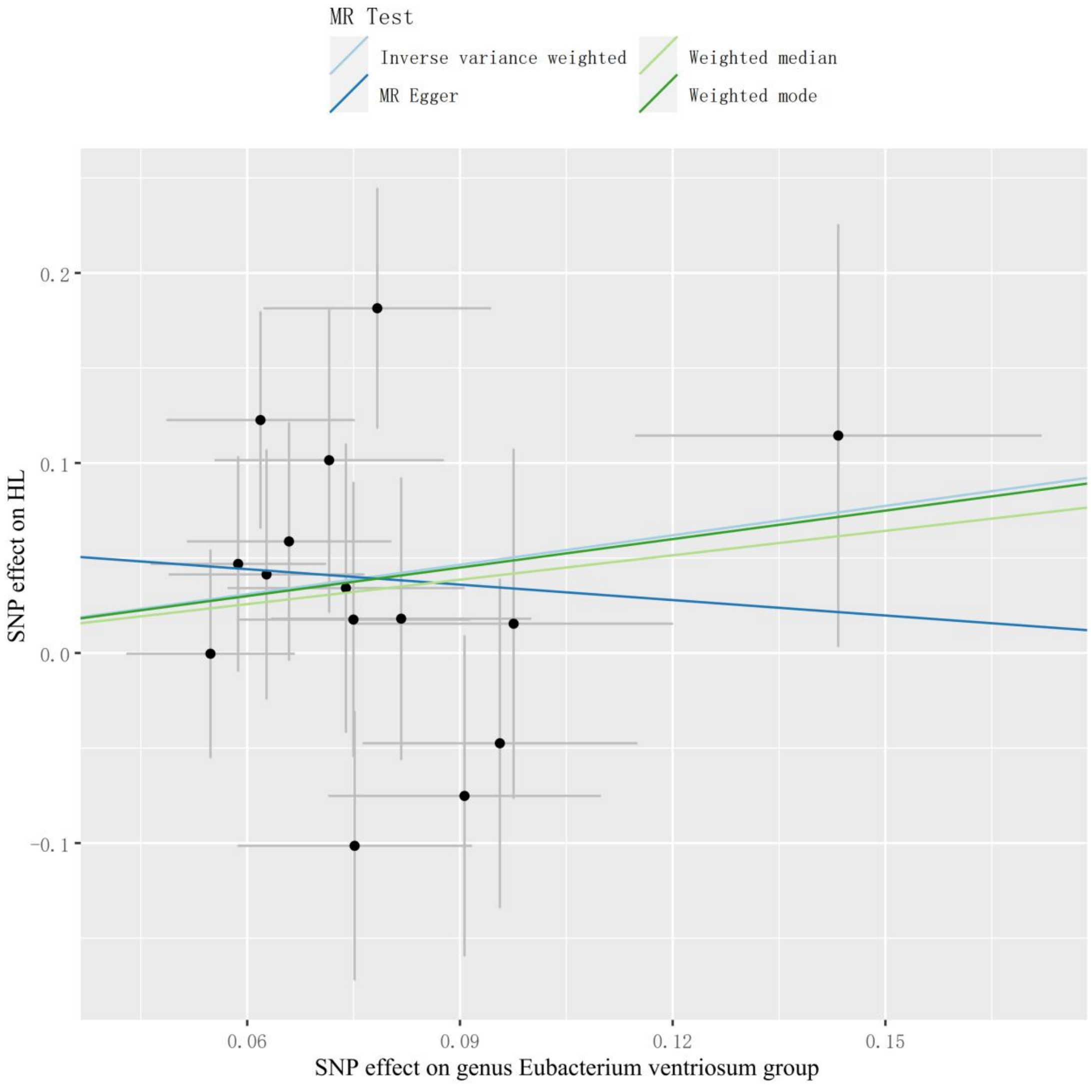

MR Test
Inverse variance weighted
MR Egger
Weighted median
Weighted mode
0.2
0.1
0.0
−0.1
SNP effect on HL
0.06
0.09
0.12
0.15
SNP effect on genus Eubacterium ventriosum group

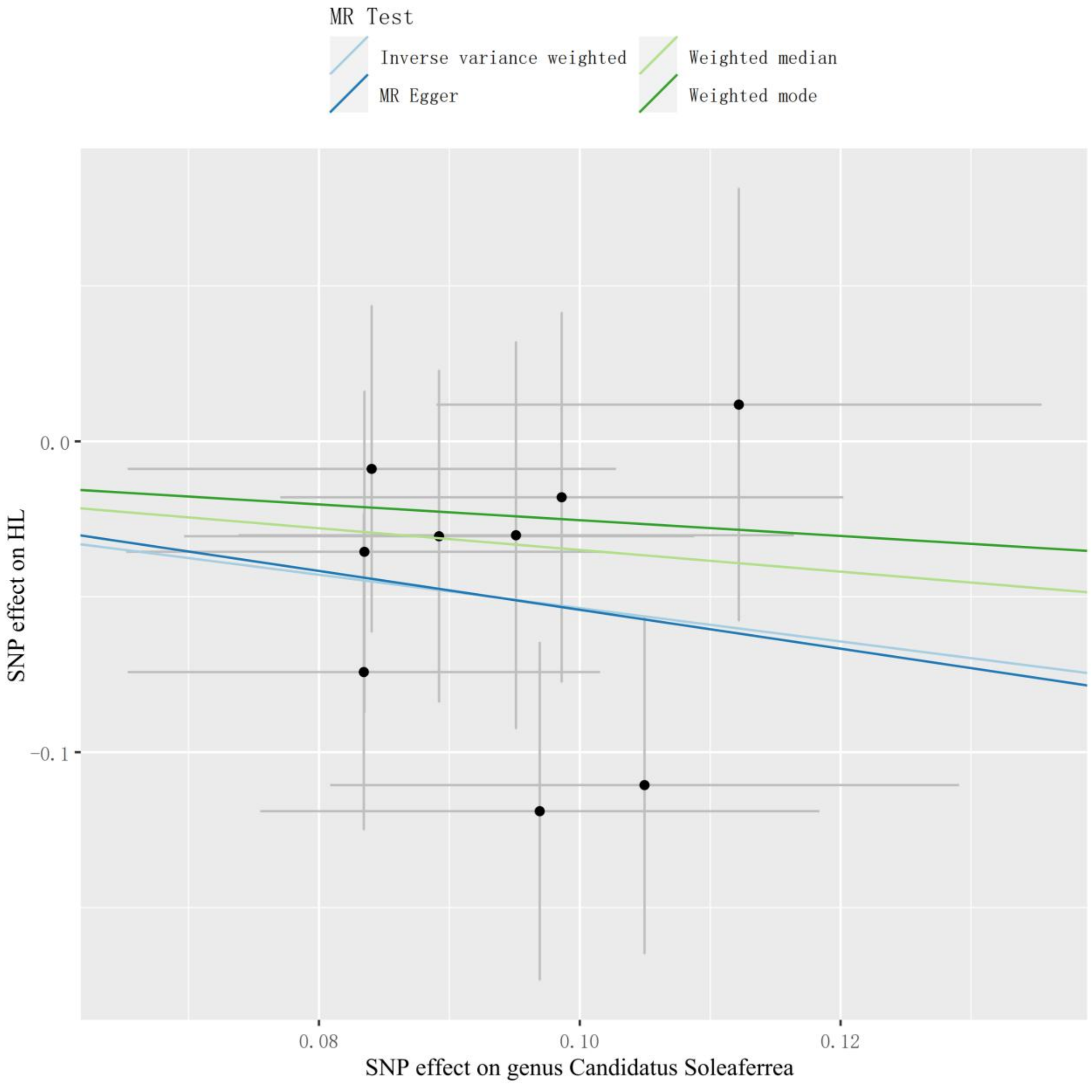
MR Test
Inverse variance weighted
MR Egger
Weighted median
Weighted mode
0.0
-0.1
SNP effect on HL
0.08
0.10
0.12
SNP effect on genus Candidatus Soleaferrea

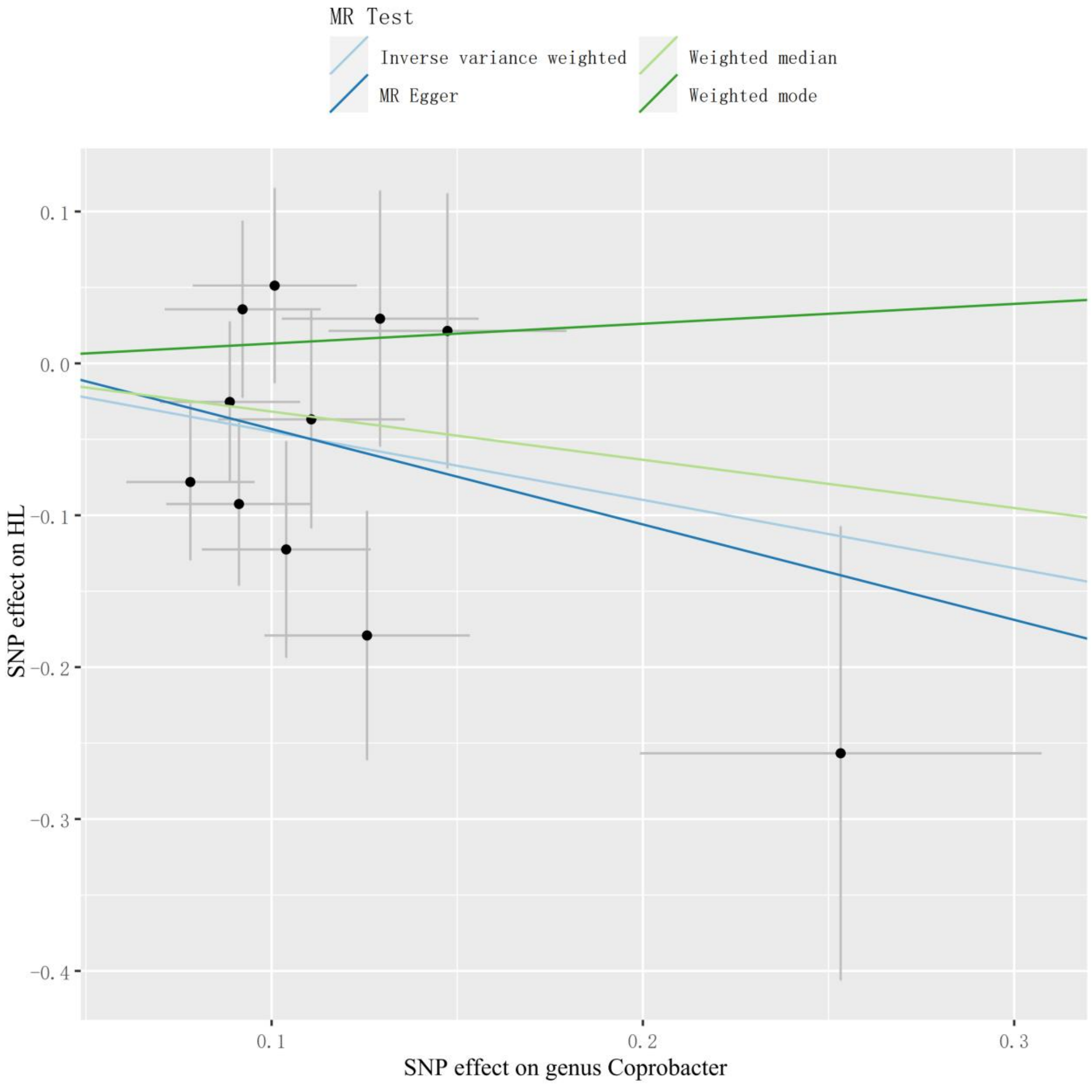
MR Test
Inverse variance weighted
MR Egger
Weighted median
Weighted mode
0.1
0.0
−0.1
−0.2
−0.3
−0.4
SNP effect on HL
0.1
0.2
0.3
SNP effect on genus Coprobacter
