# Supplementary figures and images for: Causal relationships between gut microbiota and lymphoma: a bidirectional Mendelian randomization study
Source: Front Cell Infect Microbiol. 2024 May 13;14:1374775. doi: 10.3389/fcimb.2024.1374775 (PMC11128559; doi:10.3389/fcimb.2024.1374775)

Supplementary Figure S2. MR “leave-one-out” sensitivity analysis of gut microbiota on lymphoma.

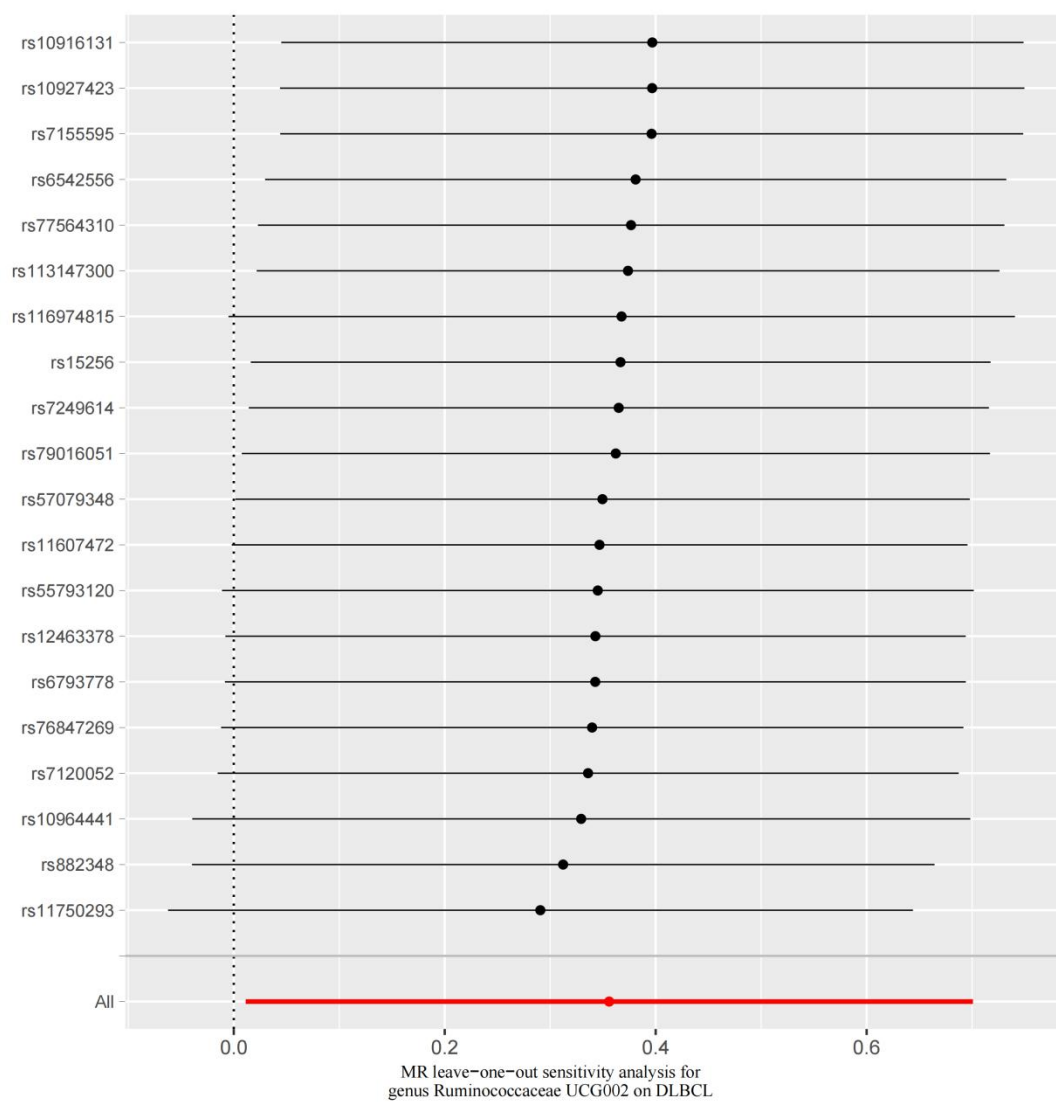

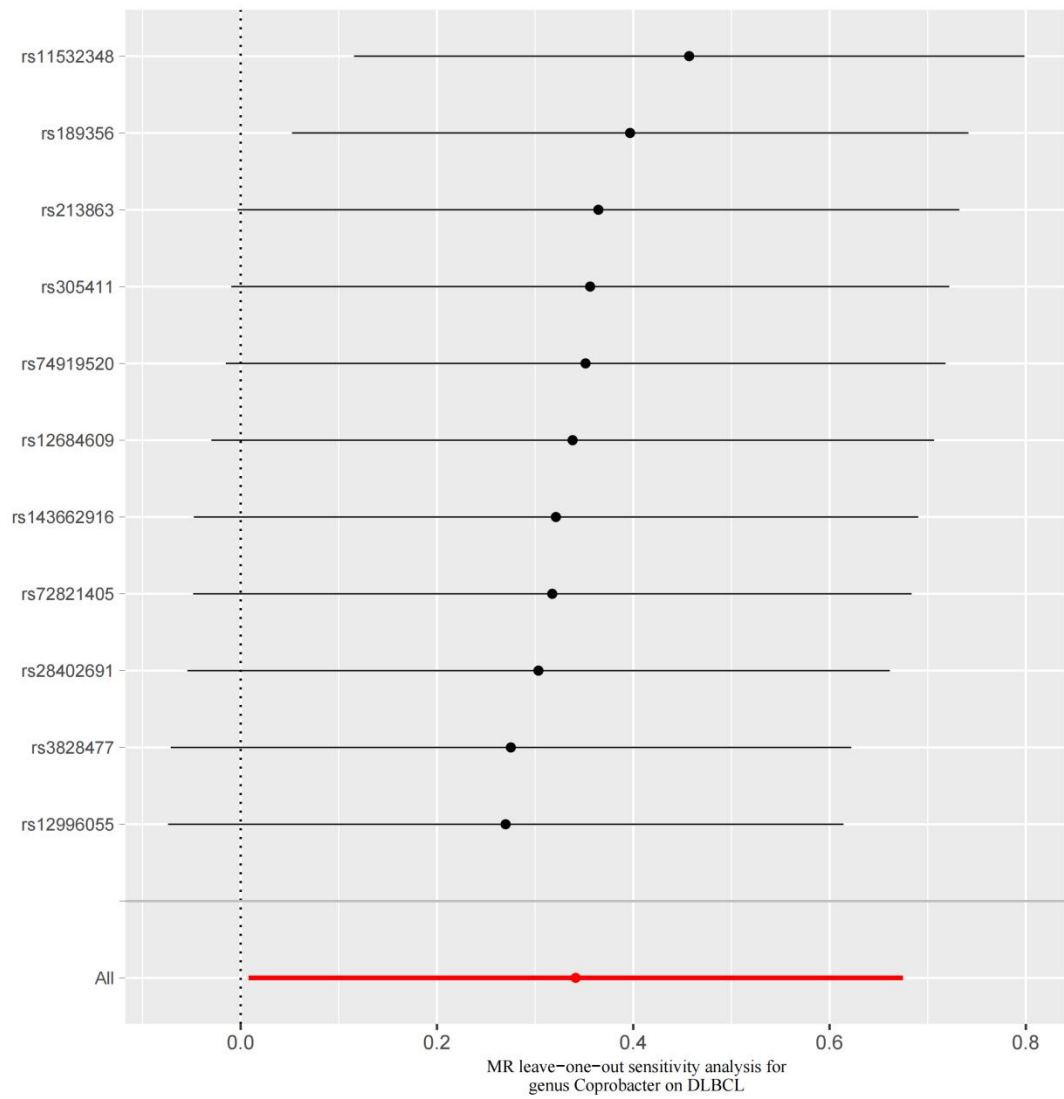

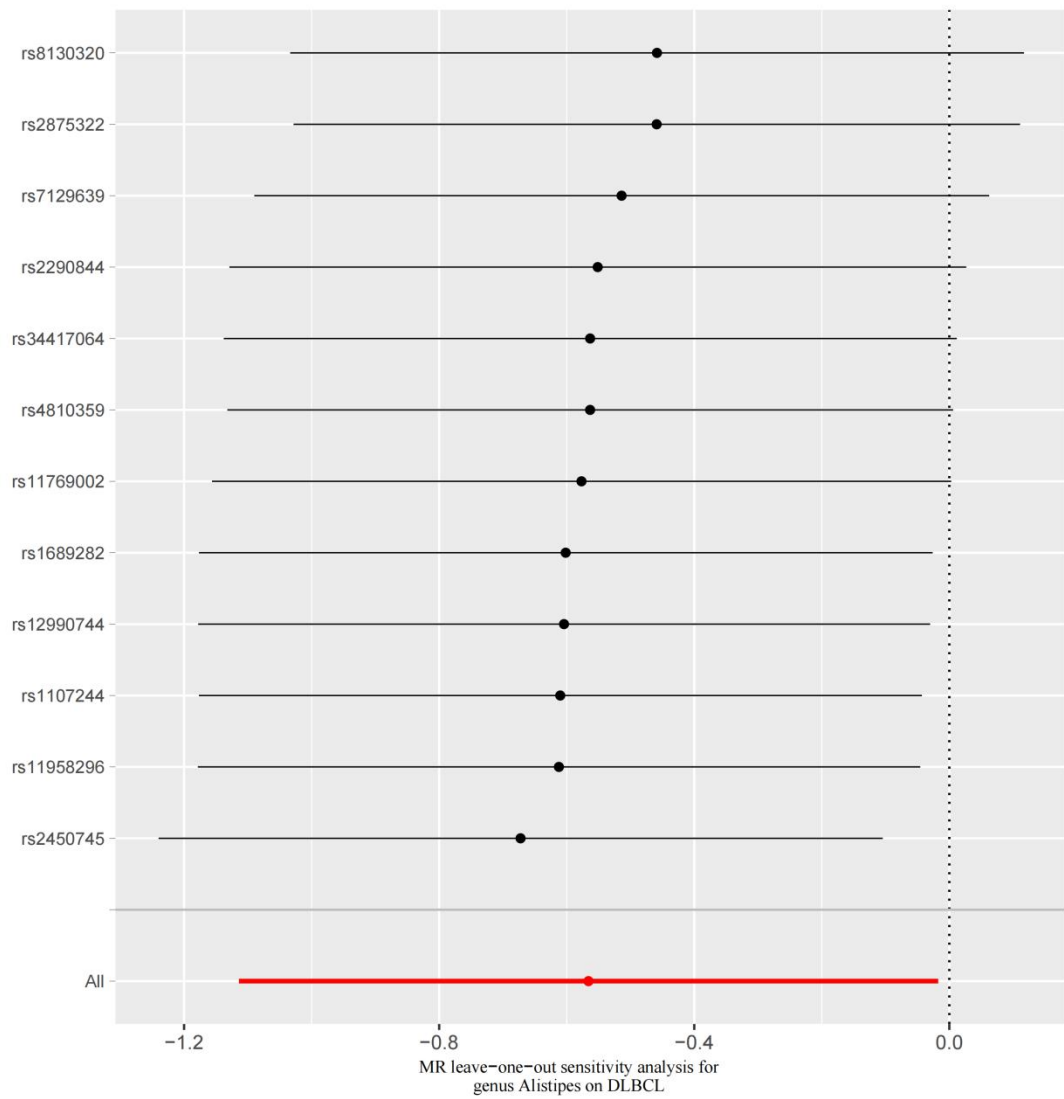

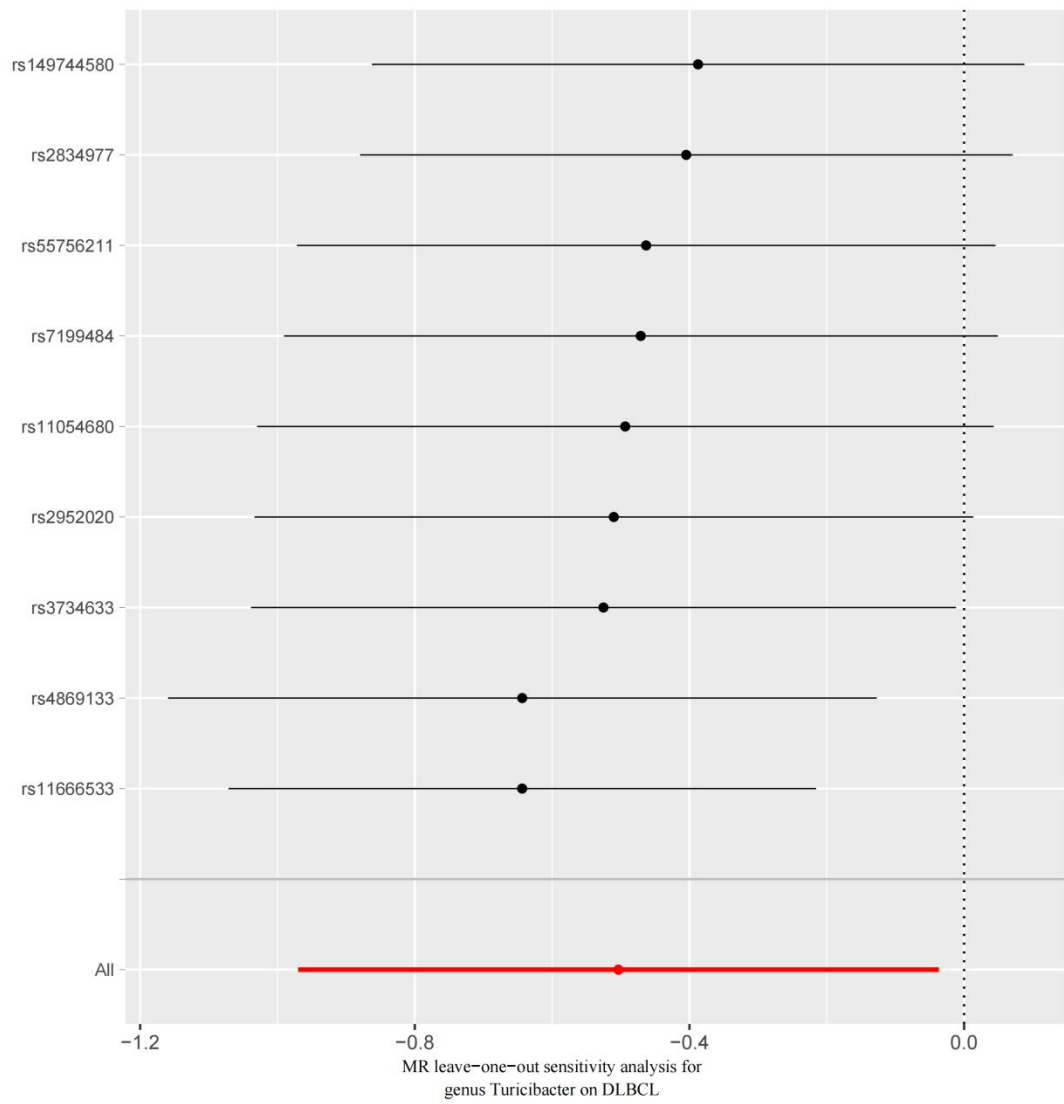

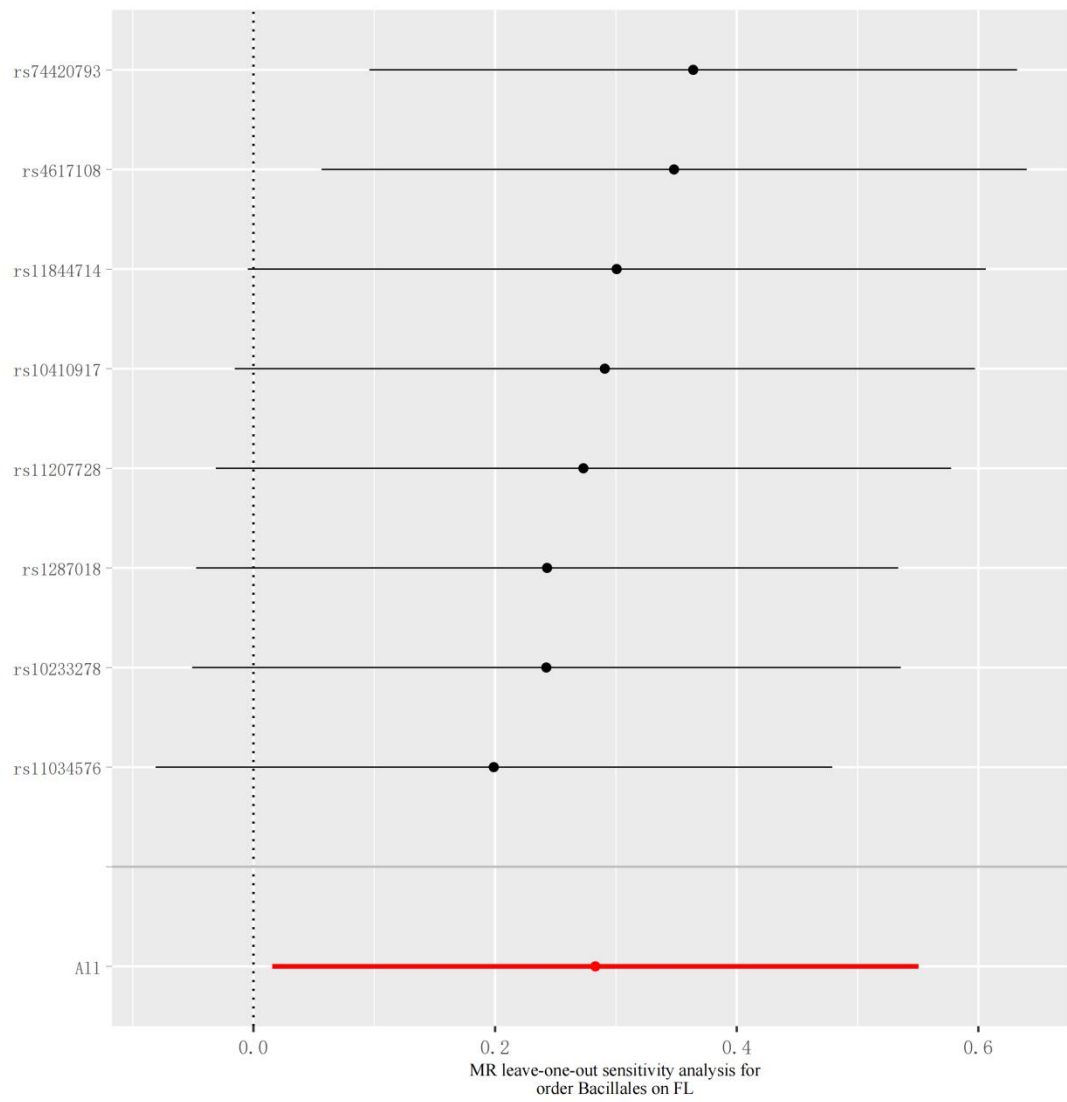

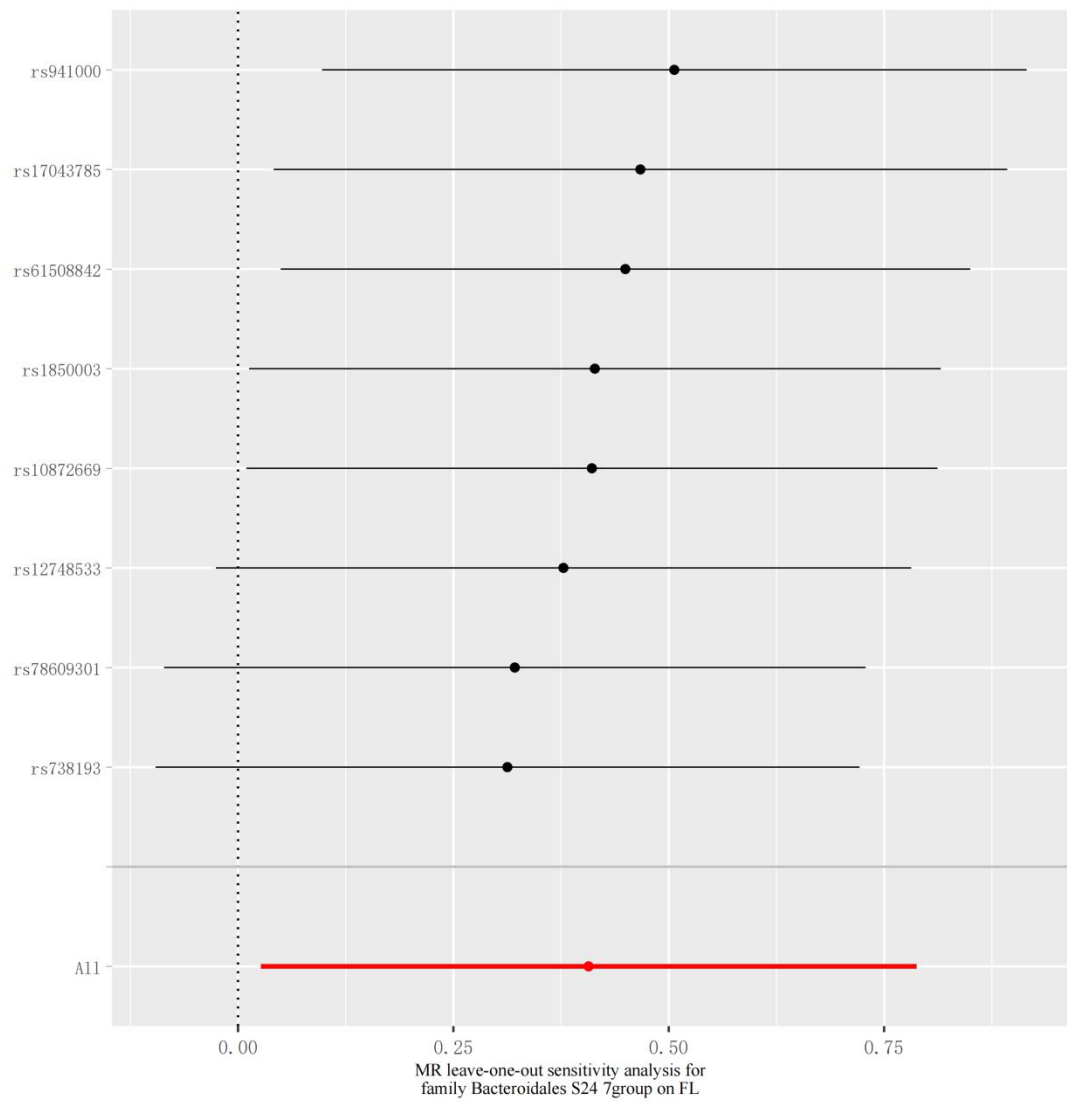

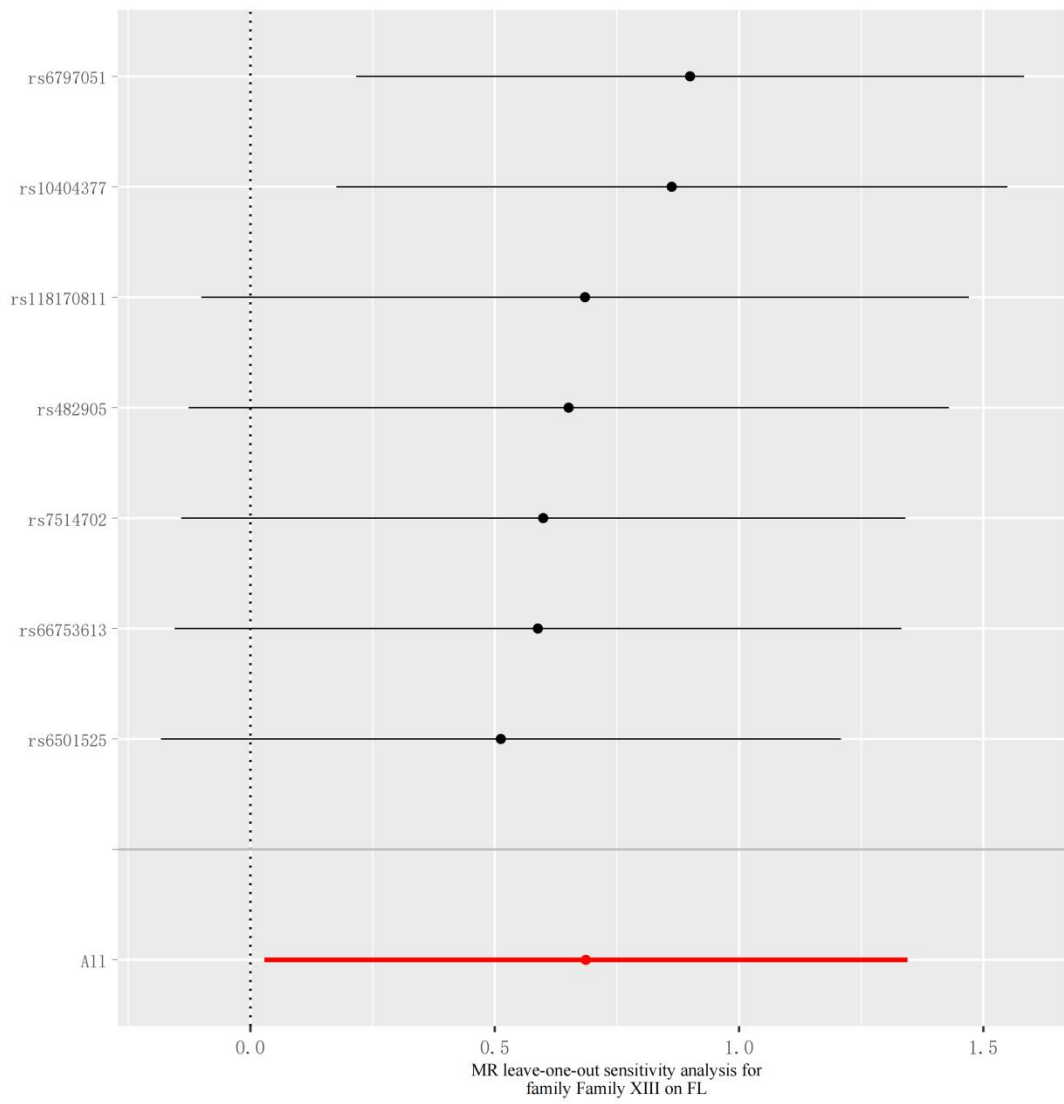

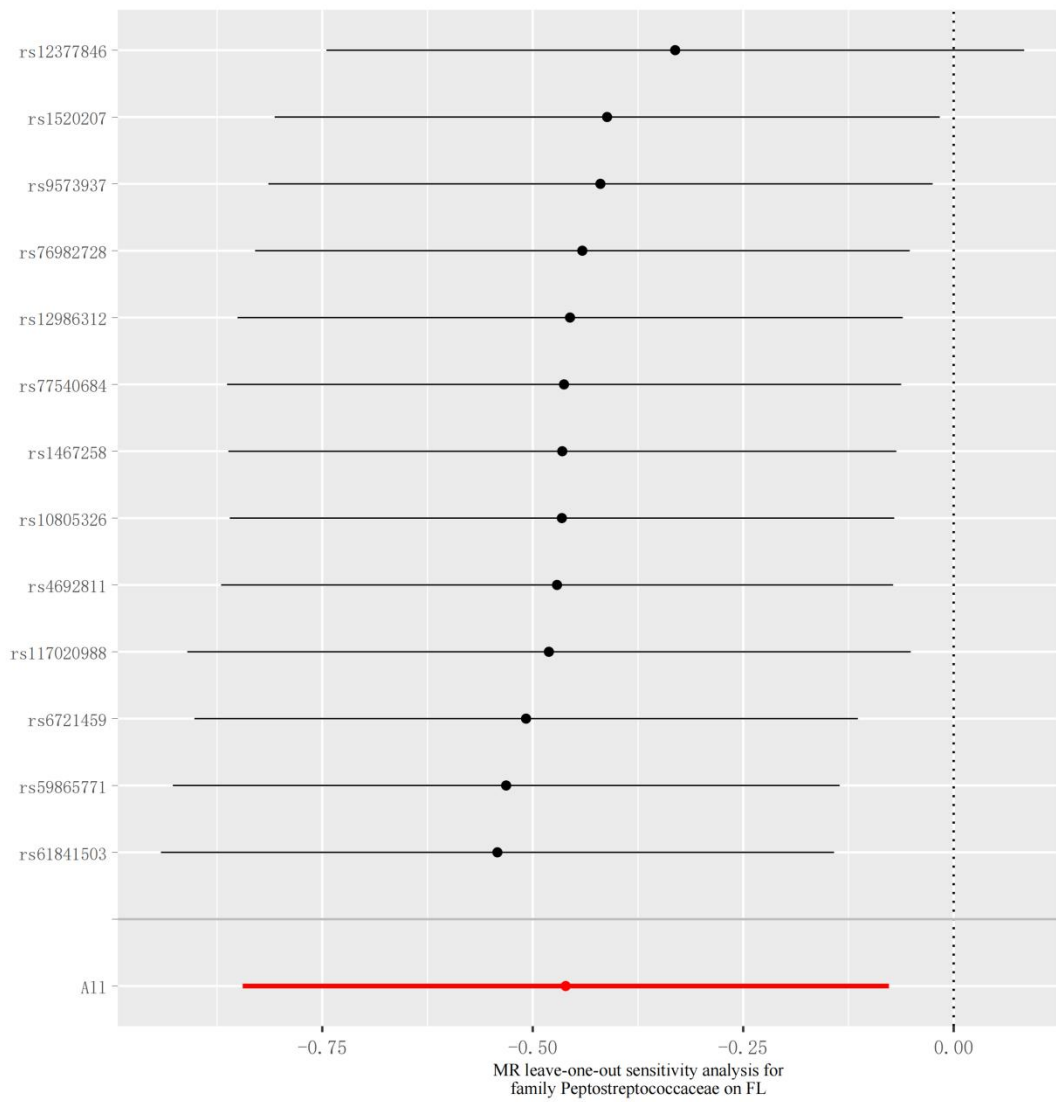

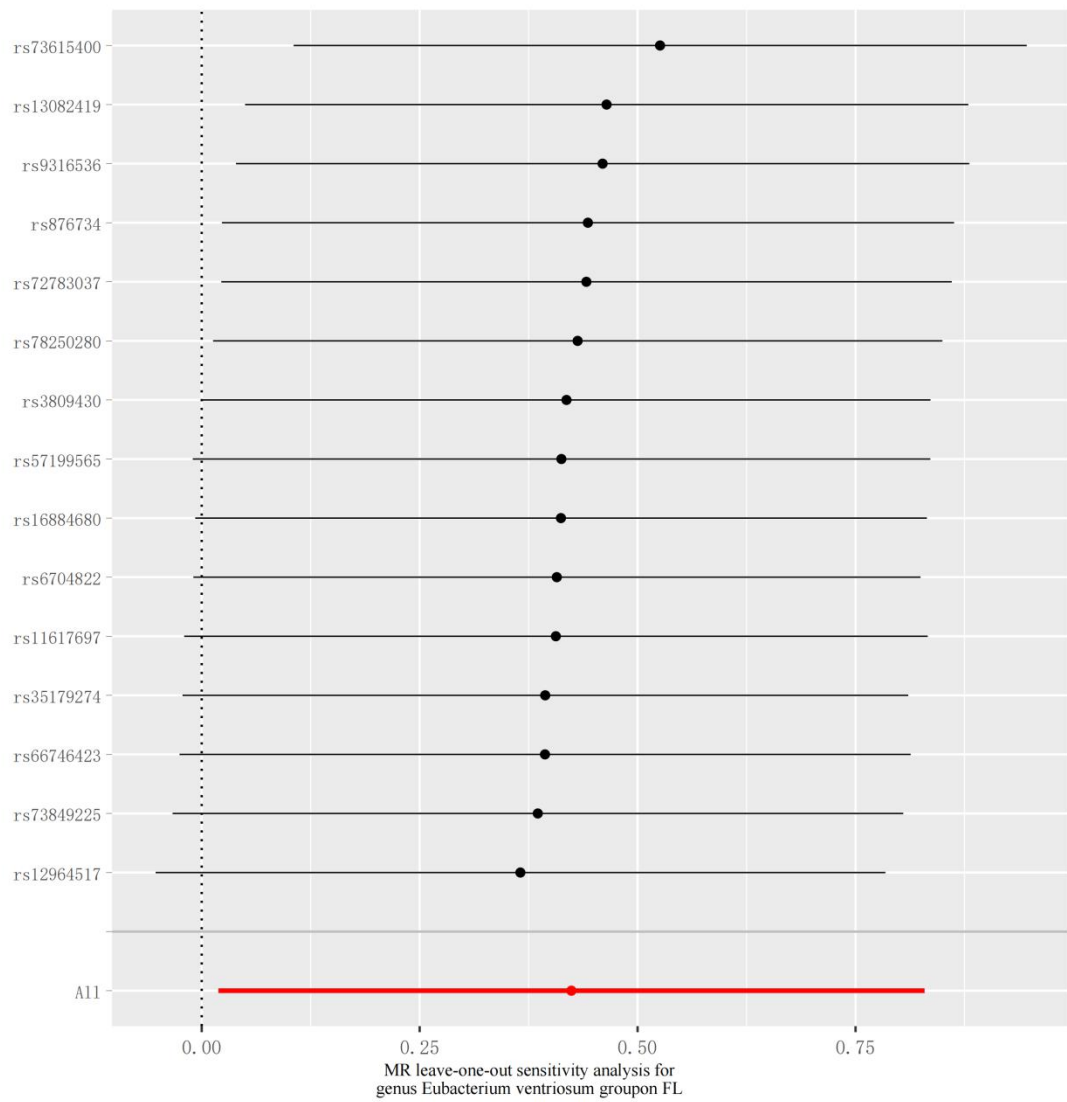

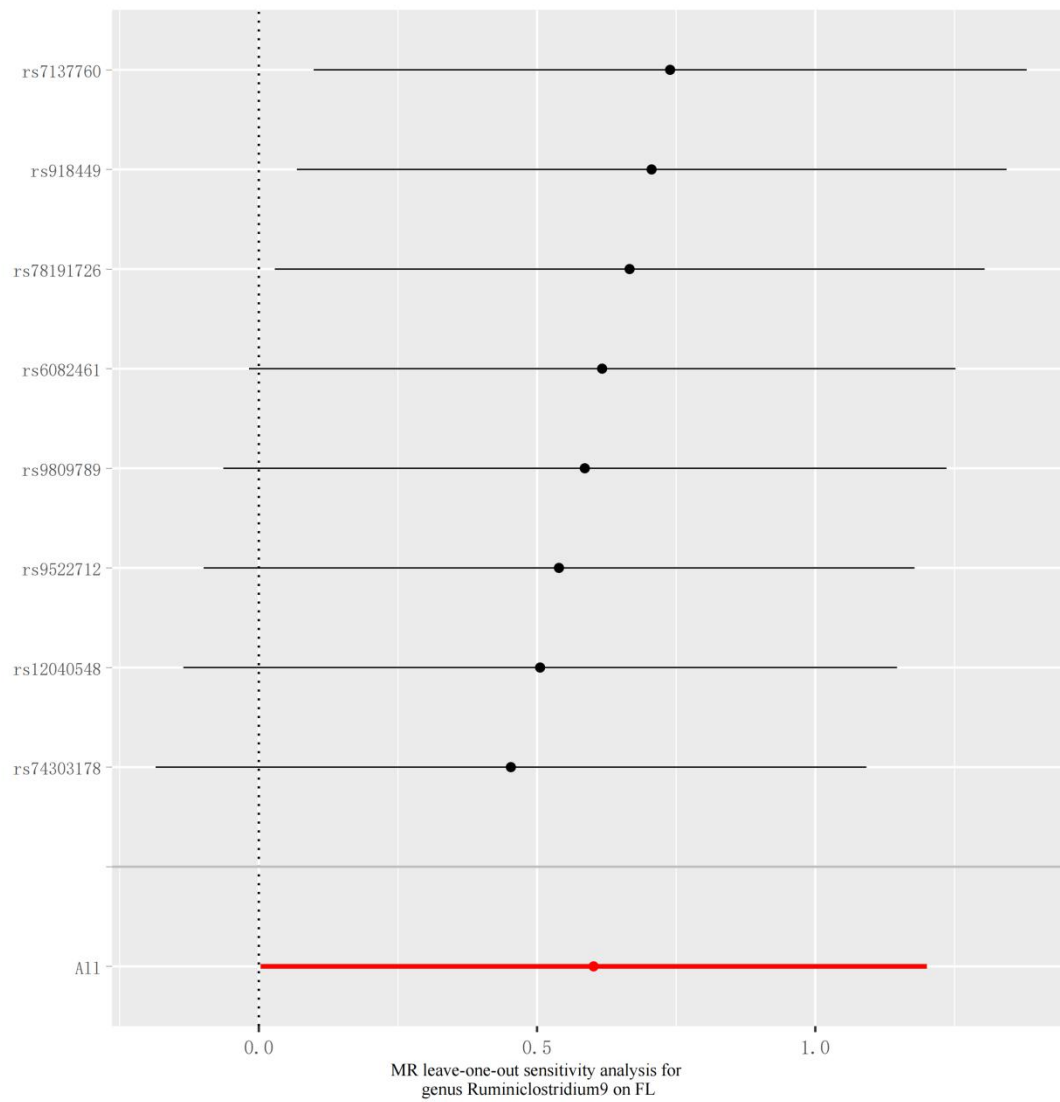

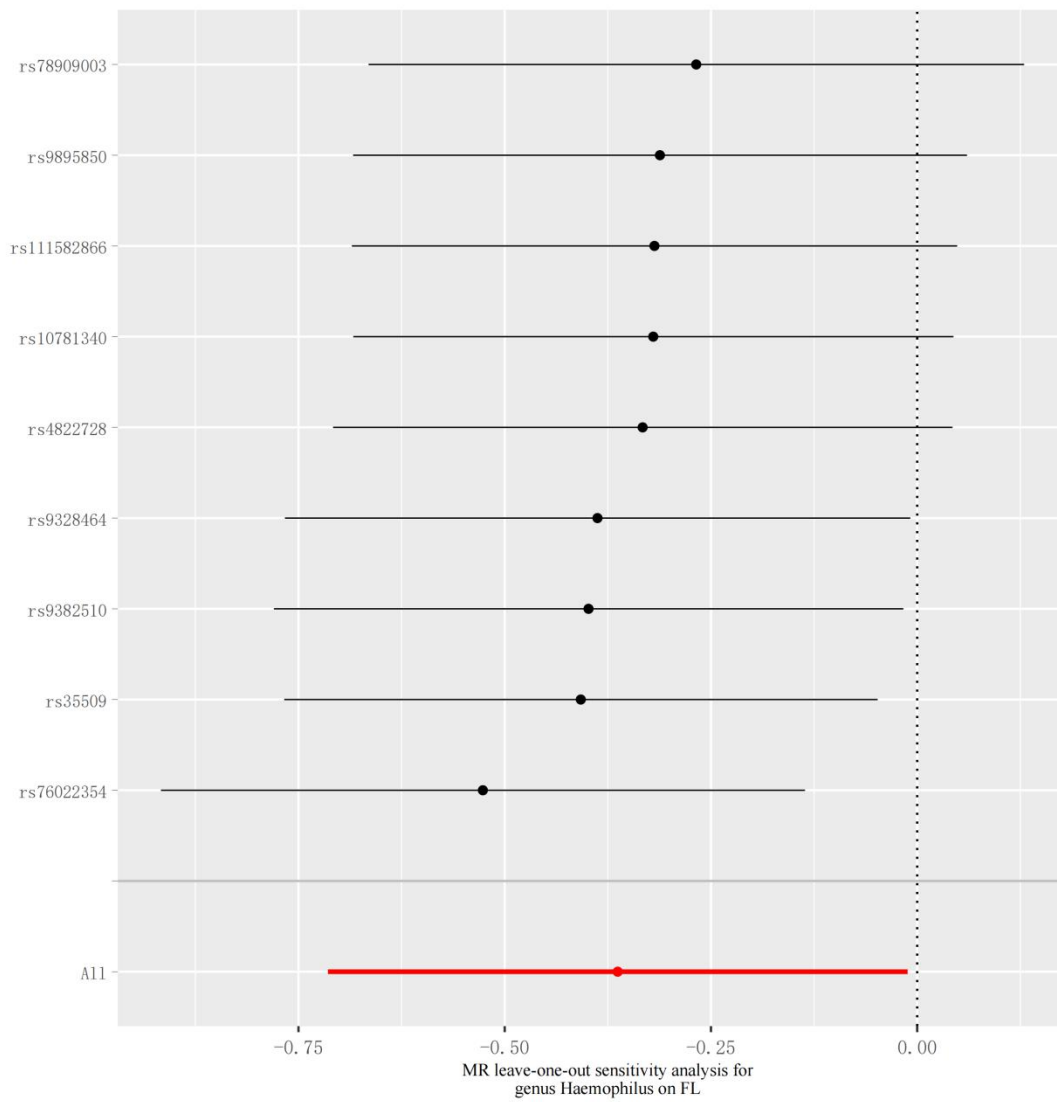

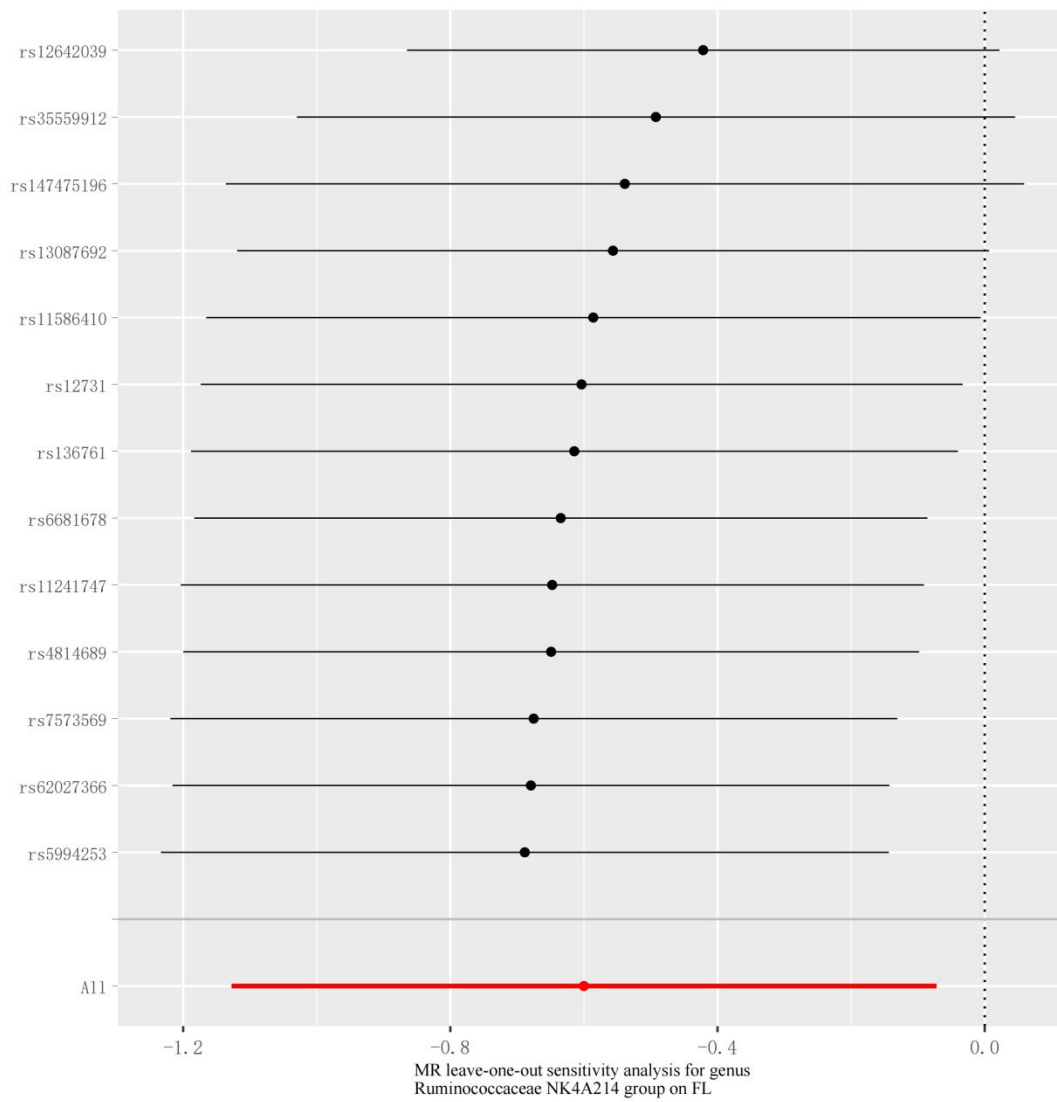

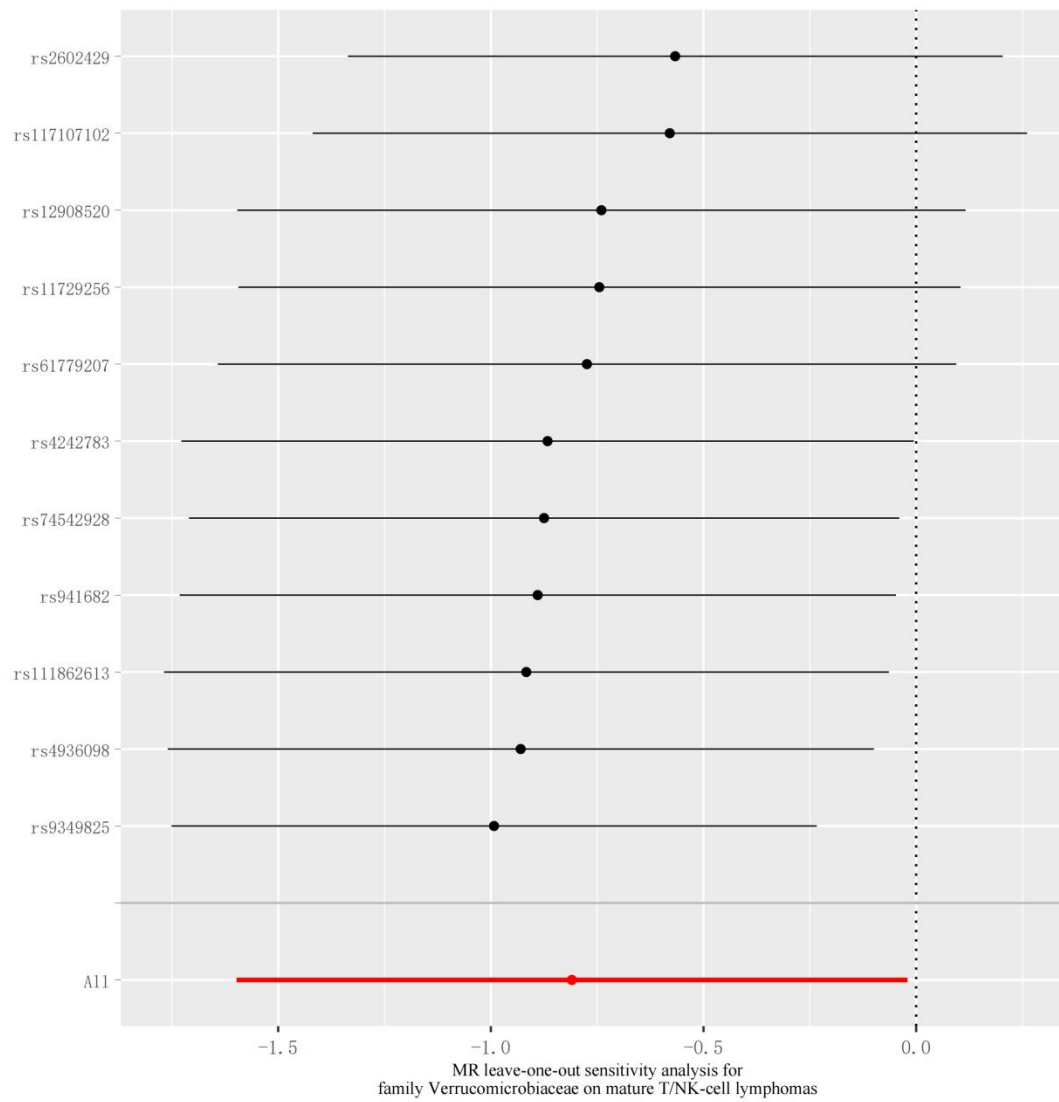

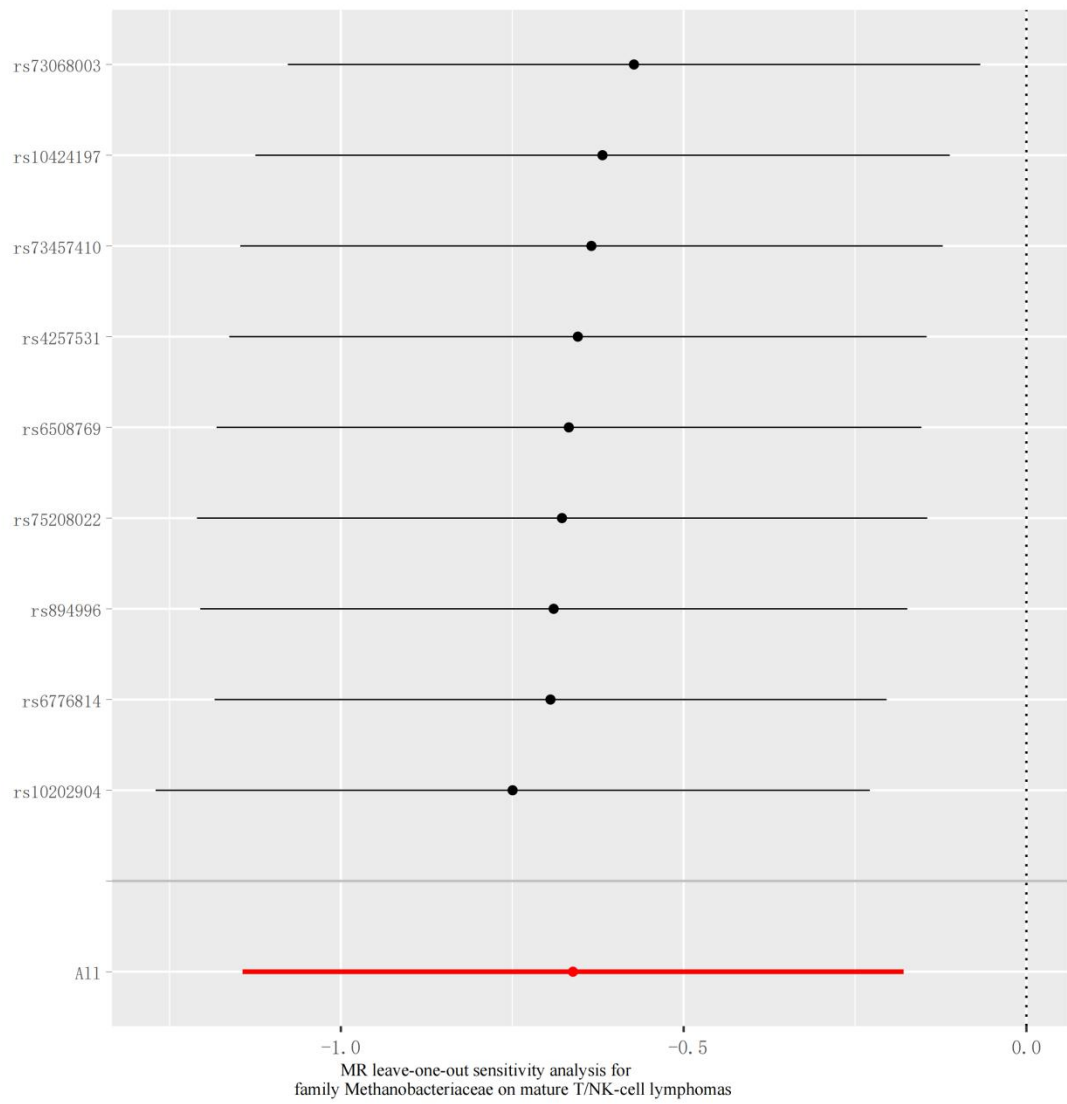

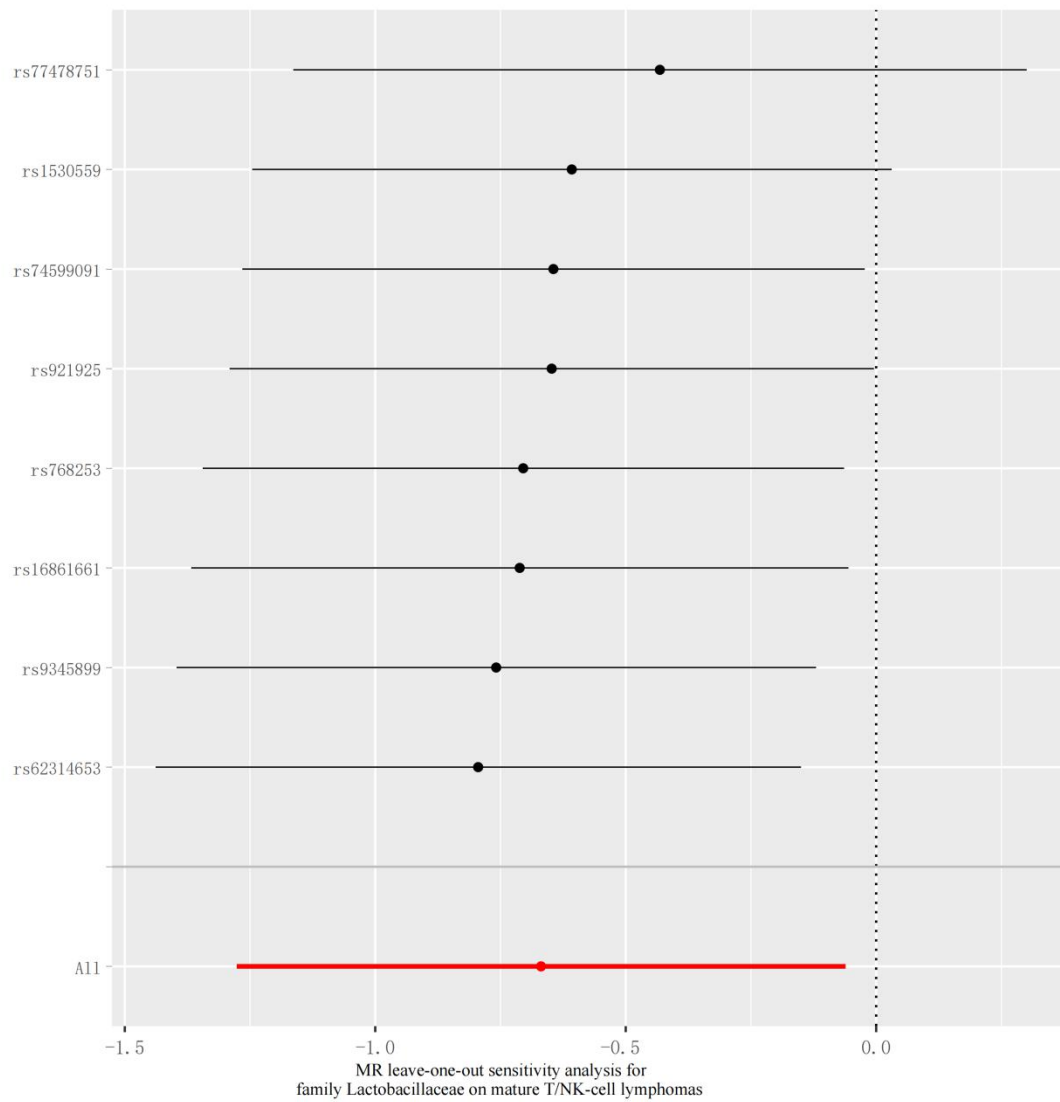

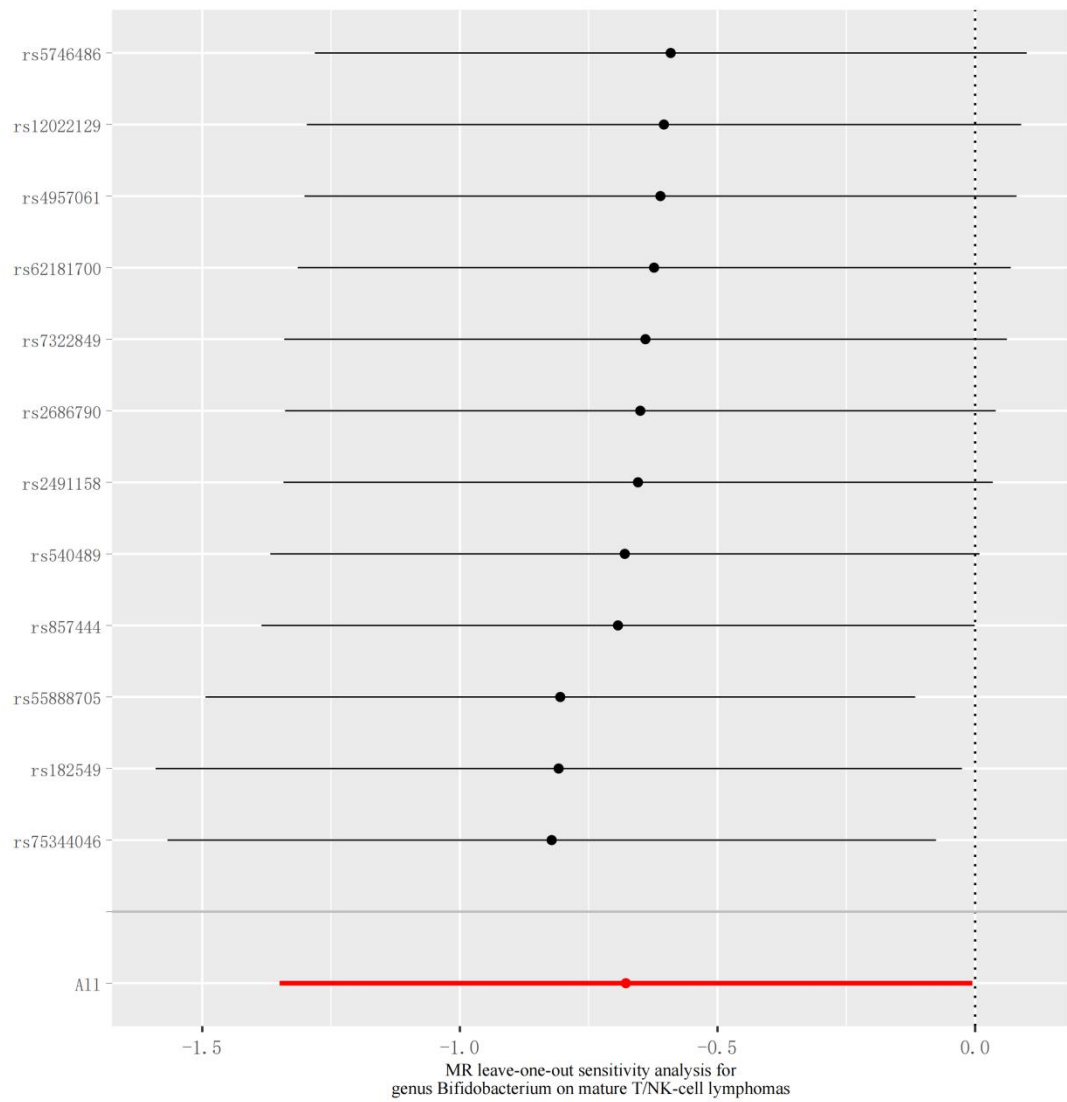

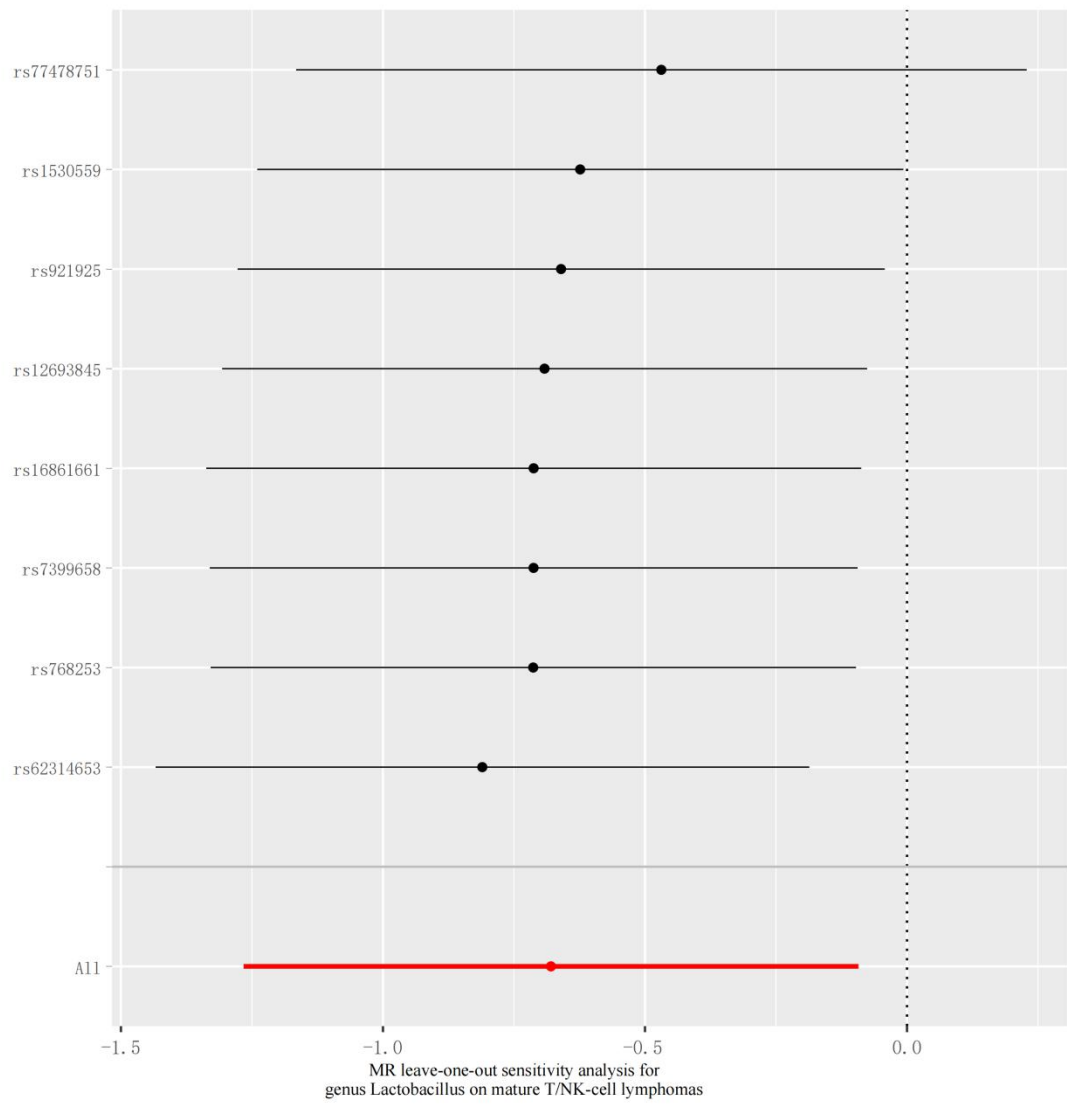

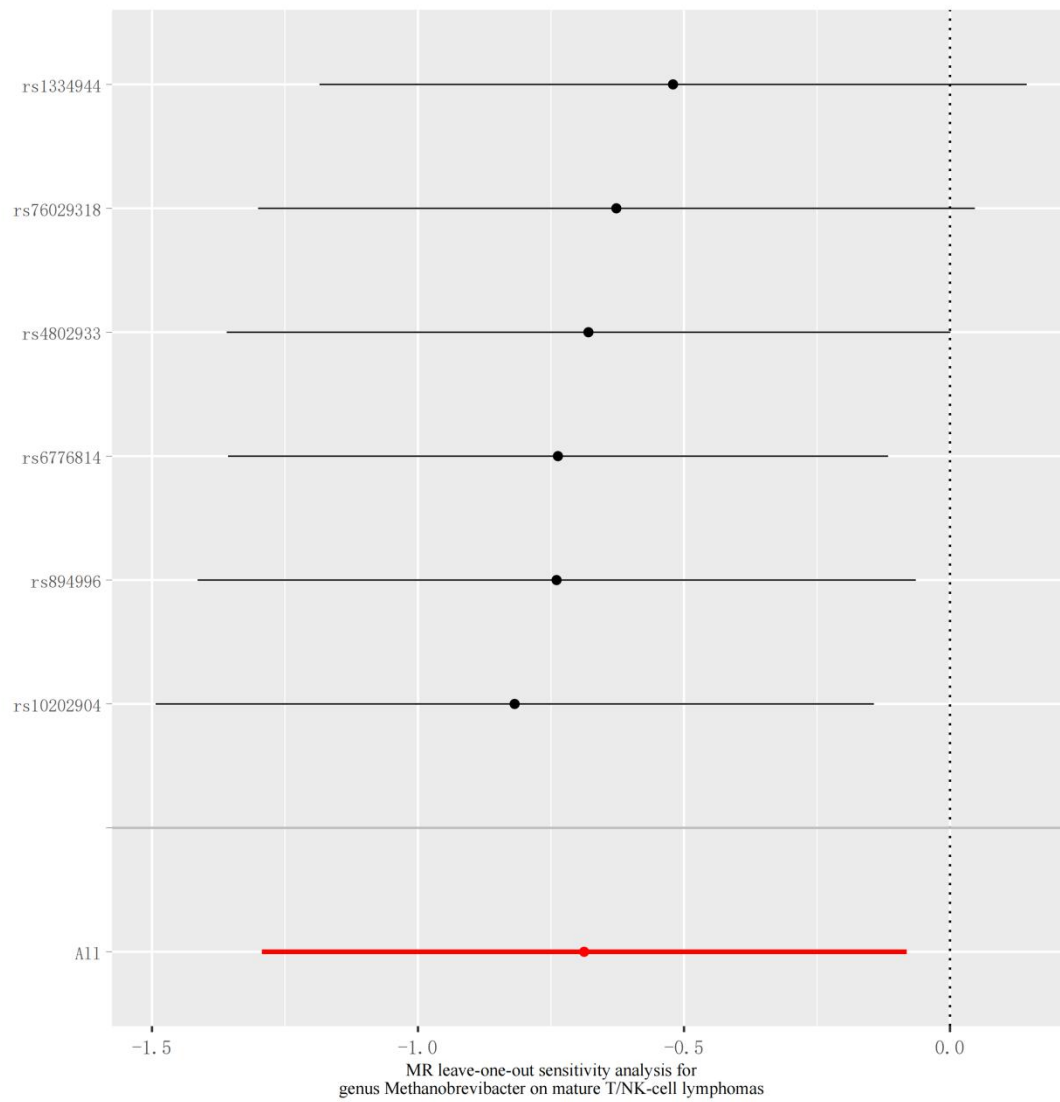

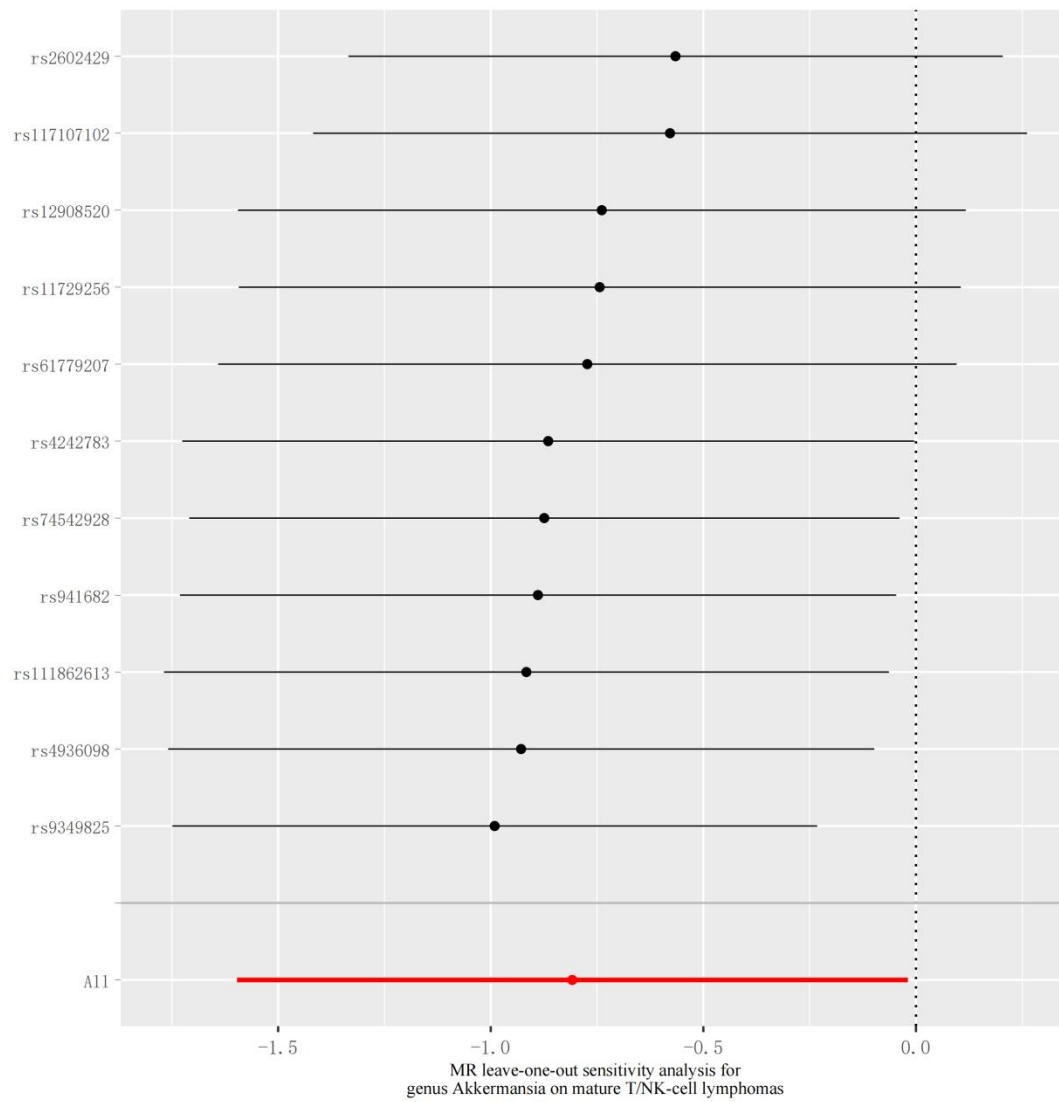

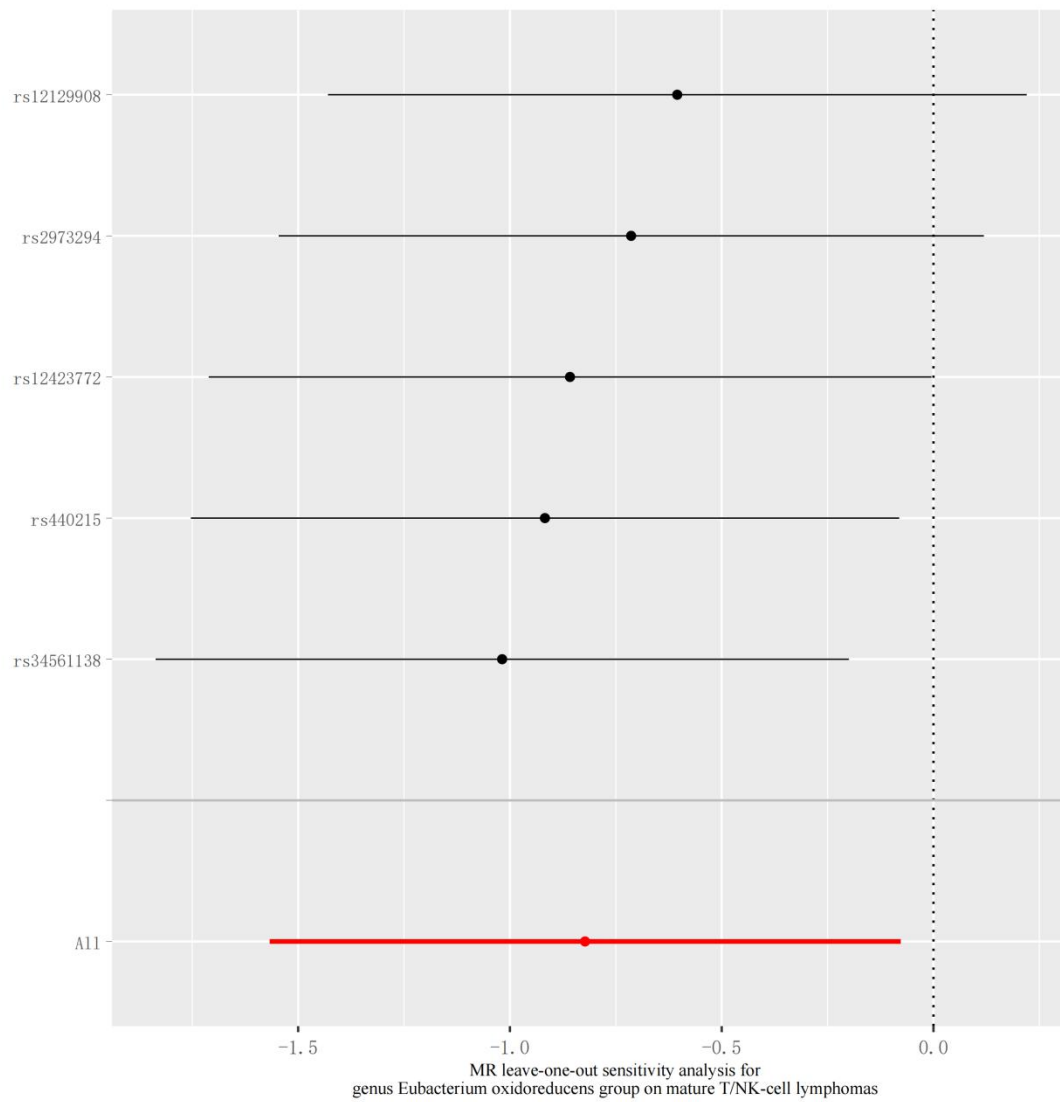

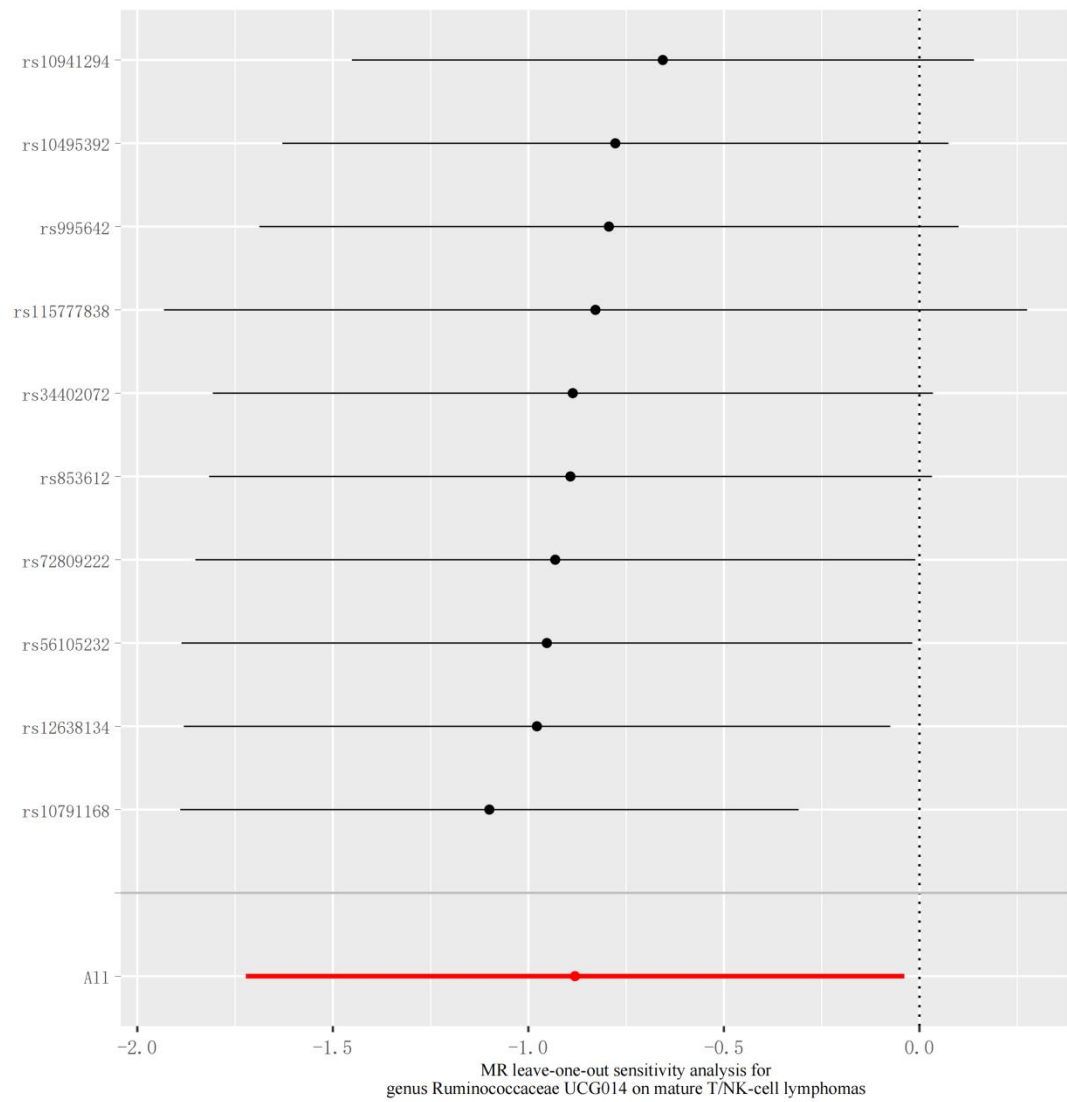

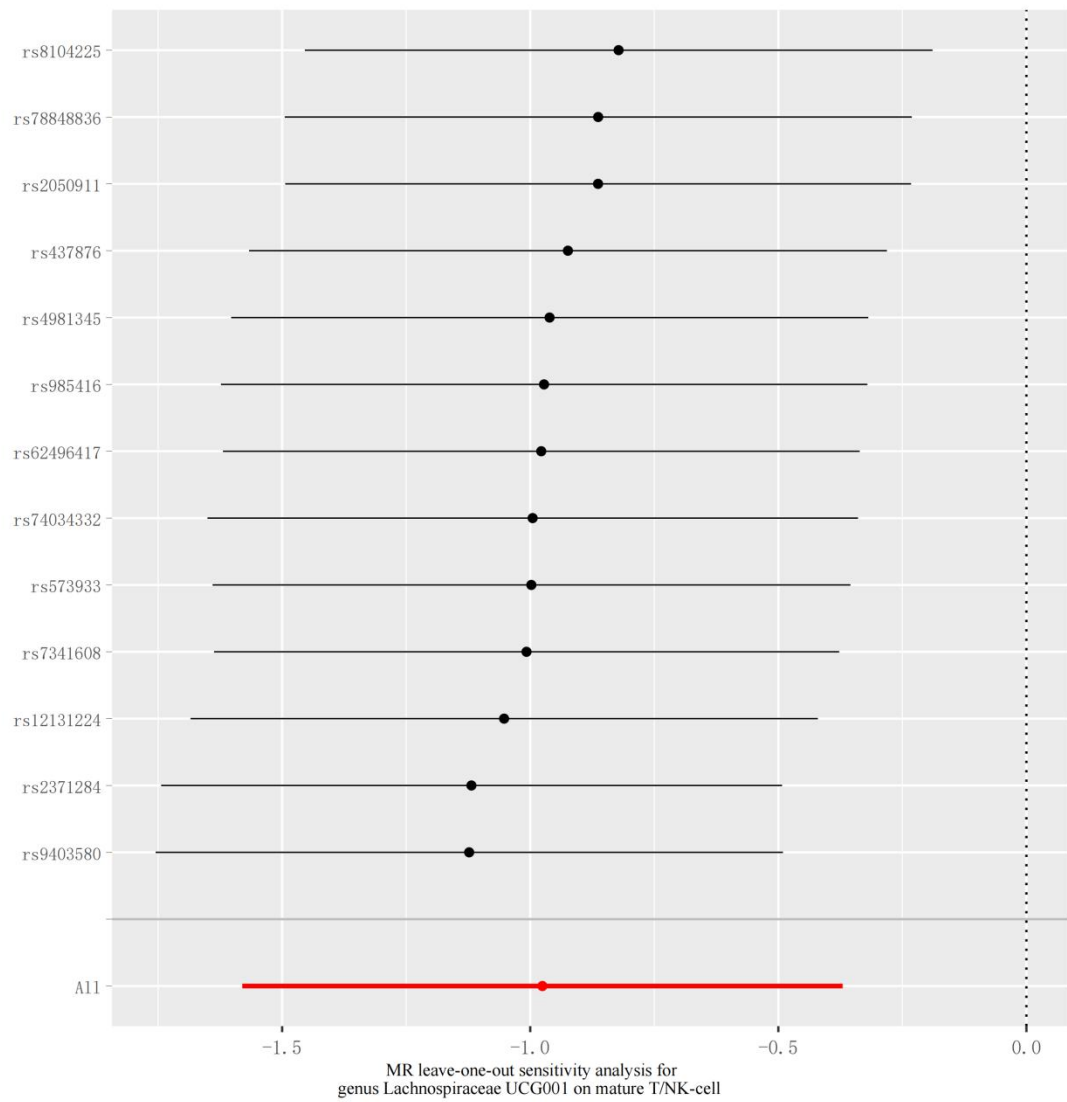

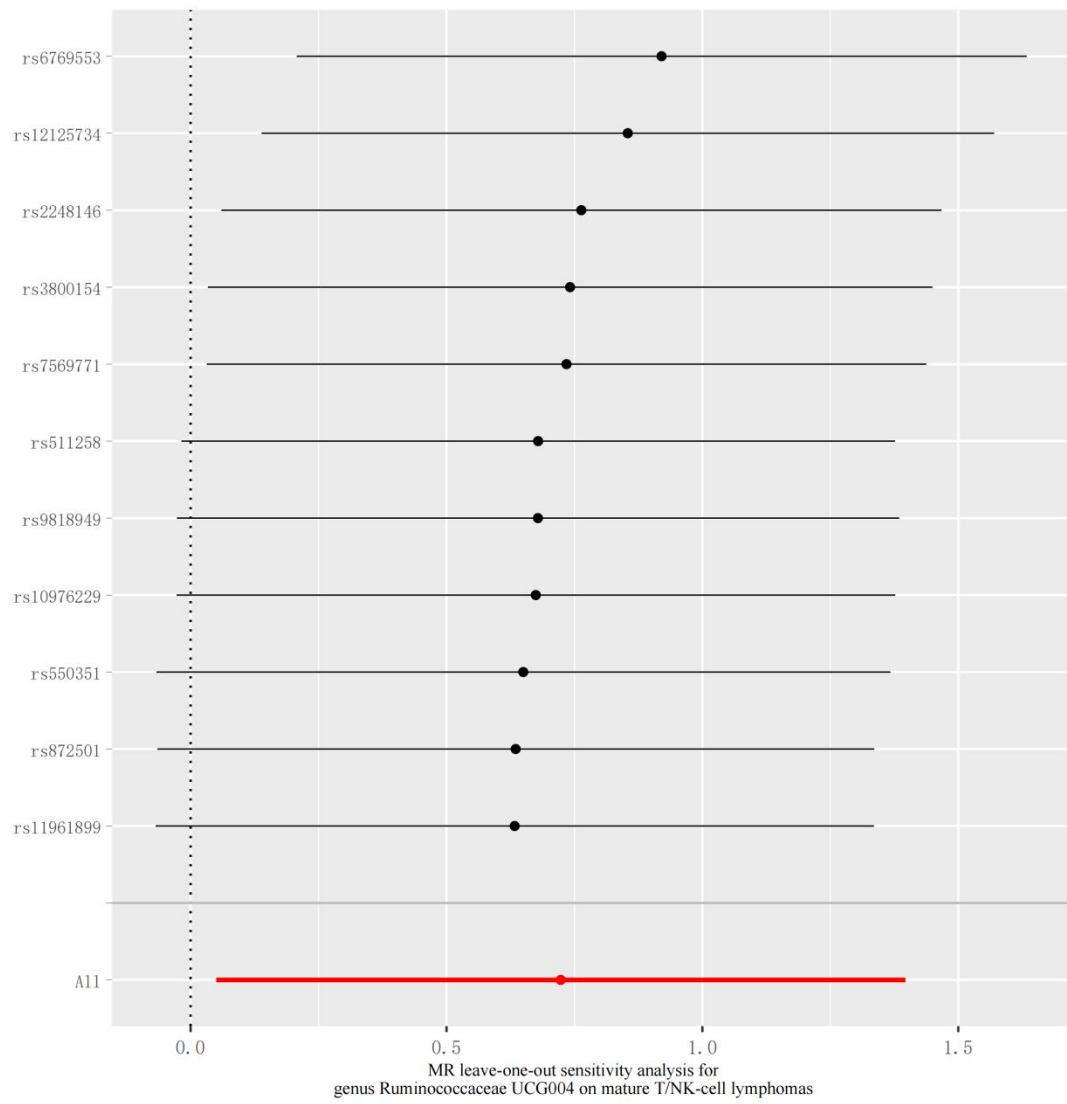

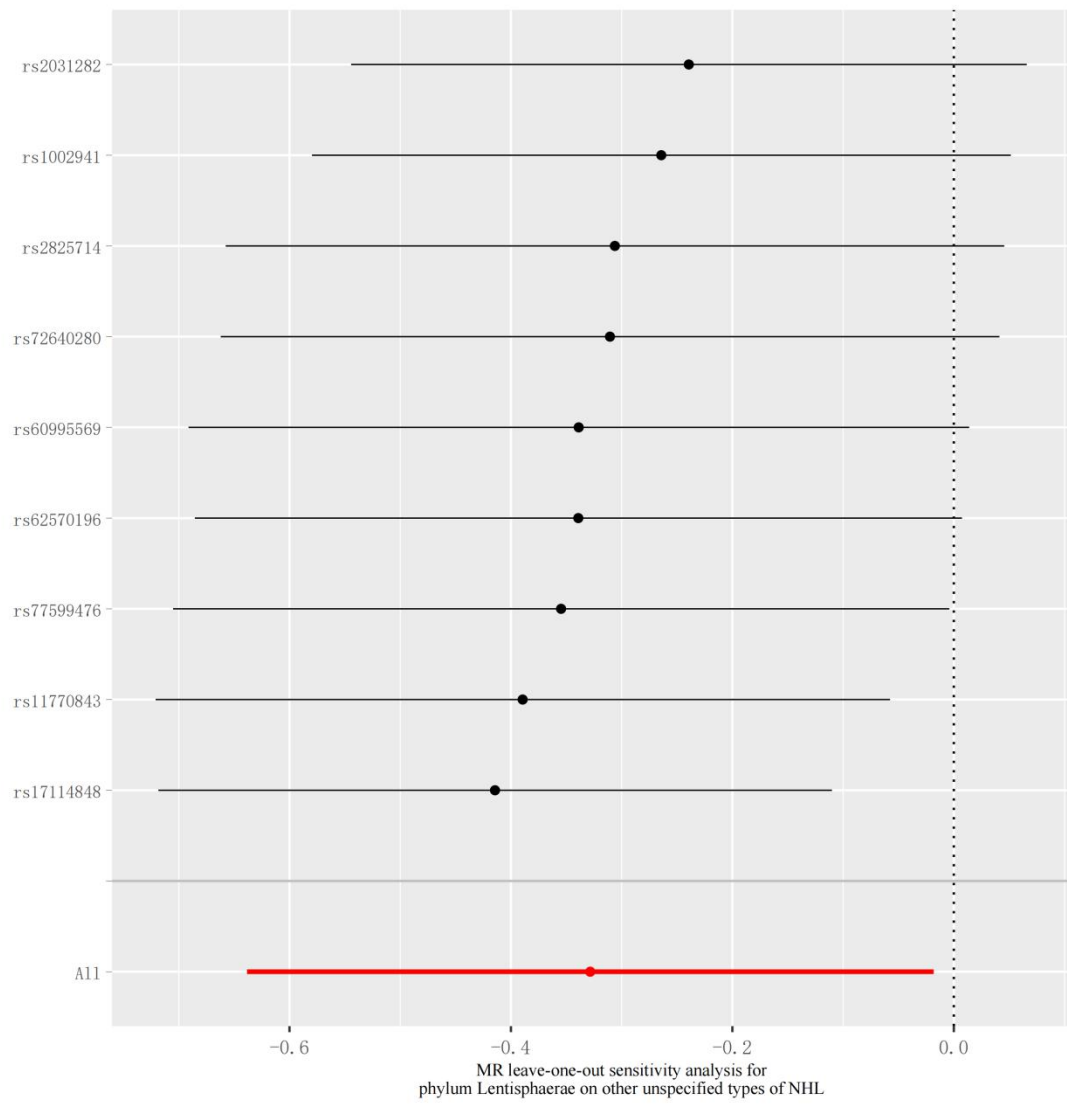

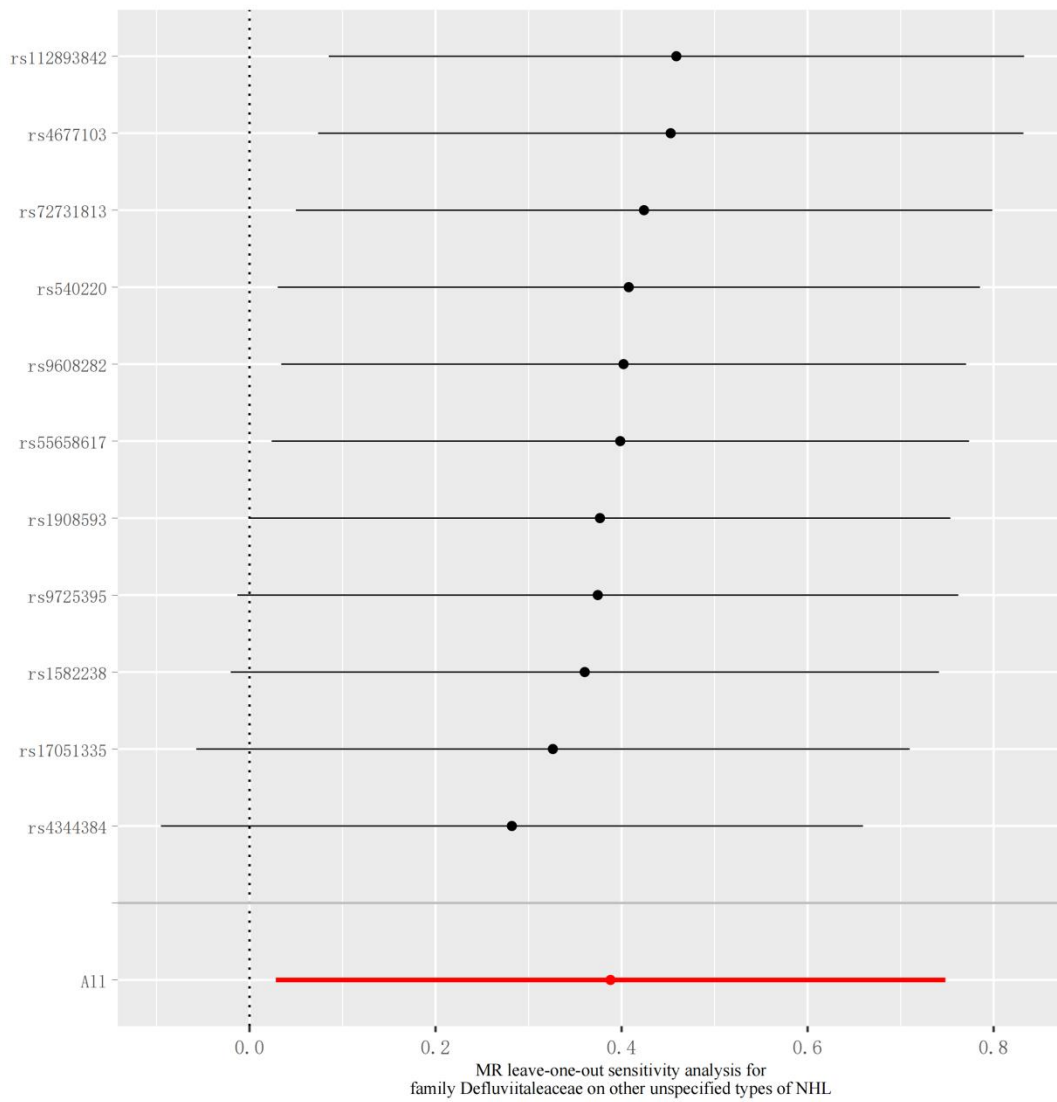

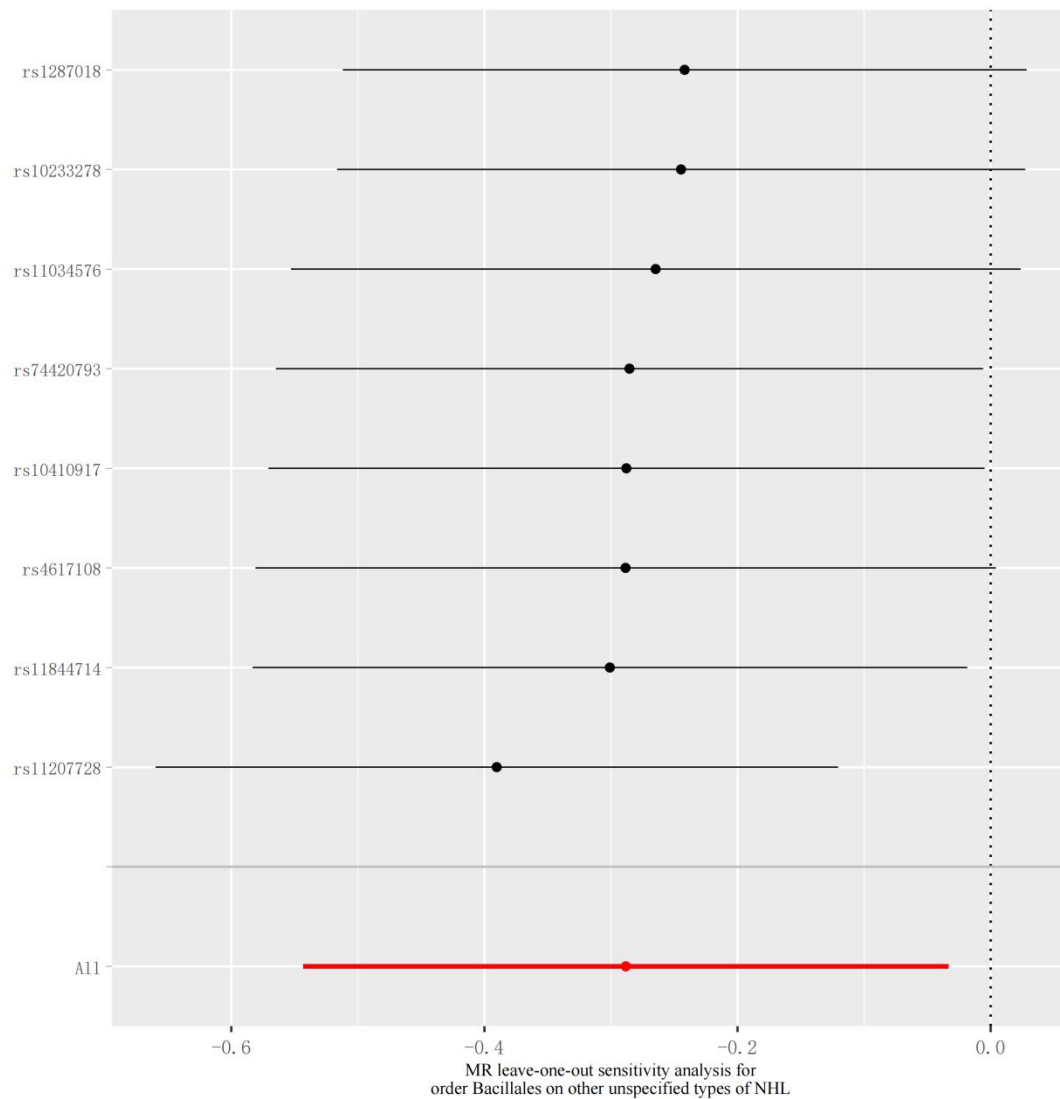

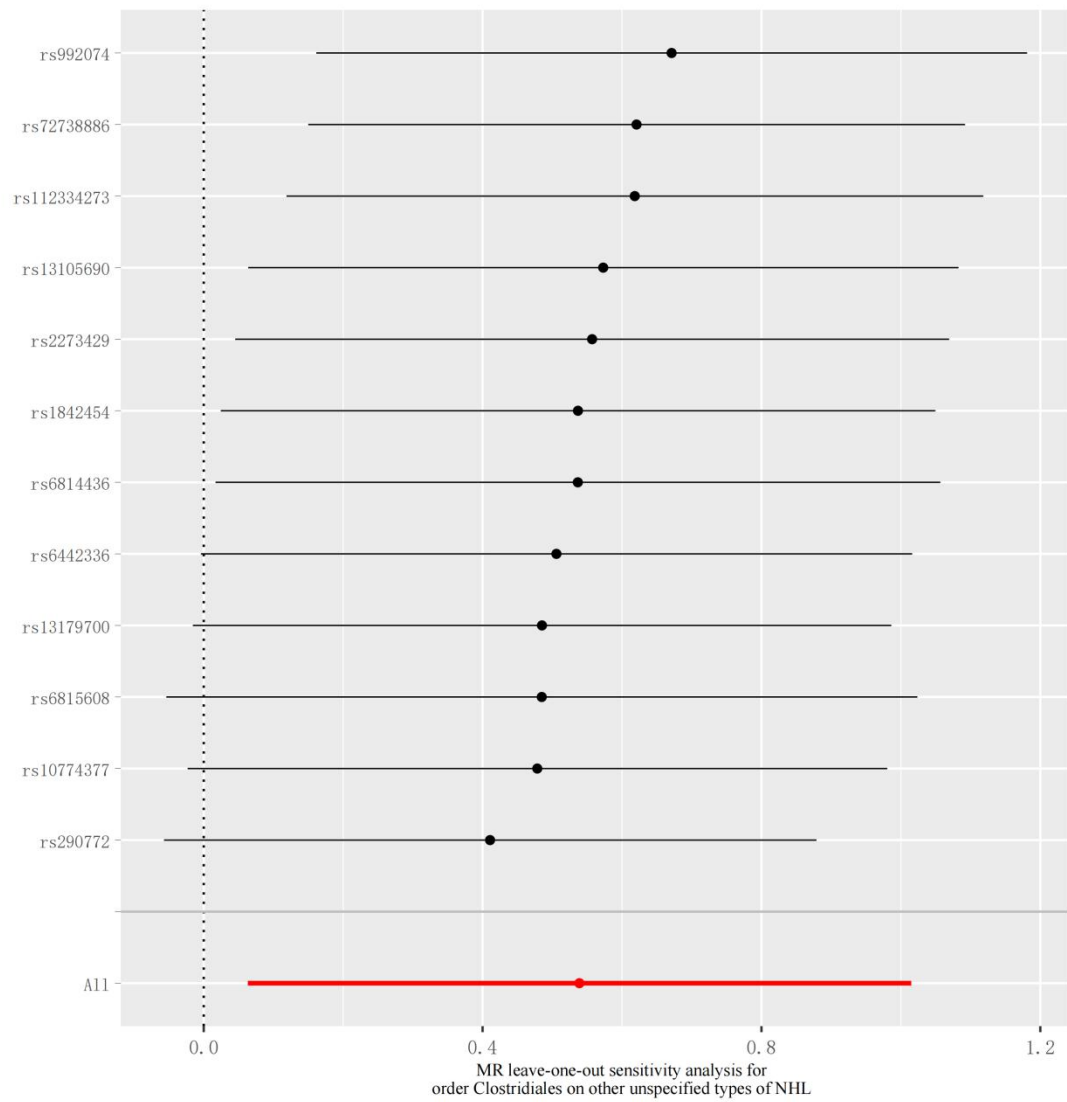

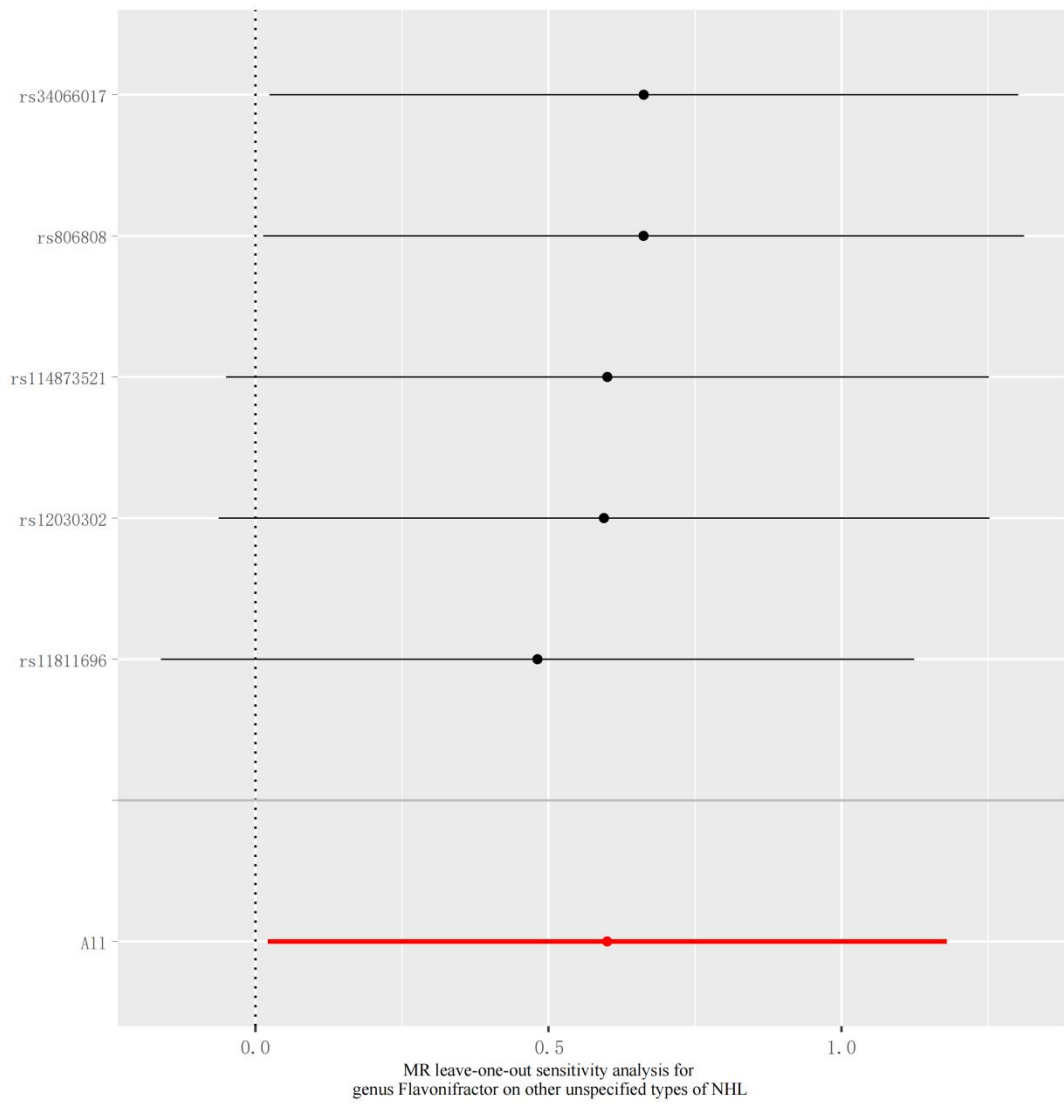

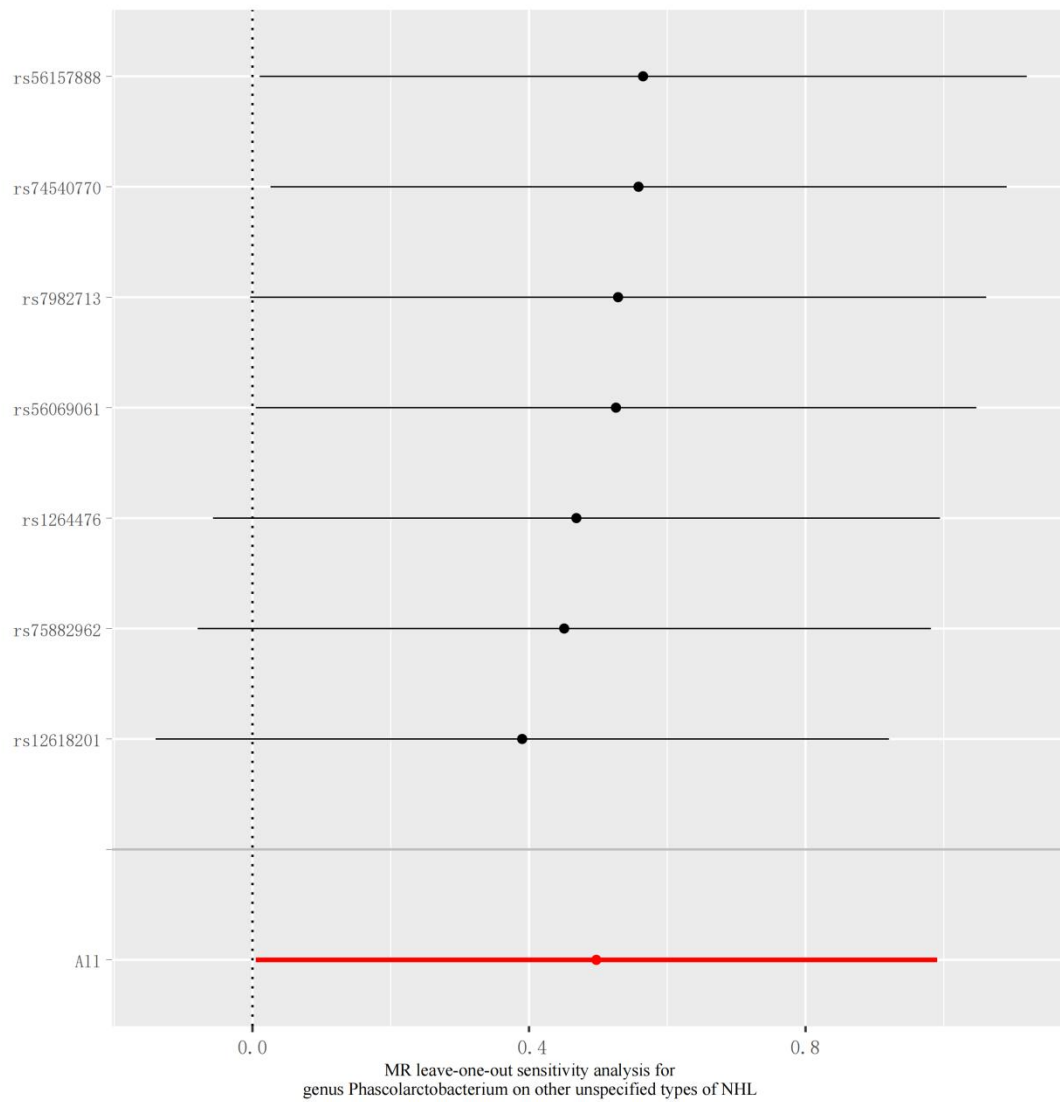

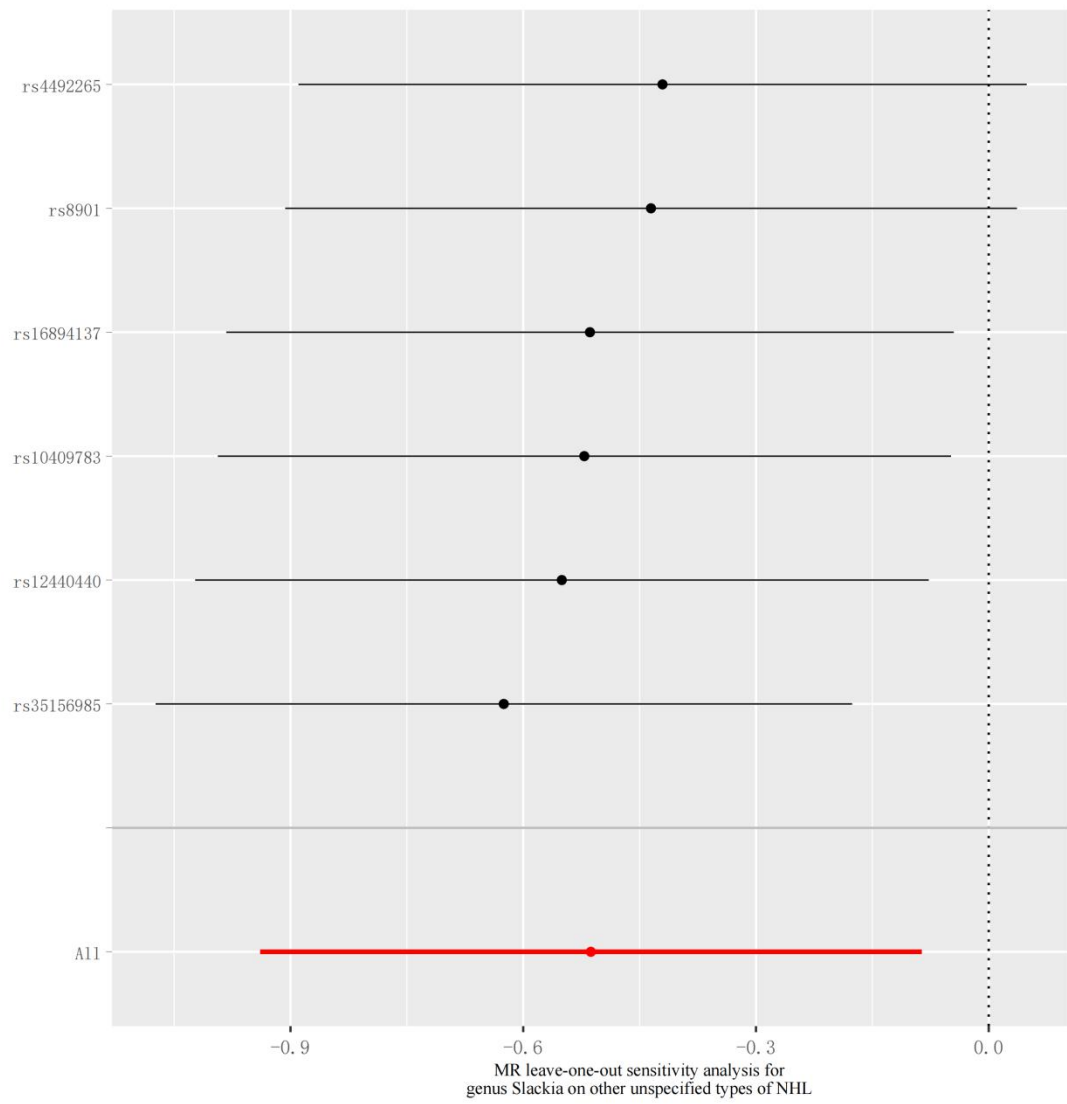

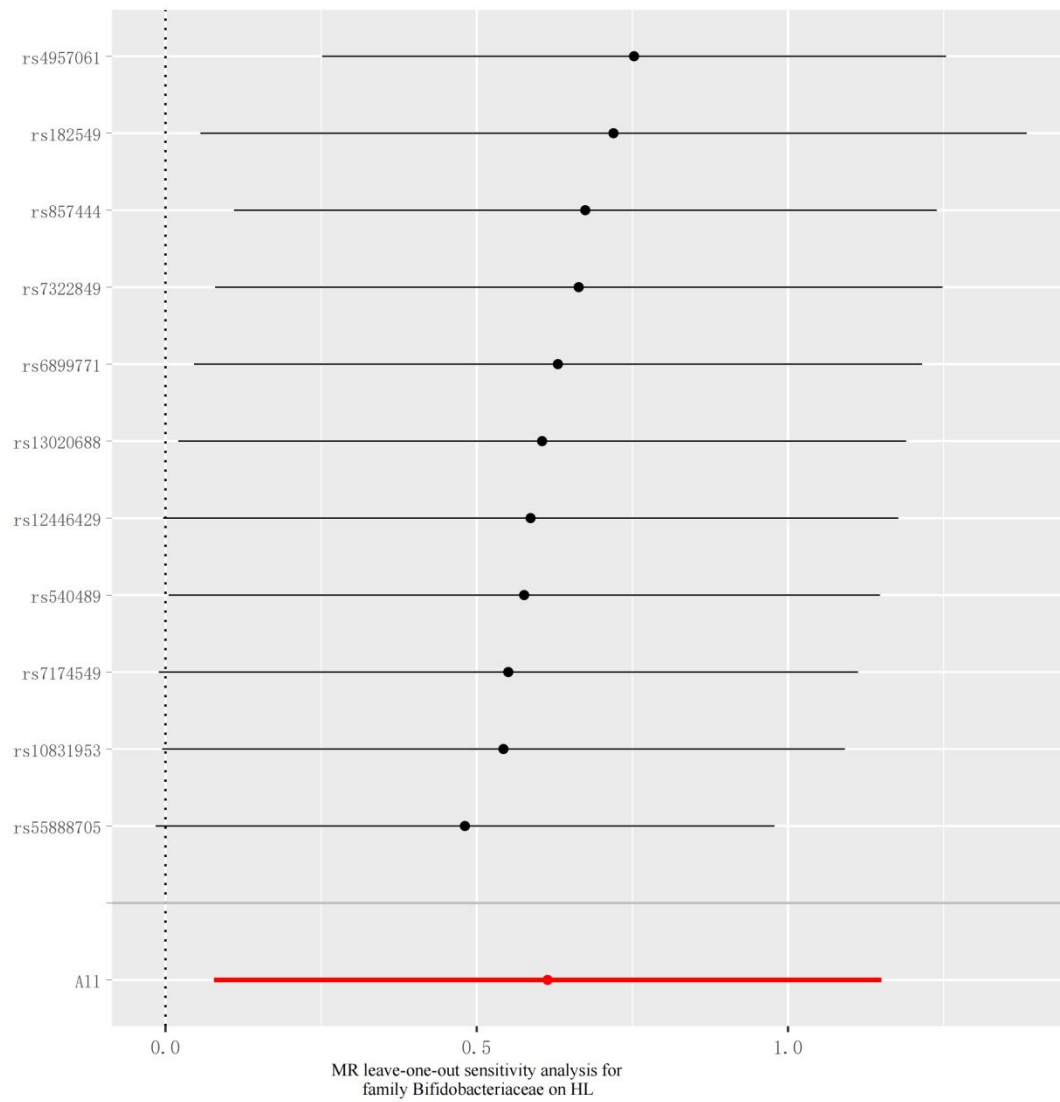

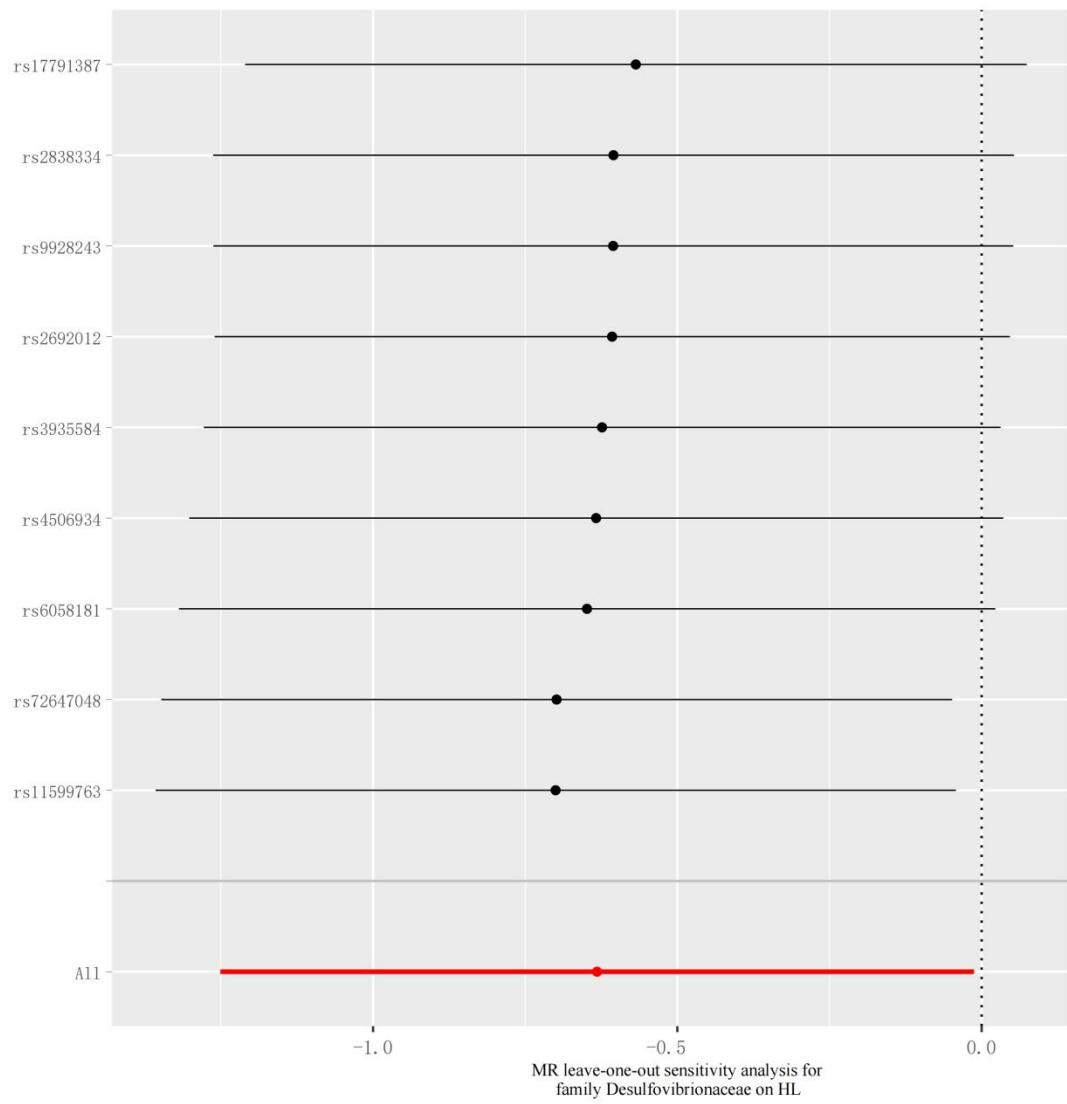

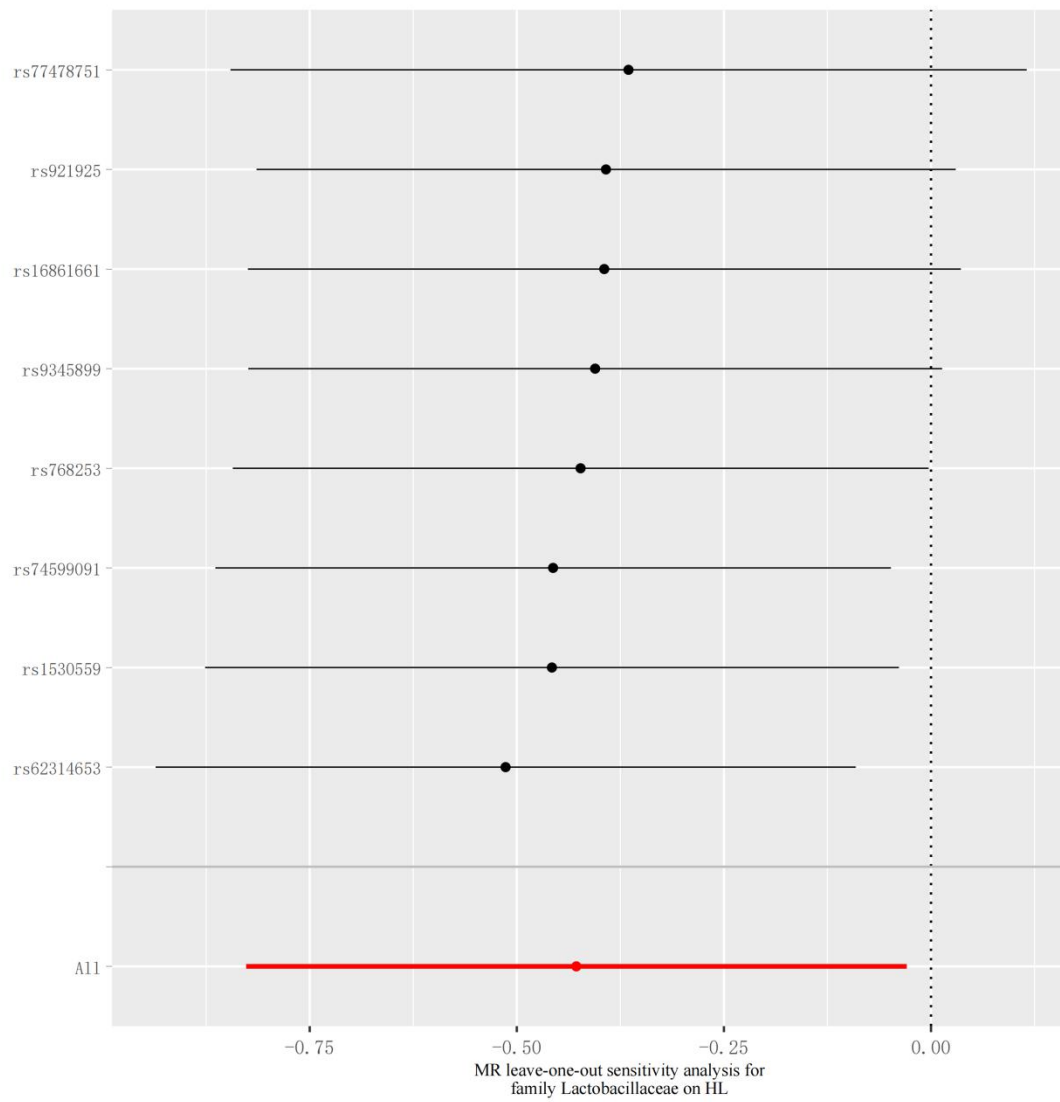

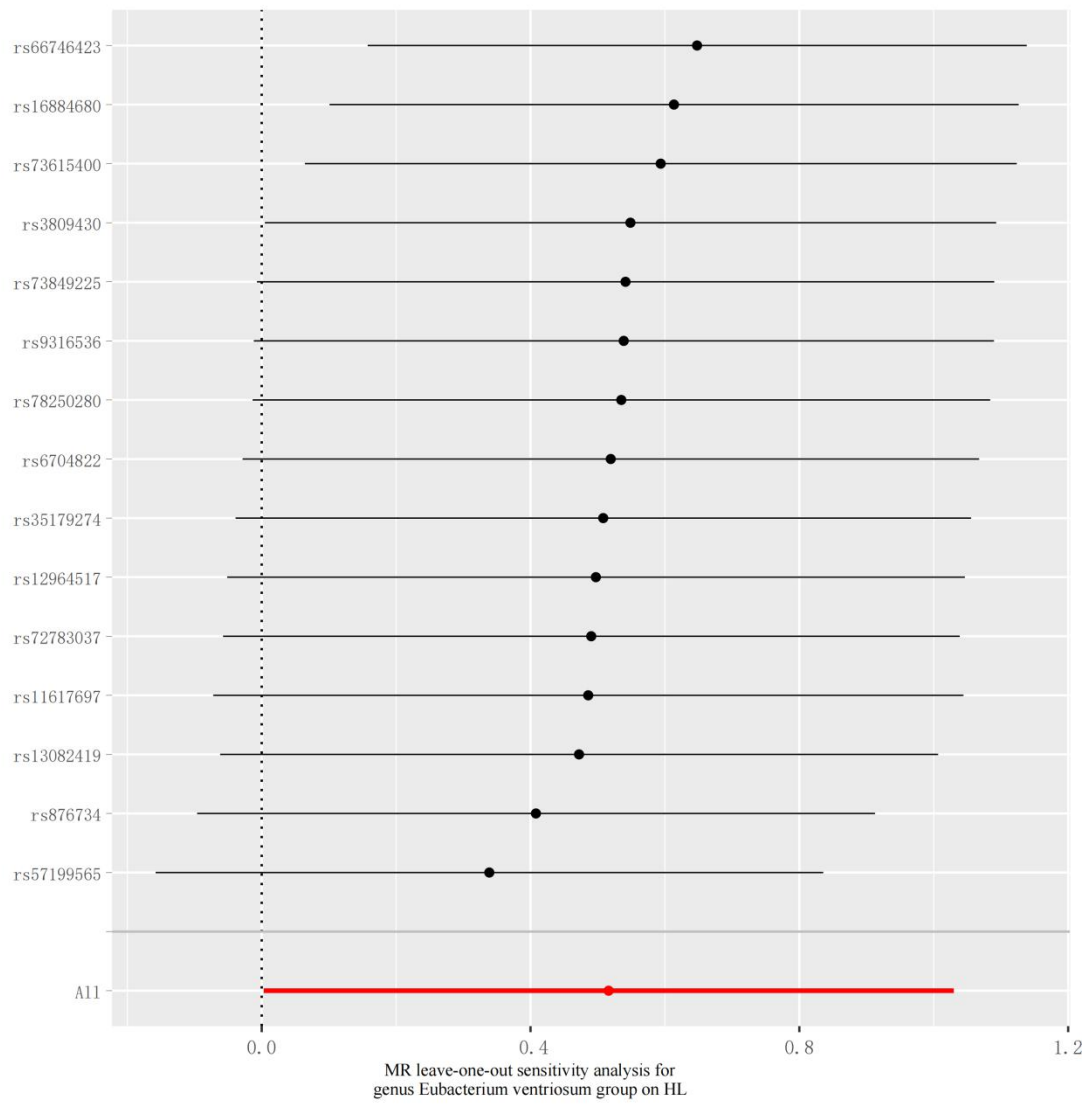

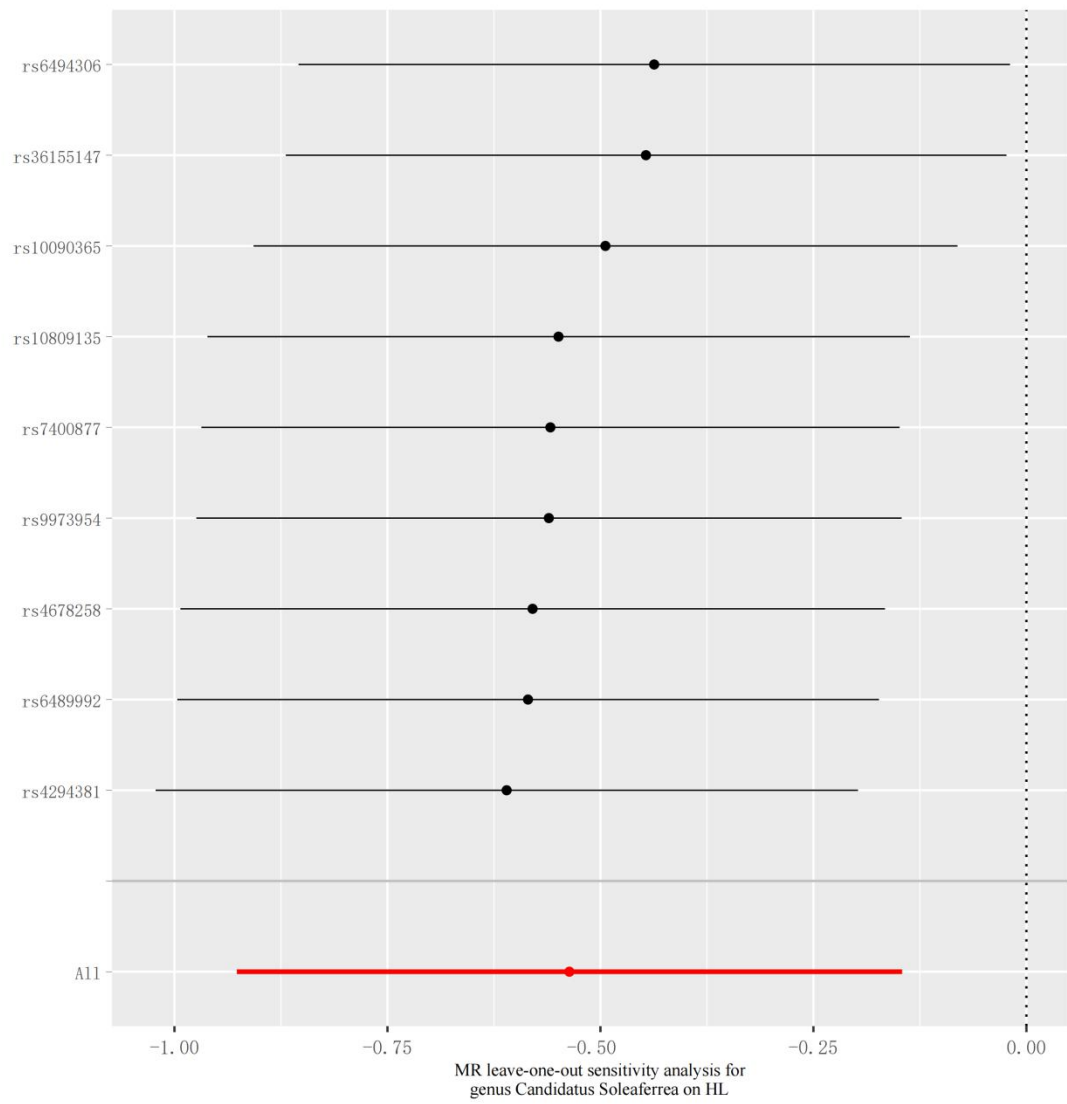

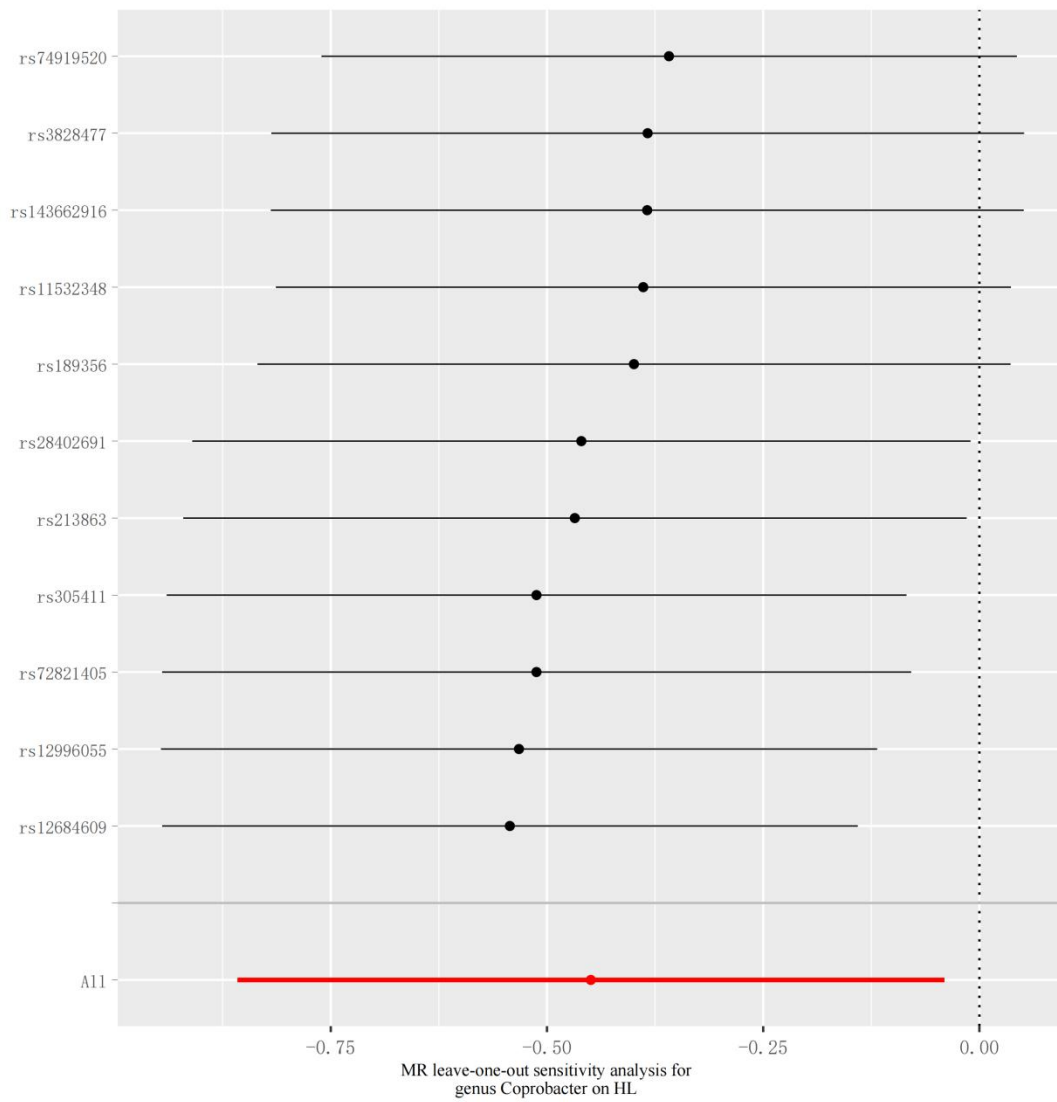

Supplement: Supplementary file 2 [file Image_2.pdf]
